# Effects of mHealth interventions to prescribe resistance training: a systematic review and meta-analysis of randomized controlled trials

**DOI:** 10.1186/s12966-025-01868-8

**Published:** 2025-12-22

**Authors:** Emily R. Cox, Sam Beacroft, Anna K. Jansson, Levi Wade, Mitch J. Duncan, David R. Lubans, Sara L. Robards, Manuel Leitner, Niklas Gutberlet, Ronald C. Plotnikoff

**Affiliations:** 1 Global Sport and Movement Collaborative, University of Newcastle, Callaghan, NSW 2308 Australia; 2 Global Sport and Movement Collaborative, Hunter Medical Research Institute, New Lambton Heights, Newcastle, NSW 2305 Australia; 3 School of Biomedical Sciences and Pharmacy, University of Newcastle, Callaghan, NSW 2308 Australia; 4 School of Heath Sciences, University of Newcastle, Callaghan, NSW 2308 Australia; 5 Nutrition and Metabolic Health Program, Hunter Medical Research Institute, New Lambton Heights, Newcastle, NSW 2305 Australia; 6 School of Medicine & Public Health, College of Health, Medicine, and Wellbeing, The University of Newcastle, University Drive, Callaghan, NSW 2308 Australia; 7 Faculty of Sport and Health Sciences, University of Jyväskylä, Jyväskylä, Finland; 8 Institute of Sports and Sports Science, Karlsruhe Institute of Technology, Karlsruhe, Germany

**Keywords:** Mobile applications, Muscle strength, Physical fitness, Physical activity, Technology

## Abstract

**Background:**

This review evaluated the efficacy of resistance training mHealth interventions for improving neuromuscular fitness and resistance training participation. It also explored how resistance training is prescribed through mHealth, and the theoretical frameworks and behavior change techniques (BCTs) employed.

**Methods:**

MEDLINE (OVID), Embase (OVID), Emcare (OVID), SPORTDiscus, Web of Science, Scopus and Cochrane (CINAHL) were searched from January 2010 to February 2025. Randomized controlled trials published in English, targeting adults, that prescribed resistance training via an mHealth platform and measured at least one outcome of neuromuscular fitness or resistance training participation were included.

**Results:**

From the 12,059 records identified, 32 RCTs were included. mHealth-delivered resistance training interventions produced a small, statistically significant improvement in neuromuscular fitness compared with no intervention/usual care (Cohen’s *d* = 0.18, 95% CI [0.08, 0.28], *p* < .001, 18 studies). There was a significant, moderate effect for lower body neuromuscular fitness outcomes, but no significant effect for upper body outcomes. Only two studies measured changes to resistance training participation, precluding meta-analysis on this outcome. Studies targeted mostly clinical populations and used mobile applications or websites. Majority of studies included bodyweight exercises, prescribed via videos or pictures, along with text description. Exercise prescription was generally poorly reported across studies. Only 7 studies used a theoretical framework to inform their intervention. All studies incorporated BCTs (17 discrete BCTs used), with a focus on providing instruction and demonstrating behavior.

**Conclusions:**

mHealth is a potentially scalable, effective method of prescribing resistance training. Better reporting of exercise prescription, along with clearer grounding in established theoretical frameworks, is recommended.

**Review registration:**

The review was prospectively registered with the International Prospective Register of Systematic Reviews (PROSPERO; registration number CRD42025641142).

**Supplementary Information:**

The online version contains supplementary material available at 10.1186/s12966-025-01868-8.

## Background

Resistance training is independently associated with multiple health outcomes, including reduced risk of all-cause mortality [1, 2], improved prevention and management of diabetes [3, 4], and improved cardiometabolic [5, 6], musculoskeletal [6–9] and mental health [10, 11] outcomes. These health benefits are closely linked to the resultant improvements in muscular strength and endurance and increased lean muscle mass [1]. Despite these well-documented benefits, resistance training has been described as the ‘forgotten’ guideline in the physical activity literature [12]; it is estimated that 32% of adults do not meet the aerobic guidelines, while 70–90% of adults do not meet the resistance training guidelines [13–18]. This represents an important public health challenge and opportunity.

Mobile Health (mHealth) refers to the use of mobile devices and wireless technology to support healthcare provision. It has emerged as a promising platform for the delivery of physical activity interventions, including resistance training [19], due to low maintenance costs and the capacity to reach large portions of the population [20, 21]. mHealth interventions can be delivered through websites, as well as established messaging services (e.g. WhatsApp) and purpose-built mobile applications, often incorporating strategies such as behavior instruction and demonstration (i.e. videos, images), goal setting, self-monitoring, and information provision [22]. Reflecting its potential, meta-analyses of mHealth physical activity interventions have demonstrated small-to-moderate increases in aerobic activity [19]; this likely reflects mHealth technologies being designed to capturing aerobic-oriented metrics (e.g. steps, heart rate, distance, and energy expenditure) and providing immediate feedback and reinforcement suited to steady, repeatable activity patterns (i.e., aerobic activity). It remains unclear whether these benefits extend to resistance training, which poses unique challenges in terms of prescription, technique, and progression. Currently, there has been no synthesis of studies analyzing the efficacy of mHealth interventions that promote resistance training for increasing resistance training behavior or improving neuromuscular fitness. Despite this gap in evidence, the fitness app market is already well-established and growing, with projections estimating growth to USD $55 billion by 2032 [23]. Concurrent with this projected growth, there is a clear need to consolidate existing evidence to inform effective and evidence-based practice.

Specifically, there is a need to quantify the overall efficacy of mHealth interventions that prescribe resistance training in improving neuromuscular fitness and promoting resistance training participation. Additionally, there is a need to describe how resistance training is currently prescribed in mHealth interventions, assess the extent to which these interventions are grounded in behavioral theory and incorporate behavior change techniques. Addressing these gaps is essential to guide the development of scalable, effective mHealth resistance training interventions. As such, the primary objective of this systematic review and meta-analysis was to evaluate the efficacy of mHealth interventions to increase neuromuscular fitness (i.e., strength, strength endurance, power, balance, and submaximal speed) and resistance training participation, including exploring moderators of efficacy. This review also aimed to explore how resistance training is prescribed through mHealth, and the theoretical frameworks and behavior change techniques employed.

## Methods

### Protocol and registration

The Preferred Reporting items for Systematic Reviews and Meta Analyses (PRISMA) protocol was used to guide and report the conduct and reporting of this systematic review (see Supplementary Appendix A) [24]. The review was prospectively registered with the International Prospective Register of Systematic Reviews (PROSPERO; registration number CRD42025641142).

### Eligibility criteria

The population, intervention, comparison, outcomes and study type (PICOS) framework was used to develop the inclusion criteria:

(i) Population: any adult population (aged ≥ 18years).
(ii) Intervention: mHealth interventions including mobile, tablet or web-based apps and websites prescribing resistance training that targets major muscle groups. Other forms of physical activity (e.g., aerobic training) could also be prescribed alongside resistance training. Additional interventions that enhanced the primary mHealth intervention (e.g., activity monitors) could be used. Interventions that included one or more other intervention components (e.g., supervised exercise sessions, regular telephone counselling) were excluded if these interventions were not identical to those delivered in the comparator and therefore their effects could not be separated. eHealth interventions such as telehealth conferencing (via video or phone); text message-based interventions; and interventions centered around the use of a device (e.g., VR headset) were excluded. mHealth interventions that were used only to monitor participation or change behavior, without structured *prescription* of resistance training, were excluded. Interventions that also targeted other lifestyle factors (e.g., diet, sleep) were excluded if the interventions were not identical to those delivered in the comparator and therefore their effects cannot be separated.
(iii) Comparator: no intervention or usual care, or another form of intervention that prescribed resistance training (e.g., in-person supervised sessions).
(iv) Outcomes: interventions had to report at least one outcome of neuromuscular fitness (i.e., strength, strength endurance, power, balance, and submaximal speed) or resistance training participation (e.g. frequency) to be included.
(v) Study type: only randomized controlled trials were included; quasi-randomized and cross-over trials were excluded. Studies published as conference abstracts, dissertations, protocol papers, literature reviews, grey literature, and theses were not considered for this review.

### Search strategy and screening

Seven databases were searched (MEDLINE via OVID, Embase via OVID, Emcare via OVID, SPORTDiscus, Web of Science, Scopus and Cochrane [CINAHL only]) from 1 January 2010 to 1 February 2025, using subject heading, keyword and MeSH term searches for “mHealth”, “cell phone”, “website”, “resistance training”, and “intervention” (see Table S1 for full search strategy). Because mHealth technologies have rapidly evolved, limiting the search to studies published from January 2010 ensured inclusion of research reflecting contemporary mobile device capabilities, app functionality, and digital health practices relevant to current clinical and technological contexts. Reference lists of relevant systematic reviews and trial/study registries were manually searched. Only studies published in English were included.

Search results were uploaded to the reference management software EndNote 20, where duplicates were removed. Search results were then uploaded into Covidence (https://www.covidence.org/home). Additional duplicates were removed in Covidence. Title/abstract and full-text screening were completed in duplicate by two independent reviewers (SB and EC, AJ, ML, NG), with disagreements resolved by discussion until consensus or inclusion of a third reviewer if consensus could not be reached.

### Data extraction

One author (SB) extracted data from the included studies, using a template developed by the authors in Microsoft Excel. A second author (EC, SR) checked the data extraction for accuracy and consistency; disagreements between reviewers were resolved through discussion until agreement was reached, or through the involvement of a third reviewer. The following information was extracted for each study: author name, year of publication, country, trial duration, target population, sample size, age, female (%), study funding source, intervention and control condition details, participant adherence and completion rates, and resistance training participation and neuromuscular fitness outcomes and results (i.e. post-test means and standard deviations). Where a study had a published protocol, this was used to gather additional information not reported in the intervention publication. The behavior change techniques (BCTs) used in the interventions were identified based on the taxonomy of 26 BCTs developed by Abraham and Michie [25]. The theoretical basis of behavior change used and reported BCTs were extracted. BCTs were coded as present or absent, and only BCTs applied in the intervention group/s were extracted.

### Data synthesis

To address objective one, a three-level meta‐analyses was conducted in R using the meta3L package to synthesize Cohen’s *d* across homogenous studies (≥ 5 studies per outcome [26]). This approach partitions variance into: (1) sampling variance within each effect size; (2) covariance among multiple effects from the same study; and (3) variance between studies. Studies included in the meta-analysis compared an mHealth intervention with no intervention or usual care; there was insufficient homogeneity in studies which included another form of intervention that prescribed resistance training as the comparator to conduct a meta-analysis. Further, only two studies reported changes in resistance training participation, so no meta-analysis was conducted on this outcome. Models were fit via restricted maximum likelihood estimation, yielding pooled effects with 95% likelihood‐based CIs.

Effect sizes were converted to Cohen’s *d*, calculated using post-test means and standard deviations for the intervention and control groups, were imported into R and analyzed using the meta3L package. If necessary, standard deviations were estimated from other summary statistics (e.g., standard errors, confidence intervals) using established formulas. A positive *d* indicated a favorable effect of the mHealth intervention relative to the comparator group. Pooled effect sizes were interpreted using conventional thresholds: small (≤ 0.2), medium (0.3–0.7) or large (≥ 0.8). Heterogeneity was quantified using the I² statistic—interpreted as low (0–40%), moderate (30–60%), substantial (50–90%), or considerable (75–100%)—and, where indicated, explored sources of heterogeneity through subgroup analyses and meta-regression [27]. To allow for more precise examination of effect modifiers, separate three‐level meta‐analyses were conducted for lower‐body and upper‐body neuromuscular fitness measures. By stratifying the analyses in this way, homogeneous sets of outcomes were grouped so to explore moderators within each body region‐specific category. Moderator analyses tested key categorical predictors, including intervention length (≤ 12 vs. > 12 weeks), population type (healthy vs. clinical conditions), mHealth delivery mode (app vs. website), dosing strategy (fixed prescription vs. standardized progressive vs. individualized progressive), and – specifically for lower-body neuromuscular fitness measures – outcome subtype (strength vs. strength endurance).

To address objectives two and three, data on study characteristics, population, intervention and comparator groups, and the reported theoretical basis of behavior change and BCTs, were summarized and descriptively tabulated.

### Risk of methodological bias

Risk of bias was assessed using Cochrane RoB2 for randomized controlled trials [28]. RoB2 is structured in five domains: 1) randomization process; 2) deviations from intended interventions; 3) missing outcome data; 4) measurement of the outcome; 5) selection of results, and categorizes answers into “yes”, “probably yes”,” probably no”, “no”, “no information”. Studies were rated as ‘low risk’ of bias if all five domains were judged to be low risk, ‘some concerns’ if at least one domain was judged to have some concern for bias, and ‘high risk’ if at least one domain was judged to be of high risk. Two reviewers (EC, SB) independently assessed included studies for risk of bias. Any disagreements were resolved by discussion until consensus or inclusion of a third reviewer if consensus could not be reached. Where a study had multiple outcomes included in the meta-analysis, a separate risk of bias assessment was completed for each outcome. For studies not included in the meta-analysis, the outcome assessed was a neuromuscular fitness or resistance training participation-related outcome.

### Publication bias

Publication bias was assessed by visually inspecting funnel plots and conducting an Egger’s regression test, which provided a statistical measure of asymmetry in the funnel plot. A *p*-value less than 0.05 indicated evidence of publication bias. As a sensitivity check, Duval and Tweedie’s trim-and‐fill procedure was applied to estimate the number of potentially missing studies and the impact of their imputation on the pooled effect sizes.

## Results

### Search and screening

**Fig. 1:**
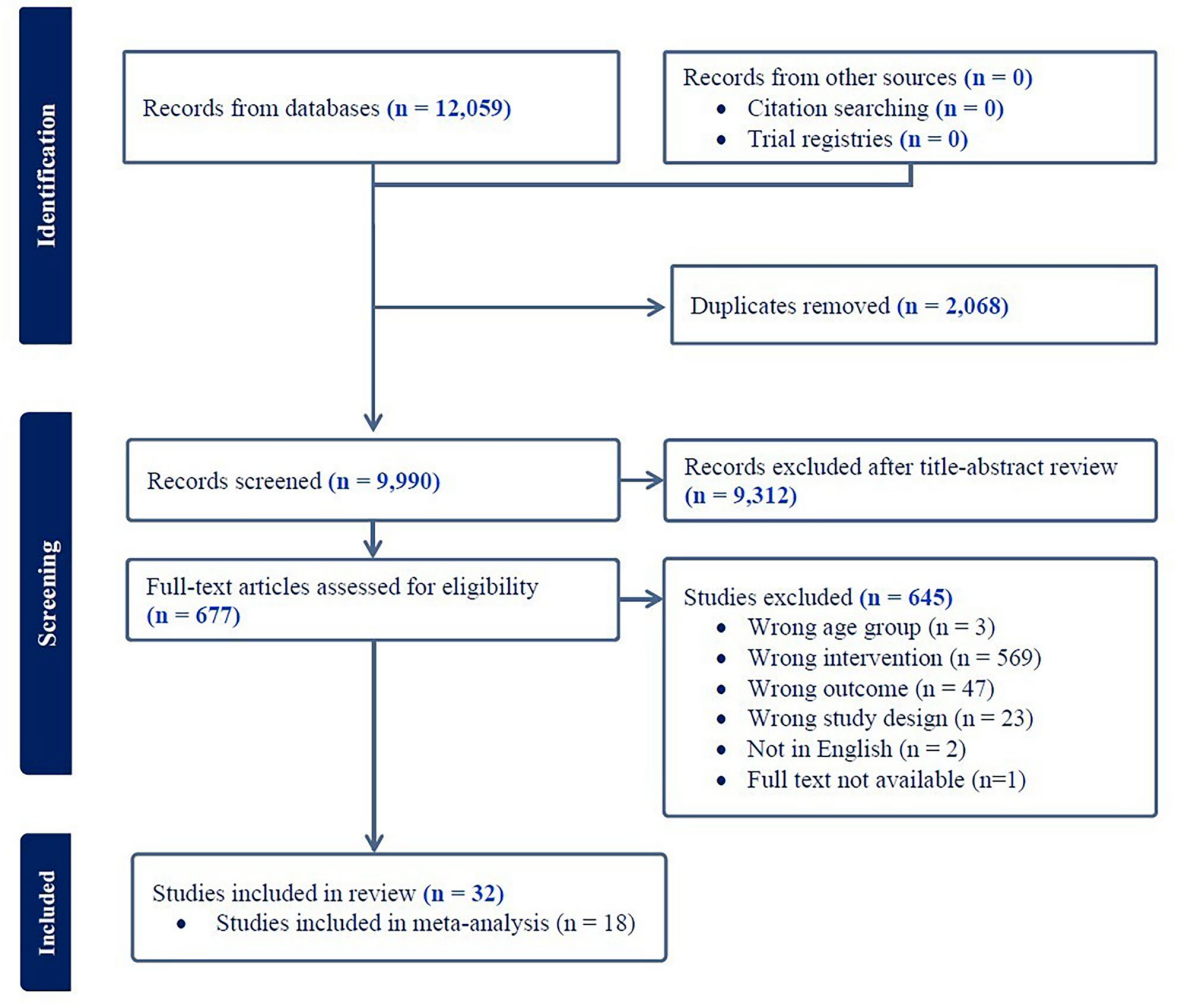
PRISMA diagram. PRISMA, Preferred Reporting Items for Systematic reviews and Meta-Analyses

After a search of the databases, 12,059 records were identified (Fig. 1). Following removal of duplicates, and title and abstract screening, 677 full texts were retrieved; 32 randomized controlled trials were deemed eligible for inclusion in this review. A list of articles excluded at full-text and the reasons for exclusion can be found in Table S2.

### Efficacy of mHealth interventions

**Fig. 2:**
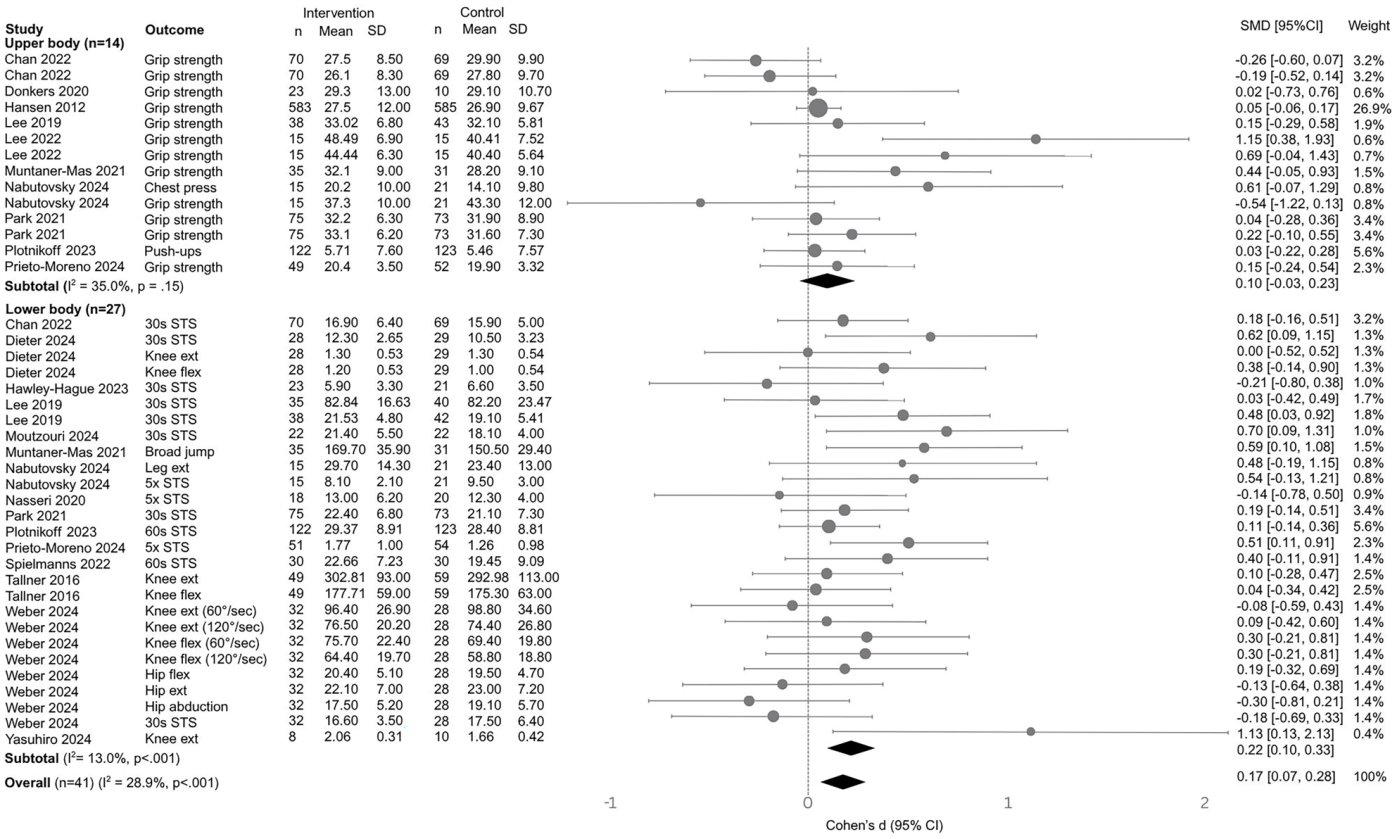
Forest plot of mHealth resistance training by subgroup. CI, confidence interval; hip ext, hip extension; hip flex, hip flexion; knee ext, knee extension; knee flex, knee flexion; leg ext, leg extension; SMD, standardized mean difference; STS, sit-to-stand

Across 18 studies [29–46] (41 effect sizes), mHealth-delivered resistance‐training interventions produced a small, statistically significant improvement in neuromuscular fitness compared with no intervention/usual care (Cohen’s *d* = 0.18, 95% CI [0.08, 0.28], *p* <.001; Fig. 2). When outcomes were stratified by body region, there was a moderate, statistically significant effect for lower body neuromuscular fitness outcomes (15 studies, 27 effect sizes) (*d* = 0.22, 95% CI [0.11, 0.33], *p* <.001; robust SE = 0.06, *p* <.01), while upper body outcomes (10 studies, 14 effect sizes) yielded a small, non‐significant effect (*d* = 0.11, 95% CI [–0.04, 0.26], *p* =.15; robust SE = 0.08, *p* =.19). Heterogeneity was low for the overall model (I^2^ = 28.9%), as well as for upper (I^2^ = 35%) and lower (I^₂^ = 13%) body outcomes.

Moderator analyses were conducted within each of the three meta-analytic models (overall, lower‐body, upper‐body). Only those contrasts that reached statistical significance (*p* <.05) are reported here; the full results are presented in Table S3. In the overall meta‐analysis, dosing approach was analyzed as a categorical moderator by coding “fixed prescription” regimens as the reference group and comparing “standardized progressive” and “individualized progressive” prescriptions against it. Findings showed that fixed prescription yielded the largest mean effect on neuromuscular fitness (*d* = 0.37, 95% CI [0.08, 0.66], *p* =.01), though relative to fixed, standardized progressive dosing (Δ*d* = − 0.27, 95% CI [–0.57, 0.03], *p* =.08) and individualized progressive dosing (Δ*d* = − 0.18, 95% CI [–0.50, 0.15], *p* =.29) were not statistically significantly different. When restricted to lower body neuromuscular fitness outcomes, dosing was a significant moderator: compared with fixed prescription, standardized progressive dosing produced smaller effects (Δ*d* = − 0.28, 95% CI [–0.55, − 0.02], *p* =.04), and individualized progressive dosing did not differ (Δ*d* = − 0.12, 95% CI [–0.42, 0.17], *p* =.41). For neuromuscular fitness outcome sub-type, the included studies only assessed strength and strength endurance; strength and strength endurance measures produced comparable improvements (strength: *d* = 0.21, 95% CI [0.05, 0.37], *p* =.01; strength endurance: *d* = 0.20, 95% CI [0.04, 0.37], *p* =.02; Δ*d* = −0.01, 95% CI [−0.23, 0.20], *p* =.93; R²₃ < 0.01), indicating similar efficacy across these outcome sub-types.

### Participant and trial characteristics

Table 1 outlines the study and sample characteristics of the included studies. A total of 15,045 participants were included across studies, with a weighted mean age of 51.14 ± 13.79 years (ranging from 23 to 77 years) and the majority being females (65%). The populations targeted were musculoskeletal conditions (*n* = 8), healthy adults (*n* = 6), neurological conditions (*n* = 5), cardiopulmonary conditions (*n* = 4), ageing (*n* = 4), people living with or beyond cancer (*n* = 3), and metabolic diseases (*n* = 2). The studies were conducted in Europe (*n* = 18), Asia (*n* = 9), North America (*n* = 6) and Oceania (*n* = 1), and were in community (*n* = 20, 63%), outpatient (*n* = 9, 28%) and inpatient (*n* = 2, 6%) settings.

**Table 1:** Study and sample characteristics

Study	Country, setting	Sample size			Female (%)	Age (years)	Population	Neuromuscular Fitness Outcomes	Funding Source
		Total (*n*)	Intervention (*n*)	Control (*n*)					
Alasfour (2020) [57]	Saudi Arabia, community	40	20	20	100	54.40 ± 4.33	Females with knee osteoarthritis	5x sit-to-stand test	DSR Graduate Students Research Support (GSR)
Chan (2022) [29]	China, outpatient	139	70	69	IG = 37.1 CG = 20.3	59.8 ± 6.6	Adults with coronary heart disease	30 s sit-to-stand test, grip strength	Health and Medical Research Fund (HMRF)of the Food and Health Bureau of the Hong Kong SAR Government (Grant no. 1415 1111)
Dieter (2024) [30]	Germany, community	61	IG1 = 15; IG2 = 15; Total = 30	31	IG = 40; CG = 58; Total = 49	62.9 ± 8.5	Adults with knee osteoarthritis	30 s sit-to-stand test	Sporlastic GmbH
Donkers (2020) [31]	Canada, community	48	32	16	IG = 63 CG = 69	54.3 ± 11.9	People with MS	Grip strength	Hermes Canada | Multiple Sclerosis Society of Canada Wellness Research Innovation Grant; the Saskatoon Health Region; and the College of Medicine, University of Saskatchewan.
Ehling (2017) [47]	Austria, outpatient	20	10	10	IG = 25 CG = 20	NR	People with MS	Motricity index lower extremities	No grant funding
Frevel (2015) [48]	Germany, community	18	9	9	IG = 77.8 CG = 88.9	45.5 ± 7.7	People with MS	Isometric knee strength	NR
Gohir (2021) [49]	UK, community	146	79	67	IG = 70.8 CG = 64.9	67.7 ± 9.2	Adults with knee osteoarthritis	30 s sit-to-stand test, leg torque	Versus Arthritis UK Pain Centre & National Institute for Health Research Nottingham Biomedical Research Centre
Granet (2023) [50]	Canada, community	83	45	38	IG = 83 CG = 81	NR	Older adults aged ≥ 60years	5x sit-to-stand test, 10x sit-to-stand test, 30 s sit-to-stand test, muscle power	Centre de recherche de l’Institut universitaire de gériatrie de Montréal (CRIUGM-Programme appui) & Senior author
Hansen (2012) [32]	Denmark, community	12,287 (1,168 with outcome measures)	6055	6232	IG = 64.8 CG = 64.9	50 ± 13.6	Healthy adults	Grip strength	TrygFonden, Denmark
Hawley-Hague (2023) [33]	UK, community	50	26	24	IG = 65.4 CG = 70.8	77.6	Adults aged ≥ 50years at risk of falls	30 s sit-to-stand test	National Institute for Health and Care Research Applied Research Collaboration-Greater Manchester
Irvine (2013) [60]	USA, community	368	178	190	IG = 71.1 CG = 67.9	60.3 ± 4.9	Adults aged ≥ 50years	Self-report RT time	US National Institutes of Health, National Institute on Aging
Jungreitmayr (2022) [51]	Austria, Italy, community	203	97	106	100	65.3 ± 1.5	Retired females	Grip strength, 30 s sit-to-stand test	Austrian Federal Ministry for Transport, Innovation and Technology grant
Lee (2019) [34]	South Korea, inpatients	96	48	48	0	NR	Men aged > 50years with prostate adenocarcinoma	Grip strength	Ministry of Science, ICT and future Planning (Smart After-Care Service for Cancer and Cardiac Disease)
Lee (2022) [35]	South Korea, community	30	15	15	0	NR	Healthy men aged 40-50years	Grip strength	No external funding
Li (2021) [61]	China, outpatients	101	55	46	IG = 22 CG = 26	48.2 ± 10.4	People with type 2 diabetes	Grip strength	Grant for High-Level Talents from West China Hospital (ZYGD18017), the Science and Technology Department of Sichuan Province (2019YFS0302), and the Recovery Plus Clinic, Chengdu, China.
Li (2022) [58]	China (Hong Kong), outpatients	31	16	15	IG = 93 CG = 69	NR	Adults post-hip fracture	Quadricep muscle strength testing	Caspar Health Ltd
Moutzouri (2024) [36]	Greece, community	50	25	25	IG = 86.4 CG = 68.2	NR	Adults with knee osteoarthritis	30 s sit-to-stand test	European Social Fund
Muntaner-Mas (2021) [37]	Spain, community	66	35	31	IG = 65.7 CG = 83.8	23.1 ± 4	University students	Handgrip strength, standing broad jump, muscular fitness	Institut de Recerca i Innovació Educativa
Nabutovsky (2024) [38]	Israel, outpatients	50	23	27	IG = 21.7 CG = 3.7	59.8 ± 10.4	People undergoing cardiac rehabilitation	Muscle endurance - chest press and leg extension, grip strength, 5x sit-to-stand test	No external funding
Nasseri (2020) [39]	Germany, community	38	18	20	IG = 50 CG = 55	51	People with chronic progressive MS	5x sit-to-stand test	Biogen
Park (2021) [40]	South Korea, outpatients	172	86	86	0	66.4 ± 7.5	Men with prostate cancer undergoing androgen deprivation therapy	30 s sit-to-stand test, grip strength	Ministry of Science, ICT and future Planning
Plotnikoff (2023) [41]	Australia, community	245	122	123	IG = 70.5 CG = 74.0	53.4 ± 13.9	Adults not meeting PA guidelines	90° push-up test, 60 s chair sit-stand	National Health and Medical Research Council of Australia
Prieto-Moreno (2024) [42]	Spain, outpatient	110	55	55	IG = 73 CG = 70 Total = 71		People recovering from hip fracture	Grip strength, 5x sit-to-stand test	EIT Health and the Ramón y Cajal 2021 Excellence Research Grant action from the Spanish Ministry of Science and Innovation.
Rees-Punia (2022) [59]	USA, community	85	45	40	IG = 91.1 CG = 97.5	60.9 ± 7.4	People living beyond cancer	Self-report RT time	The American Cancer Society
Spielmanns (2022) [43]	Germany, Switzerland, inpatients	67	33	34	IG = 48.5 CG = 50.0	64.3 ± 7.7	People with COPD – Global Initiative for Obstructive Lung Disease (GOLD) Stages II–IV	60 s chair sit-stand	Kaia Health software GmbH
Stork (2021) [53]	Canada, community	48	24	24	IG = 50 CG = 50	24 ± 5	Healthy adults aged 18-50years	100-point functional movement screen - squat, in-line lunge, active straight leg raise, trunk stability push up	Mitacs and Lululemon Athletica
Tallner (2016) [44]	Germany, community	126	59	67	IG = 75 CG = 75	40.8 ± 9.9	People with MS	Quadricep extension, Knee flexion/extension, trunk flexion/extension	Hertie Foundation [grant number 1.01.1/09/007]; Bayer Vital GmbH [no grant number]; and the German Foundation for Neurology (Deutsche Stiftung Neurologie) [no grant number]
Tanhan (2024) [54]	Türkiye, outpatients	27	13	14	IG = 38.0 CG = 50.0 Total = 44.4	53.7 ± 10.28	COVID-19 outpatients	30 s sit-to-stand test	Marmara University
Timurtas (2022) [55]	Türkiye, community	90	IG1 = 30; IG2 = 30	30	NR	NR	People aged 30-65years with type 2 diabetes	Grip strength, knee flexion strength	Scientific and Technological Research Council of Turkey (TUBITAK)
Weber 2024 [45]	Germany, community	60	32	28	IG = 66 CG = 57	62 ± 7	Adults with knee and hip osteoarthritis	30 s sit-to-stand test, isometric strength (hip and knee)	Projekt DEAL
Yasuhiro (2024) [46]	Japan, community	21	11	10	IG = 100 CG = 90 Total = 94	NR	Healthy hospital staff	Grip strength, knee extension strength	NR
Ziebart (2024) [56]	Canada, outpatients	69	34	35	89	60 ± 3.4	Adults with distal radius fracture	Grip strength, 5x sit-to-stand test	CIHR Foundation Grant [167284]

Data presented as mean or mean ± standard deviation

*CG *control group, *COPD* chronic obstructive pulmonary disease, *IG* intervention group, *MS* multiple sclerosis,* NR* not reported., *PA *physical activity, *RT* resistance training

### Resistance training prescription in mHealth interventions

Table 2 shows the mHealth intervention characteristics. These were delivered by mobile applications (*n* = 22, 69%) and websites (*n* = 10, 31%). Resistance exercises were mostly prescribed using videos (*n* = 25, 78%) featuring real people (*n* = 15) or animations (*n* = 4); six studies did not report video format. Nine studies (28%) used pictures to prescribe resistance exercises, featuring real people (*n* = 6) or animations (*n* = 2); one study did not report image format. Over half of the studies (*n* = 16) reported using text descriptions of exercises. 

**Table 2:** Intervention details

Study	mHealth mode	mHealth name	Mode of RT instruction		RT prescription	Intervention duration	Adherence (%)		Completion (%)		Comparator
			Format (real or animated)	Text descriptions			Intervention	Comparator	Intervention	Comparator	
Alasfour (2020) [57]	App	My Dear Knee	Videos (real)	Y	**F**: Daily **I**: NR **T**: NR **Ty**: BW, band **V**: 1 set x 10 reps, 10 s rest, 2–9 exercises **P**: Progressive (standardized)	6 weeks	85.35 ± 13.73	60.19 ± 33.10	90	85	Other intervention – paper-based exercise intervention
Chan (2022) [29]	App	ZTExApp	Pictures (real)	Y	**F**: NR **I**: NR **T**: NR **Ty**: BW **V**: NR **P**: NR	12 weeks	NR	-	80	82	Control – education
Dieter (2024) [30]	App	re.flex	Videos (real, animated)	Y	**F**: 3x/week **I**: NR **T**: 25–30 min **Ty**: BW, band, household items, specialized knee brace with sensors **V**: 5 exercises **P**: Progressive (individualized) – user	12 weeks	92.5	-	90	97	Control – usual care
Donkers (2020) [31]	Website	webbasedphysio.com	Videos (real)	Y	**F**: ≥2x/week **I**: Individualised **T**: NR **Ty**: NR **V**: Individualised **P**: Progressive (individualized) – researcher	26 weeks	All = 74.9 Walkers = 65.3 Wheelchair users = 99.2	All = 66.5 Walkers = 65.3 Wheelchair users = 69.2	81.3	68.8	Control – exercise advice
Ehling (2017) [47]	App	MS-spasticity APP	Videos (real)	NR	**F**: Daily **I**: NR **T**: 2 × 15 min **Ty**: BW, household items **V**: NR **P**: NR	13 weeks	80	NR	90	90	Other intervention – paper-based exercise intervention
Frevel (2015) [48]	Website	NR	Pictures (NR)	Y	**F**: 2x/week **I**: RPE 11–14/20 **T**: 45 min **Ty**: BW, band, mat, gym ball, household items **V**: 2–3 sets x 8–15 reps, 5–8 exercises **P**: Progressive (individualized) – algorithm	12 weeks	NR	NR	88.9	88.9	Other intervention – hippotherapy
Gohir (2021) [49]	App	iBEAT-OA	NR	NR	**F**: Daily **I**: NR **T**: 20–30 min **Ty**: NR **V**: NR **P**: Progressive (individualized) – researcher	6 weeks	87.9 ± 14.3	-	71.6	72.2	Control – usual care
Granet (2023) [50]	Website	‘Training recommend’	Videos (real)	NR	**F**: 3x/week **I**: NR **T**: 55 min **Ty**: BW, weights **V**: NR **P**: Progressive (standardized)	12 weeks	81	89	54	84	Other intervention – eHealth (Zoom exercise intervention)
Hansen (2012) [32]	Website	NR	NR	NR	Individualised based on baseline physical activity levels **Ty**: NR **P**: NR	6 months	7	-	59	67	Control – usual care
Hawley-Hague (2023) [33]	App	‘Motivate Me’, ‘My Activity Programme’	Pictures (animated)	NR	Individualised based on goals **Ty**: NR **P**: NR	6 months	NR	-	73	79	Control – exercise advice
Irvine (2013) [60]	Website + CD rom	Active After 55	Pictures (real), Videos (animated)	Y	Individualised based on participant self-assessed ability **Ty**: Individualised, (minimal equipment) **P**: Progressive (standardized)	12 weeks	NR	-	70.2	94.7	Control – usual care
Jungreitmayr (2022) [51]	App	NR	NR	NR	**F**: 2-3x/week **I**: RPE 5–7/10 or 15–18/20, **T**: 10–30 min **Ty**: BW, band, ball, household items **V**: 2 set x 8–12 reps or 40 s, 1–3 min rest, 3–10 exercises **P**: Fixed	14 weeks	Frequent users (*n* = 13) = 233.2 ± 66.8; Occasional users (*n* = 9) = 147.5 ± 54.6; Rare users (*n* = 15) = 83.9 ± 23.9; Non-users (*n* = 15) = 31.4 ± 20.6	-	56.7	78.3	Control – usual care
Lee (2019) [34]	App	Smart After-Care	Videos (real)	NR	**F**: 2x/week **I**: NR **T**: NR **Ty**: BW, band **V**: 2 sets x 10 reps **P**: Progressive (standardized)	12 weeks	92.5	79.5	76.0	86.0	Control – exercise advice, pedometer
Lee (2022) [35]	Online platform	NR	Videos (NR)	NR	**F**: NR **I**: 60% HRR **T**: 45 min **Ty**: BW **V**: 3 sets x 12 reps, 60 s rest **P**: Fixed	20 weeks	89	NR	100	100	NR
Li (2021) [61]	App	R Plus Health	Videos (real)	NR	**F**: NR **I**: NR **T**: NR **Ty**: NR **V**: NR **P**: Progressive (individualized) – algorithm	12 weeks	NR	-	80.0	89.1	Control – exercise advice
Li (2022) [58]	App	Caspar Healthe-system	Pictures (real), Videos (real)	NR	**F**: 2x/week **I**: Individualised **T**: Individualised **Ty**: BW, household items **V**: Individualised **P**: Progressive (individualized) – researcher	3 weeks	87	86	100	94.0	Other intervention – paper-based exercise intervention
Moutzouri (2024) [36]	Website	West Walks	Videos (real)	NR	**F**: 2x week **I**: NR **T**: NR **Ty**: NR **V**: NR **P**: NR	6 weeks	70	48	88	88	Control – exercise advice
Muntaner-Mas (2021) [37]	App	vidahora	Videos (animated)	Y	**F**: 3x/week **I**: NR **T**: >10 min **Ty**: NR **V**: NR **P**: Fixed	9 weeks	95	-	70	62	Control – usual care
Nabutovsky (2024) [38]	App	RCR-ST	Videos (NR)	NR	**F**: 2x/week **I**: NR **T**: NR **Ty**: BW, band, weights, household items **V**: 8 exercises **P**: Progressive (individualized) – researcher	16 weeks	100 ± 110	30 ± 35	65.2	77.8	Control – exercise advice, education
Nasseri (2020) [39]	App	NR	Pictures (animated), Videos (NR)	Y	NR	12 weeks	NR	-	100	90	Control – education
Park (2021) [40]	App	Smart After-Care	Videos (NR)	Y	**F**: 2x/week **I**: NR **T**: NR **Ty**: NR **V**: 2 sets x 10 reps **P**: Fixed	12 weeks	NR	-	87.2	84.9	Control – exercise advice
Plotnikoff (2023) [41]	App	ecofit	Videos (animated)	Y	**F**: 2x/week **I**: Individualised based on participant self-assessed ability **T**: 20–55 min **Ty**: BW, outdoor gyms **V**: 2 sets x 8 reps, 8 exercises **P**: Progressive (individualized) – user	13 weeks	NR	-	65	68	Control – usual care
Prieto-Moreno (2024) [42]	App	ActiveHip +	Videos (real)	NR	**F**: 2x/week **I**: RPE 3–8/10 **T**: 30–60 min **Ty**: BW, household items **V**: 60 s rest, 8–10 exercises **P**: Progressive (individualized) – researcher	13 weeks	NR	-	92.7	98.2	Control – exercise advice, education
Rees-Punia (2022) [59]	App	Healed	Videos (NR)	NR	**F**: NR **I**: NR **T**: NR **Ty**: BW **V**: NR **P**: Fixed	12 weeks	NR	-	77.8	80.0	Control – usual care
Spielmanns (2022) [43]	App	Kaia COPD	Pictures (real), Videos (real)	Y	Individualised based on baseline exercise capacity **Ty**: BW **P**: Progressive (individualized) – algorithm	26 weeks	36	-	90.9	88.2	Control – usual care
Stork (2021) [53]	App	Movr	Pictures (real), Videos (real)	Y	**F**: 4x ‘mini’ and 2x ‘builder’/week **I**: NR **T**: ‘Mini’ – 5 min, ‘builder’ – 15–50 min **Ty**: BW, band, weights (based on availability of equipment) **V**: NR **P**: Progressive (individualized) – user	8 weeks	NR	-	96	96	Control – usual care
Tallner (2016) [44]	Website	Motion Net e-Training	Pictures (real)	Y	**F**: 2x/week **I**: RPE 11–16/20 **T**: NR **Ty**: BW, band **V**: 2–3 sets x 6–20 reps, 1–2 min rest **P**: Progressive (individualized) – user	13 weeks	73	-	83.1	88.1	Control – usual care
Tanhan (2024) [54]	App	FizyoTr	NR	NR	**F**: 3x/week **I**: NR **T**: 30–40 min **Ty**: NR **V**: NR **P**: Progressive (individualized) – researcher	8 weeks	92	84	92.8	93.3	Other intervention – eHealth (Zoom exercise intervention)
Timurtas (2022) [55]	App	DIABETEX	Videos (real)	Y	**F**: 3x/week **I**: NR **T**: 30–60 min **Ty**: NR **V**: NR **P**: Progressive (individualized) – researcher	12 weeks	NR	NR	IG1 (app-only) = 83.3; IG2 (app + smartwatch) = 80.0	86.7	Other intervention – supervised, in-person exercise intervention
Weber 2024 [45]	App	Join2Move	Videos (real)	Y	**F**: 3x/week **I**: NR **T**: NR **Ty**: BW, band, household items **V**: Reps and sets individualized, 2–3 exercises **P**: Progressive (standardized)	12 weeks	NR	-	81	86	Control – usual care
Yasuhiro (2024) [46]	Website/ App	YouTube	Videos (real)	NR	**F**: ≥3x/week **I**: NR **T**: NR **Ty**: BW **V**: 3 sets x 10 reps, 12 s rest **P**: Fixed	12 weeks	NR	-	72.7	100	Control – usual care
Ziebart (2024) [56]	Website	handsup-program.com	Videos (real)	NR	**F**: 2x/week **I**: NR **T**: 45 min **Ty**: BW, band **V**: NR **P**: Progressive (individualized) – user	6 weeks	55	-	61.8	51.4	Control – usual care

Data presented as mean or mean ± standard deviation. BW, bodyweight. CG, control group. FITT-VP: F, frequency; I, intensity; T, time (session); Ty, type (modality) of resistance training; V, volume (sets, reps, rest, number of exercises); P, progression. HRR, heart rate reserve. IG, intervention group. NR, not reported. Reps, repetitions. RPE, rating of perceived exertion. RT, resistance training.

Progression of training dose categorised as (1) ‘progressive (individualized) – researcher’ indicating the dose was progressed individually for participants, by a researcher/clinician, (2) ‘progressive (individualized) – user’ indicating the dose was progressed individually for participants, by the user, (3) ‘progressive (individualized) – algorithm’ indicating the dose was progressed individually for participants, by a platform algorithm, (4) ‘progressive (standardized)’ indicating the dose was progressed in a standardized way for all participants, or (5) fixed dose with no progression.

 Intervention duration ranged from three weeks to six months and was most frequently 12 weeks (*n* = 12, 38%). The prescribed dosage of resistance training varied between studies. The prescribed frequency of sessions was reported in 22 studies (69%) [30, 31, 34, 47–51] and ranged from 1 to 7 sessions per week, with most studies (*n* = 17) prescribing 2–3 sessions per week. The prescribed intensity of the sessions was reported in five studies (16%) [35, 42, 44, 48, 51], with all prescribing moderate-to-vigorous intensity. Intensity was typically prescribed using the Borg 6–20 (*n* = 3) or Borg CR10 (*n* = 1) rating of perceived exertion scales [52]. Session time was reported in 14 studies (44%) [30, 35, 37, 41, 42, 47–51, 53–56] and ranged from 10 to 60 min; sessions were most often prescribed for 30–60 min (*n* = 11). The type (modality) of resistance training was reported in 19 studies (59%); exercises were prescribed using bodyweight (*n* = 19) [29, 30, 34, 35, 38, 41, 43–48, 50, 51, 53, 56–59], resistance bands (*n* = 10) [30, 34, 38, 44, 45, 48, 51, 53, 56, 57] and/or weights such as dumbbells and barbells (*n* = 3) [38, 50, 53]. Other equipment used included household items such as chairs and tables (*n* = 6) [38, 42, 47, 48, 51, 58], gym balls (*n* = 3) [44, 48, 51], outdoor gym equipment (*n* = 1) [41] and a specialized knee brace with sensors (*n* = 1) [30]. Equipment (e.g., bands, gym ball, specialized knee brace with sensors) was provided to participants in six studies [30, 34, 48, 51, 56, 57]. Only two (6%) studies provided complete information on the volume of resistance training prescribed, including sets, repetitions, number of exercises and rest periods [51, 57]; eleven (34%) studies provided no information on volume. Nine studies (28%) reported the prescribed sets and/or repetitions for the resistance exercises [34, 35, 40, 41, 44, 46, 48, 51, 57]. Majority of these studies (*n* = 8) prescribed two to three sets. The prescribed repetitions for each set varied between the studies, ranging from 6 to 20; most (*n* = 6) were in the 8–12 repetition range. The rest period between sets and/or exercises was reported by six studies (19%) [35, 42, 44, 46, 51, 57]; this ranged from 10 s to three minutes, with one minute most common (*n* = 3). Eight studies (25%) [30, 38, 41, 42, 46, 48, 51, 57] reported the number of exercises prescribed per session; this ranged from 2 to 10, with 5–8 exercises most used (*n* = 4). Eight studies (25%) individualized one of more components of the resistance training prescription [31–33, 41, 43, 45, 58, 60]; this was typically based on the participants’ physical activity levels [32], goals [33], exercise capacity [43], or researcher/clinician [31] or participant self-assessed ability [41, 60], at baseline. To manage resistance training dose (exercise volume and type), most studies (*n* = 20, 63%) used a **progressive** approach; this was achieved via individualized progressions made by a researcher/clinician (*n* = 7) [31, 38, 42, 49, 54, 55, 58], the user (*n* = 5) [30, 41, 44, 53, 56] or platform-based algorithm (*n* = 3) [43, 48, 61], or using a standardized approach for all participants (*n* = 5) [34, 45, 50, 57, 60]. Six studies (19%) prescribed a fixed dose of exercise with no progression over the intervention [35, 37, 40, 46, 51, 59]. Six studies (19%) did not report whether the dose was progressed during the intervention [29, 32, 33, 36, 39, 47].

Eighteen studies (56%) reported adherence to the prescribed intervention [30–32, 34–38, 43, 44, 47, 49–51, 54, 56, 57, 61]; the mean adherence was 84.4% (range 7–100%). The definition of adherence varied among the studies but was predominately based on the number of sessions completed as a proportion of the number prescribed (*n* = 9) [30, 31, 35–38, 49, 50, 54] or mHealth platform usage (*n* = 3) [32, 43, 60]. Adherence was determined from mHealth platform usage (*n* = 9) [30, 32, 37, 43, 44, 47, 49, 51, 61], participant self-report (e.g., via exercise diaries; *n* = 7) [31, 34–36, 38, 57, 58] and attendance at sessions (*n* = 2) [50, 54]. In studies that compared an mHealth intervention with another intervention that prescribed resistance training (e.g., eHealth), and reported adherence in both groups (*n* = 4) [50, 54, 57, 58], the mean adherence rate was 86% (range 81–92%) versus 80% (range 60–89%) in the comparator. All 32 studies reported completion rates; the mean completion for the intervention was 80% (range 54–100%), compared with 84% (range 51–100%) in the comparator.

### Behavior change techniques used in mHealth interventions

Seven (22%) of the studies reported using a behavior change theoretical framework to inform their mHealth intervention; these included Theory of Planned Behavior (*n* = 3, 43%) [32, 33, 60], Bandura’s Social Cognitive Theory (*n* = 2, 29%) [41, 59], Transtheoretical Model (*n* = 1, 14%) [55], and Health Action Process Approach Model (*n* = 1, 14%) [29].

All studies incorporated BCTs into their mHealth intervention, with 17 discrete BCTs used across the 32 studies (Table S4). The mean number of BCTs used in an intervention was five (range 2–10). “Provide instruction” (94%), “model/demonstrate the behavior” (81%), “set graded tasks” (72%), “provide information on consequences” (41%) and “prompt practice” (34%) were the most used.

### Risk of methodological bias

**Fig. 3:**
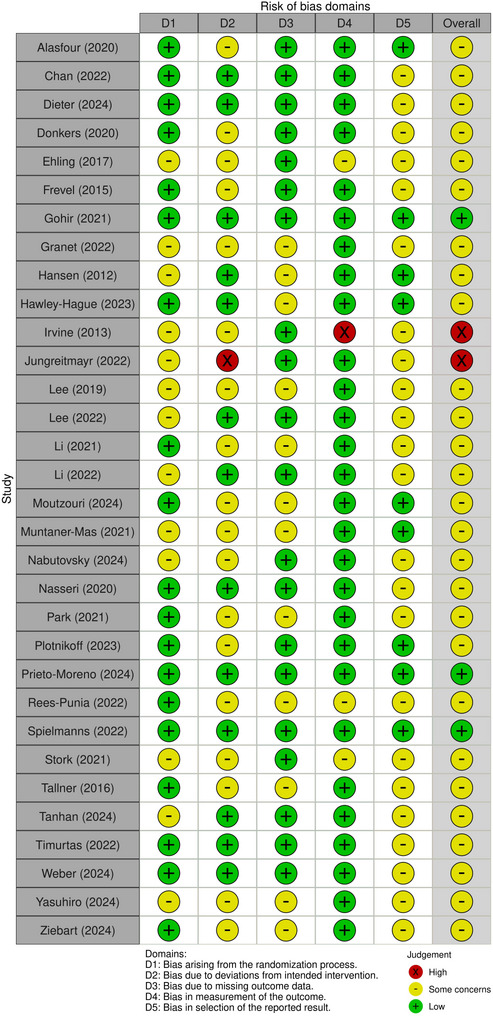
Risk of Bias for all studies included in this review. To be printed in color

Studies were classified as low (*n* = 3), some concerns (*n* = 27) or high (*n* = 2) risk of bias (Fig. 3). The most common sources of bias were deviations from the intended interventions (*n* = 19), often due to the use of per-protocol analyses, and bias in the selection of reported results (*n* = 23), mainly due to poor availability of pre-published statistical analysis plans. Most studies (*n* = 21) were judged to have bias across multiple domains. The 41 outcomes assessed in the meta-analysis were classified as low (*n* = 3) and some concerns (*n* = 38) (Figure S1). ‘Measurement of the outcome’ was rated as having low risk of bias for all outcomes.

### Publication bias

Small-study effects were evaluated using funnel‐plot inspection and Egger’s regression (Figure S2). For the overall analysis (*k* = 18, *n* = 41), Egger’s test indicated significant asymmetry (*z* = 2.66, *p* =.0078), consistent with the possibility that smaller studies may report larger effects. As a sensitivity check, Duval and Tweedie’s trim‐and‐fill was applied, which imputed three potentially missing studies and yielded an adjusted pooled estimate of d = 0.14 (95% CI [0.04, 0.24]). Because this “corrected” effect size is substantively similar to the unadjusted estimate (d = 0.18, 95% CI [0.08, 0.28]) and leads to the same practical interpretation - a small, positive effect - the unadjusted results are presented as the primary findings, while acknowledging this limitation. This approach treats trim‐and‐fill as a sensitivity analysis rather than a definitive correction and ensures clarity and consistency in reporting.

## Discussion

This review sought to evaluate the efficacy of mHealth interventions that prescribe resistance training for improving neuromuscular fitness and resistance training participation. This review also explored how resistance training is prescribed through mHealth-based interventions, and the theoretical frameworks and BCTs employed. By synthesizing findings across a diverse range of studies, this review provides insight into the efficacy of mHealth interventions and how they can be utilized at a larger scale to reach large proportions of the population, both in public and clinical settings.

A meta-analysis was conducted to assess the short-term efficacy of mHealth interventions on neuromuscular fitness. There was a small but statistically significant improvement in neuromuscular fitness following mHealth interventions, compared with no intervention or usual care. While modest, improvements in neuromuscular fitness contribute to improved cardiometabolic health, physical functioning and quality of life among general and clinical populations [5, 62, 63]. Though this review was unable to conduct a direct comparison of mHealth and in-person interventions, the effects seen in this review appear smaller than those observed in a recent review of in-person interventions [64]. However, the scalability and accessibility of mHealth interventions can ensure that even small effects can be meaningful at the population level. The low heterogeneity across models indicates consistency in effect sizes across studies, despite the variability in intervention design and dose, strengthening the reliability of the findings. The studies were generally short in length, which is consistent with previous mHealth intervention reviews [65]. While these outcomes demonstrate positive short-term effects, there is a need for longer-term studies to assess the sustainability of this area of research.

There was a significant, moderate effect for lower body neuromuscular fitness outcomes, but no significant effect for upper body neuromuscular fitness outcomes. The studies in this review predominantly included bodyweight exercises, with weighted exercises typically not used. In these scenarios, available options for bodyweight-only upper-body exercises are limited; while exercises targeting the chest and arms, such as push-ups and triceps dips, can easily be prescribed and progressed, exercises targeting other key muscle groups (i.e., back and shoulders) are more difficult to prescribe and progress without equipment. In contrast, lower body resistance exercises can be effectively adapted and loaded using bodyweight, including for both trained and untrained populations. Further, multiple studies targeted clinical populations; given the established exercise guidelines for conditions such as knee osteoarthritis [66], post-hip fracture [67] and falls prevention [68] emphasize lower-body resistance training, it is likely that the exercise prescriptions within these interventions were focused on the lower body. These factors likely contributed to significant changes in lower body, rather than upper body, neuromuscular fitness.

Only two studies included in this review measured changes in resistance training participation and thus could not be meta-analyzed. As a result, conclusions cannot be made regarding short- or long-term changes to resistance training participation following mHealth interventions. Previous reviews assessing mHealth interventions have found small- to moderate increases in physical activity behavior in the short-and long term [19, 69]. These reviews assessed changes in device-measured aerobic physical activity (i.e., daily step count, accelerometry). Unlike aerobic physical activity, validated and reliable methods of measuring resistance training behavior do not currently exist [70], thus researchers are reliant on self-report measures. Development and validation of a gold-standard measure to assess the FITT-VP of resistance training is needed [70]. Positively, over half of the studies measured adherence to the resistance training prescription (via the number of sessions completed as a proportion of the number prescribed), with studies reporting generally good adherence (average adherence 84.4% across studies). However, the definitions and methods of measuring adherence were inconsistent.

An unexpected finding from the meta-analysis was the moderating effect of dosing, whereby interventions implementing fixed prescriptions were associated with significant outcomes, while those utilizing progressive dosing, whether standardized or individualized, did not yield statistically significant effects. One potential explanation for this is that fixed prescriptions may be simpler to follow, making them more appealing and achievable for participants, especially those new to resistance training. Therefore, fixed dosing protocols may encourage greater consistency and adherence, which are key to improvements in neuromuscular fitness [71]. In contrast, progressive dosing, while theoretically more effective, may not translate into superior outcomes if progression is not adequately implemented, individualized, or adhered to. Without regular supervision or input from exercise professionals, progression may have been ill-suited to participants, with prescribed exercises being above or below participant capabilities. In addition, regularly changing or modifying the exercises without supervision may have negatively impacted the confidence and self-efficacy of participants, potentially reducing their exercise adherence. A further consideration is the limited individualization mechanisms observed across included mHealth interventions. Unlike aerobic-based mHealth programs, which often adapt goals and feedback based on users’ performance data, most resistance training interventions relied on static prescriptions with little or no real-time feedback. The absence of responsive, individualized progression likely constrained participants’ ability to adjust load or intensity appropriately, limiting training stimulus and engagement. Future mHealth resistance training interventions should aim to integrate real-time biofeedback and adaptive algorithms to tailor exercise prescription and progression according to user performance, supporting both scalability and individualization. It is also important to note that standardized dosing protocols were often poorly reported across studies, raising the possibility that some form of progression, such as participant-guided adjustments, may have been implemented. Additionally, only a small number of studies employed a fixed dosing prescription, limiting the generalizability of the finding. To promote scalability of mHealth, future studies may benefit from designing exercise prescriptions that allow for continuity of exercises in the short-term but also encourage and enable participants to progress as they feel confident in the longer-term.

This review also described how resistance training is prescribed via mHealth-based interventions. Overall, the mHealth interventions were prescribed to a range of populations, most commonly using mobile apps with a focus on visual media, which is positive for scalability. The studies mostly targeted clinical populations, with only six studies targeting healthy adults. As such, mHealth may act as a valuable supplemental tool for clinicians seeking to prescribe resistance training to individuals under their care. mHealth may be particularly useful in contexts where traditional exercise services are limited by barriers such as geographic isolation and financial constraints. This is salient for scalability given apps are accessible and usable among most populations due to the high and increasing prevalence of mobile phone and tablet use. Studies mostly prescribed bodyweight exercises, with instruction typically delivered through visual formats such as videos or images. Visual and multimedia are useful for breaking down barriers related to language proficiency and literacy by providing accessible, easy-to-follow instruction. This plays a key role in developing user comprehension, as quality visual demonstrations can promote proper exercise technique which is key to avoiding injury and improving neuromuscular fitness [72]. Such features, along with the accessibility of bodyweight exercises, further contribute to the scalability of mHealth interventions prescribing resistance training.

Reporting of exercise prescription (frequency, intensity, time, type, volume, progression [FITT-VP]) was generally poor across the studies. For example, only 16% of studies reported exercise intensity, which is a major gap in reporting, and possibly in intervention design. To improve replicability and in turn scalability [73], better reporting of prescription is recommended. In those that did report exercise dose, there was large variability, though this may be a result of the different populations (i.e. healthy, clinical) included, given resistance training guidelines and recommendations differ based on the presence of conditions [74–76]. Positively, 2–3 sets of 8–12 repetitions were regularly prescribed, consistent with resistance training guidelines for the general, and multiple clinical, populations [66, 67, 77, 78]. Considering the positive findings of the meta-analysis, using mHealth to prescribe resistance training according to recommended guidelines appears to be a low-cost, manageable and effective means of RT delivery.

Theoretical frameworks were reported in only 22% of studies included in this review, suggesting that most interventions lacked a theoretically grounded foundation. This is common in mHealth research and represents a significant limitation given theory-informed interventions are generally more effective in promoting sustained behavior change [79–81]. There is a need for greater integration of theory in intervention design to enhance rigor and efficacy. The theoretical frameworks that were employed were diverse; this may reflect a lack of consensus on the most appropriate theoretical model for mHealth-delivered resistance training. Future studies should explicitly report the theoretical basis and rationale for selected frameworks. Despite most studies not being explicitly grounded in theory, a large variety BCTs were used. The average of five BCTs per intervention indicates moderate complexity, which likely balances enhanced behavior change and thus intervention efficacy with a non-significant user burden, suggesting a pragmatic approach to behavior change. Previous reviews assessing the promotion of physical activity through mHealth interventions (focusing on aerobic activity) have identified goal setting, self-monitoring, and social support as the most frequently employed BCTs [82, 83], reflecting an emphasis on enhancing motivation to engage in physical activity. The mHealth interventions included in this review placed more emphasis on providing instruction, demonstrating behavior and setting graded tasks, indicating a focus on improving knowledge and skills to complete resistance training. This is appropriate, given these techniques address commonly cited barriers to participating in resistance training such as the lack of skills (i.e., executing each exercise correctly and safely) and knowledge of what exercises to include, and low self-efficacy [84]. In addition to being highly relevant to resistance training, these BCTs are well-suited to mHealth delivery, as digital platforms can effectively provide instructional content, deliver demonstrations through multimedia, and support graded progression with personalized feedback and tracking features.

### Strengths and limitations

A strength of this review is its novelty; this is the first review to assess the pooled effect of mHealth interventions prescribing resistance training on neuromuscular fitness. It also fills an important gap in the literature by describing how resistance training is prescribed using mHealth, a growing field of research and practice. This review has been conducted with high methodological rigor, pre-registering with PRSOPERO, adhering to PRISMA guidelines and completing a meta-analysis of efficacy.

A limitation of the study is the inclusion papers only published in English, which may have resulted in eligible studies being omitted from the review. Another limitation is the short duration of interventions included in this review, limiting the long-term generalizability of these findings. Risk of methodological bias assessments raised some concerns over the quality of statistical analyses among studies included in this review; therefore, results should be interpreted with some caution. Finally, BCTs were only coded as present or absent; this approach was necessary due to inconsistent reporting of intervention detail across studies, which limited the ability to assess BCT dose or intensity and their potential influence on intervention effectiveness.

## Conclusions

This systematic review and meta-analysis found small to moderate effects of mHealth interventions that prescribe resistance training on neuromuscular fitness. Reflecting this, mHealth presents a potentially scalable, effective method to prescribe resistance training to both clinical and healthy adult populations. By leveraging widely accessible mobile technologies, mHealth interventions can overcome common barriers to resistance training participation and reach large proportions of the population. As such, mHealth may act as a valuable supplemental tool for clinicians seeking to prescribe resistance training to individuals under their care. However, current interventions often lack clear, comprehensive exercise prescription, and are rarely grounded in behavioral theory, limiting their potential impact. To advance the field, future research should focus on improving the consistency and transparency of prescription reporting (including all FITT-VP parameters), integrating appropriate behavioral theories, and evaluating the long-term sustainability of effects.

## Supplementary Information

Supplementary Material 1.

Supplementary Material 2.

Supplementary Material 3.

Supplementary Material 4.

Supplementary Material 5.

Supplementary Material 6.

Supplementary Material 7.

## Data Availability

All data relevant to the study are included in the article or uploaded as supplementary information.

## Declarations

Acknowledgements.
