# Supplementary figures and images for: Effects of mHealth interventions to prescribe resistance training: a systematic review and meta-analysis of randomized controlled trials
Source: Int J Behav Nutr Phys Act. 2025 Dec 22;23:7. doi: 10.1186/s12966-025-01868-8 (PMC12836956; doi:10.1186/s12966-025-01868-8)

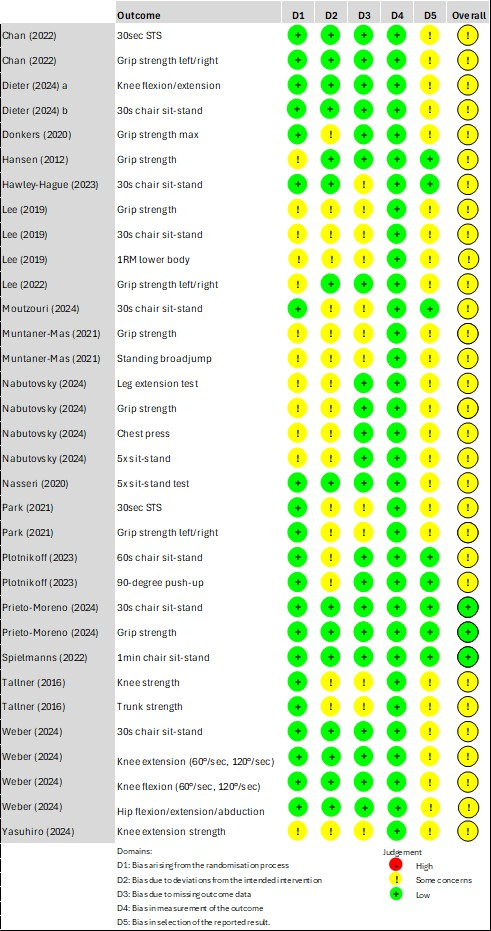

Supplement: Supplementary file 1 — Supplementary Material 1. [file 12966_2025_1868_MOESM1_ESM.tif]

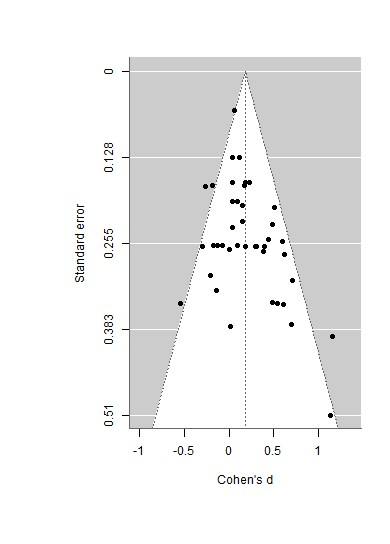

Supplement: Supplementary file 2 — Supplementary Material 2. [file 12966_2025_1868_MOESM2_ESM.tif]
