# Supplementary material for: Effects of mHealth interventions to prescribe resistance training: a systematic review and meta-analysis of randomized controlled trials
Source: Int J Behav Nutr Phys Act. 2025 Dec 22;23:7. doi: 10.1186/s12966-025-01868-8 (PMC12836956; doi:10.1186/s12966-025-01868-8)
Supplement: Supplementary file 4 — Supplementary Material 4. [file 12966_2025_1868_MOESM4_ESM.docx]

**Effects of mHealth Interventions to Prescribe Resistance Training: A Systematic Review and Meta-Analysis of Randomized Controlled Trials**

**Sports Medicine**

Emily R. Cox, Sam Beacroft, Anna K. Jansson, Levi Wade, Mitch J. Duncan, David R. Lubans, Sara L. Robards, Manuel Leitner, Niklas Gutberlet, Ronald C. Plotnikoff^*^

Ron Plotnikoff, [ron.plotnikoff@newcastle.edu.au](mailto:ron.plotnikoff@newcastle.edu.au)

ATC314*,* University Drive*,* Callaghan, NSW 2308*,* Australia

Tel: +61 (02) 49854465

Fax: 61 + 2 49212084

**TABLE S1. Search strategy**

| **Database** | **Search Strategy** |
| --- | --- |
| **Cochrane library** | (“cell phones” OR “cell*phone*” OR cell-phone OR “mobile phone*” OR mobile-phone OR “mobile device” OR “mobile telephone*” OR i*Phone* OR android OR iOS OR “mobile health” OR mHealth OR m-health OR app OR apps OR smartphone NEXT/2 (application OR app) OR mobile NEXT/2 (application* OR app) OR “information technology intervention” OR “IT*intervention” OR eHealth OR e-health OR website OR web-based OR “web based” OR internet-based OR “internet based” OR internet OR tablet-based OR “tablet based”) AND  (resistance NEXT/1 (training OR fitness) OR muscle NEXT/1 (strength* OR fitness OR building) OR muscular OR strength OR strength-balance OR physical* NEXT/1 (fit* OR train* OR activit*) OR train* NEXT/1 (strength* or exercise* or fitness)) AND  (Intervention OR program OR trial) |
| **EBSCO: SportDiscus** | ((cell phones) or (cell*phone*) or (cell-phone) or (mobile phone*) or (mobile-phone) or (mobile device) or (mobile telephone*) or i*Phone* or android or iOS or (mobile health) or mHealth or m-health or app or apps or (smartphone N2 (application or app)) or (mobile N2 (application* or app)) or (information technology intervention) or (IT*intervention) or eHealth or e-health or website or web-based or (web based) or internet-based or (internet based) or internet or tablet-based or (tablet based)) AND  ((resistance N1 (training or fitness)) or (muscle N1 (strength* or fitness or building)) or muscular or strength or strength-balance or (physical* N1 (fit* or train* or activit*)) or (train* N1 (strength* or exercise* or fitness))) AND  (Intervention or program or trial) |
| **OVID: Medline, Embase, Emcare** | ((cell phones) or (cell*phone*) or (cell-phone) or (mobile phone*) or (mobile-phone) or (mobile device) or (mobile telephone*) or i*Phone* or android or iOS or (mobile health) or mHealth or m-health or app or apps or (smartphone adj2 (application or app)) or (mobile adj2 (application* or app)) or (information technology intervention) or (IT*intervention) or eHealth or e-health or website or web-based or (web based) or internet-based or (internet based) or internet or tablet-based or (tablet based)) AND  ((resistance adj (training or fitness)) or (muscle adj (strength* or fitness or building)) or muscular or strength or strength-balance or (physical* adj (fit* or train* or activit*)) or (train* adj (strength* or exercise* or fitness))) AND  (Intervention or program or trial) |
| **Scopus** | (“cell phones” OR “cell*phone*” OR cell-phone OR “mobile phone*” OR mobile-phone OR “mobile device” OR “mobile telephone*” OR i*Phone* OR android OR iOS OR “mobile health” OR mHealth OR m-health OR app OR apps OR smartphone W/2 (application OR app) OR mobile W/2 (application* OR app) OR “information technology intervention” OR “IT*intervention” OR eHealth OR e-health OR website OR web-based OR “web based” OR internet-based OR “internet based” OR internet OR tablet-based OR “tablet based”) AND  (resistance W/1 (training OR fitness) OR muscle W/1 (strength* OR fitness OR building) OR muscular OR strength OR strength-balance OR physical* W/1 (fit* OR train* OR activit*) OR train* W/1 (strength* or exercise* or fitness)) AND  (Intervention OR program OR trial) |
| **Web of Science** | (“cell phones” OR “cell*phone*” OR cell-phone OR “mobile phone*” OR mobile-phone OR “mobile device” OR “mobile telephone*” OR i*Phone* OR android OR iOS OR “mobile health” OR mHealth OR m-health OR app OR apps OR smartphone NEXT/2 (application OR app) OR mobile NEXT/2 (application* OR app) OR “information technology intervention” OR “IT*intervention” OR eHealth OR e-health OR website OR web-based OR “web based” OR internet-based OR “internet based” OR internet OR tablet-based OR “tablet based”) AND  (resistance NEXT/1 (training OR fitness) OR muscle NEXT/1 (strength* OR fitness OR building) OR muscular OR strength OR strength-balance OR physical* NEXT/1 (fit* OR train* OR activit*) OR train* NEXT/1 (strength* or exercise* or fitness)) AND  (Intervention OR program OR trial) |
