# Supplementary material for: Effects of mHealth interventions to prescribe resistance training: a systematic review and meta-analysis of randomized controlled trials
Source: Int J Behav Nutr Phys Act. 2025 Dec 22;23:7. doi: 10.1186/s12966-025-01868-8 (PMC12836956; doi:10.1186/s12966-025-01868-8)
Supplement: Supplementary file 5 — Supplementary Material 5. [file 12966_2025_1868_MOESM5_ESM.docx]

**Effects of mHealth Interventions to Prescribe Resistance Training: A Systematic Review and Meta-Analysis of Randomized Controlled Trials**

**Sports Medicine**

Emily R. Cox, Sam Beacroft, Anna K. Jansson, Levi Wade, Mitch J. Duncan, David R. Lubans, Sara L. Robards, Manuel Leitner, Niklas Gutberlet, Ronald C. Plotnikoff^*^

Ron Plotnikoff, [ron.plotnikoff@newcastle.edu.au](mailto:ron.plotnikoff@newcastle.edu.au)

ATC314*,* University Drive*,* Callaghan, NSW 2308*,* Australia

Tel: +61 (02) 49854465

Fax: 61 + 2 49212084

**TABLE S2. Studies excluded at full-text**

|  | **Author, year** | **Title** | **Reason for exclusion** |
| --- | --- | --- | --- |
| 1 | Adams, 2022 | Adaptive Goals and Reinforcement Timing to Increase Physical Activity in Adults: A Factorial Randomized Trial | Wrong intervention - no resistance training in intervention |
| 2 | Akinci, 2018 | The effects of Internet-based exercise compared with supervised group exercise in people with type 2 diabetes: a randomized controlled study | Wrong outcome |
| 3 | Albright, 2014 | Effectiveness of a 12-month randomized clinical trial to increase physical activity in multiethnic postpartum women: Results from Hawaii's N Mikimiki Project | Wrong intervention - no resistance training in intervention |
| 4 | Al-Eisa, 2016 | Effect of Motivation by "Instagram" on Adherence to Physical Activity among Female College Students | Wrong intervention - no resistance training in intervention |
| 5 | Alexander, 2010 | Internet-delivered behavior change program increases physical activity and improves cardiometabolic disease risk factors in sedentary adults: Results of a randomized controlled trial | Wrong intervention - no resistance training in intervention |
| 6 | Al-Kuwari, 2021 | A comparative study on the uptake and physical activity outcome of the pedometer and smartphone application in Qatar | Wrong intervention - no resistance training in intervention |
| 7 | Allam, 2015 | The effect of social support features and gamification on a Web-based intervention for rheumatoid arthritis patients: randomized controlled trial | Wrong intervention - no resistance training in intervention |
| 8 | Allen, 2013 | Randomized controlled pilot study testing use of smartphone technology for obesity treatment | Wrong intervention - no resistance training in intervention |
| 9 | Allen, 2021 | Stepped exercise program for patients with knee osteoarthritis a randomized controlled trial | Wrong intervention - no resistance training in intervention |
| 10 | Alley, 2014 | Do personally tailored videos in a web-based physical activity intervention lead to higher attention and recall? - an eye-tracking study | Wrong intervention - no resistance training in intervention |
| 11 | Alley, 2016 | Web-Based Video-Coaching to Assist an Automated Computer-Tailored Physical Activity Intervention for Inactive Adults: A Randomized Controlled Trial | Wrong intervention - no resistance training in intervention |
| 12 | Alley, 2018 | The effectiveness of a web 2.0 physical activity intervention in older adults - a randomised controlled trial | Wrong intervention - no resistance training in intervention |
| 13 | Alley, 2022 | The Effectiveness of a Computer-Tailored Web-Based Physical Activity Intervention Using Fitbit Activity Trackers in Older Adults (Active for Life): Randomized Controlled Trial | Wrong outcome |
| 14 | Alley, 2023 | Does matching a personally tailored physical activity intervention to participants' learning style improve intervention effectiveness and engagement? | Wrong intervention - no resistance training in intervention |
| 15 | Al-Nawaiseh, 2022 | An-m-Health Intervention Using Smartphone App to Improve Physical Activity in College Students: A Randomized Controlled Trial | Wrong intervention - no resistance training in intervention |
| 16 | Alonso-Dominguez, 2019 | Effect of a multifactorial intervention on the increase in physical activity in subjects with type 2 diabetes mellitus: a randomized clinical trial (EMID Study) | Wrong intervention - no resistance training in intervention |
| 17 | Alshahrani, 2021 | Whatsapp-based intervention for promoting physical activity among female college students, Saudi Arabia: A randomized controlled trial | Wrong intervention - no resistance training in intervention |
| 18 | Amorim, 2019 | Integrating Mobile-health, health coaching, and physical activity to reduce the burden of chronic low back pain trial (IMPACT): a pilot randomised controlled trial | Wrong intervention - no resistance training in intervention |
| 19 | Anand, 2021 | Group-Based Exercise in CKD Stage 3b to 4: A Randomized Clinical Trial | Wrong intervention - resistance training not prescribed via mHealth |
| 20 | Ansari, 2022 | Comparing the effects of text messaging and mobile social networking on physical activity and anthropometric indices of middle-aged women: a randomized controlled trial | Wrong intervention - no resistance training in intervention |
| 21 | Antypas, 2014 | An Internet- and mobile-based tailored intervention to enhance maintenance of physical activity after cardiac rehabilitation: short-term results of a randomized controlled trial | Wrong intervention - no resistance training in intervention |
| 22 | Arbillaga-Etxarri, 2018 | Long-term efficacy and effectiveness of a behavioural and community-based exercise intervention (Urban Training) to increase physical activity in patients with COPD: A randomised controlled trial | Wrong intervention - no resistance training in intervention |
| 23 | Astrid Peels, 2013 | Long-term efficacy of a printed or a Web-based tailored physical activity intervention among older adults | Wrong intervention - no resistance training in intervention |
| 24 | Atorkey, 2021 | Uptake of Proactively Offered Online and Telephone Support Services Targeting Multiple Health Risk Behaviors Among Vocational Education Students: Process Evaluation of a Cluster Randomized Controlled Trial | Wrong intervention - no resistance training in intervention |
| 25 | Auerswald, 2022 | Impact of Activity Tracker Usage in Combination with a Physical Activity Intervention on Physical and Cognitive Parameters in Healthy Adults Aged 60+: A Randomized Controlled Trial | Wrong intervention - no resistance training in intervention |
| 26 | Backer, 2021 | App-based rehabilitation program after total knee arthroplasty: a randomized controlled trial | Wrong intervention - no resistance training in intervention |
| 27 | Baez, 2017 | Effects of online group exercises for older adults on physical, psychological and social wellbeing: A randomized pilot trial | Wrong intervention - no resistance training in intervention |
| 28 | Ballin, 2020 | Web-based exercise versus supervised exercise for decreasing visceral adipose tissue in older adults with central obesity: a randomized controlled trial | Wrong outcome |
| 29 | Bannell, 2023 | Adherence to unsupervised exercise in sedentary individuals: A randomised feasibility trial of two mobile health interventions | Wrong intervention - not mHealth |
| 30 | Banos, 2015 | An internet-based self-administered intervention for promoting healthy habits and weight loss in hypertensive people who are overweight or obese: a randomized controlled trial | Wrong intervention - no resistance training in intervention |
| 31 | Barreto, 2021 | A Web-Based Multidomain Lifestyle Intervention for Older Adults: The eMIND Randomized Controlled Trial | Wrong intervention - no resistance training in intervention |
| 32 | Barwais, 2013 | Physical activity, sedentary behavior and total wellness changes among sedentary adults: A 4-week randomized controlled trial | Wrong intervention - no resistance training in intervention |
| 33 | Bennell, 2017 | Effectiveness of an internet-delivered exercise and pain-coping skills training intervention for persons with chronic knee pain: A randomized trial | Wrong intervention - resistance training not prescribed via mHealth |
| 34 | Bentley, 2020 | The Use of a Smartphone App and an Activity Tracker to Promote Physical Activity in the Management of Chronic Obstructive Pulmonary Disease: Randomized Controlled Feasibility Study | Wrong intervention - no resistance training in intervention |
| 35 | Berglind, 2020 | The Effect of Smartphone Apps Versus Supervised Exercise on Physical Activity, Cardiorespiratory Fitness, and Body Composition Among Individuals With Mild-to-Moderate Mobility Disability: Randomized Controlled Trial | Wrong outcome |
| 36 | Bernardelli, 2020 | Physical Activity in Healthcare Workers With Low Back Pain Effects of the Back-FIT Randomized Trial | Wrong intervention - not mHealth |
| 37 | Bernardo, 2024 | Feasibility, clinical efficacy, and maternal outcomes of a remote exercise program in pregnant women with obesity: The grob randomized control pilot study | Wrong outcome |
| 38 | Bittar, 2016 | Physical exercises with free weights and elastic bands can improve body composition parameters in postmenopausal women: WEB protocol with a randomized controlled trial | Wrong intervention - not mHealth |
| 39 | Bittel, 2017 | Improving Exercise Performance with an Accelerometer-Based Smartphone App: A Randomized Controlled Trial | Wrong study design |
| 40 | Blackford, 2015 | A randomised controlled trial of a physical activity and nutrition program targeting middle-aged adults at risk of metabolic syndrome in a disadvantaged rural community | Wrong intervention - resistance training not prescribed via mHealth |
| 41 | Blair, 2021 | A home-based mobile health intervention to replace sedentary time with light physical activity in older cancer survivors: Randomized controlled pilot trial | Wrong intervention - no resistance training in intervention |
| 42 | Blake, 2019 | Move-it: A cluster-randomised digital worksite exercise intervention in China: Outcome and process evaluation | Wrong intervention - no resistance training in intervention |
| 43 | Blanquero, 2020 | Feedback-guided exercises performed on a tablet touchscreen improve return to work, function, strength and healthcare usage more than an exercise program prescribed on paper for people with wrist, hand or finger injuries: a randomised trial | Wrong intervention - no resistance training in intervention |
| 44 | Blomqvist, 2025 | Testing the Recruitment Frequency, Implementation Fidelity, and Feasibility of Outcomes of the Heart Failure Activity Coach Study (HEALTHY): Pilot Randomized Controlled Trial | Wrong intervention - no resistance training in intervention |
| 45 | Bohlen, 2024 | Six-Month Outcomes of a Theory- and Technology-Enhanced Physical Activity Intervention for Latina Women (Pasos Hacia La Salud II): Randomized Controlled Trial | Wrong intervention - no resistance training in intervention |
| 46 | Bonato, 2020 | A Mobile Application for Exercise Intervention in People Living with HIV | Wrong intervention - no resistance training in intervention |
| 47 | Bondesson, 2022 | Utilization of healthcare and prescription medicines after non-pharmacological interventions for depression - A 3-year register follow-up of an RCT in primary care | Wrong intervention - no resistance training in intervention |
| 48 | Bonn, 2018 | App-technology to increase physical activity among patients with diabetes type 2 - The DiaCert-study, a randomized controlled trial | Wrong intervention - no resistance training in intervention |
| 49 | Bort-Roig, 2020 | An mHealth workplace-based "sit less, move more" program: Impact on employees' sedentary and physical activity patterns at work and away from work | Wrong intervention - no resistance training in intervention |
| 50 | Bosak, 2010 | Effects of an Internet physical activity intervention in adults with metabolic syndrome | Wrong intervention - no resistance training in intervention |
| 51 | Bossen, 2013 | Effectiveness of a web-based physical activity intervention in patients with knee and/or hip osteoarthritis: randomized controlled trial | Wrong intervention - no resistance training in intervention |
| 52 | Bossen, 2013 | Adherence to a web-based physical activity intervention for patients with knee and/or hip osteoarthritis: a mixed method study | Wrong intervention - no resistance training in intervention |
| 53 | Bouchi, 2024 | Internet of things-based approach for glycemic control in people with type 2 diabetes: A randomized controlled trial | Wrong intervention - no resistance training in intervention |
| 54 | Boudreau, 2020 | Effectiveness of a web-based computer-tailored intervention promoting physical activity for adults from Quebec City: a randomized controlled trial | Wrong intervention - no resistance training in intervention |
| 55 | Broekhuizen, 2016 | An Internet-based physical activity intervention to improve quality of life of inactive older adults: A randomized controlled trial | Wrong intervention - no resistance training in intervention |
| 56 | Brown, 2010 | The effects of internet-based home training on upper limb function in adults with cerebral palsy | Wrong intervention - no resistance training in intervention |
| 57 | Brunet, 2020 | Motivation Predicts Change in Nurses' Physical Activity Levels During a Web-Based Worksite Intervention: Results From a Randomized Trial | Wrong intervention - no resistance training in intervention |
| 58 | Brunet, 2021 | Work-related factors predict changes in physical activity among nurses participating in a web-based worksite intervention: A randomized controlled trial | Wrong intervention - no resistance training in intervention |
| 59 | Burke, 2012 | Using mHealth technology to enhance self-monitoring for weight loss: A randomized trial | Wrong intervention - no resistance training in intervention |
| 60 | Busch, 2020 | The influence of fitness-app usage on psychological well-being and body awareness - A daily diary randomized trial | Wrong intervention - no resistance training in intervention |
| 61 | Cadmus-Bertram, 2013 | Web-based self-monitoring for weight loss among overweight/obese women at increased risk for breast cancer: the HELP pilot study | Wrong intervention - no resistance training in intervention |
| 62 | Cadmus-Bertram, 2015 | Randomized Trial of a Fitbit-Based Physical Activity Intervention for Women | Wrong intervention - no resistance training in intervention |
| 63 | Cadmus-Bertram, 2016 | Randomized trial of a phone- and web-based weight loss program for women at elevated breast cancer risk: the HELP study | Wrong intervention - not mHealth |
| 64 | Cai, 2022 | Effects of peer support and mobile application-based walking programme on physical activity and physical function in rural older adults: a cluster randomized controlled trial | Wrong intervention - no resistance training in intervention |
| 65 | Cai, 2022 | A novel model of home-based, patient-tailored and mobile application-guided cardiac telerehabilitation in patients with atrial fibrillation: A randomised controlled trial | Wrong outcome |
| 66 | Cairo, 2020 | Evaluation of a Mobile Health Intervention to Improve Wellness Outcomes for Breast Cancer Survivors | Wrong intervention - no resistance training in intervention |
| 67 | Callisaya, 2021 | A novel cognitive-motor exercise program delivered via a tablet to improve mobility in older people with cognitive impairment - StandingTall Cognition and Mobility | Wrong intervention - no resistance training in intervention |
| 68 | Cameron, 2015 | A theory-based online health behaviour intervention for new university students (U@Uni: LifeGuide): Results from a repeat randomized controlled trial | Wrong intervention - no resistance training in intervention |
| 69 | Carfora, 2022 | Affective components in promoting physical activity: A randomized controlled trial of message framing | Wrong intervention - no resistance training in intervention |
| 70 | Carpenter, 2022 | Consistency With and Disengagement From Self-monitoring of Weight, Dietary Intake, and Physical Activity in a Technology-Based Weight Loss Program: Exploratory Study | Wrong intervention - no resistance training in intervention |
| 71 | Carr, 2013 | Multicomponent intervention to reduce daily sedentary time: A randomised controlled trial | Wrong intervention - no resistance training in intervention |
| 72 | Carr, 2013 | Randomized controlled trial testing an internet physical activity intervention for sedentary adults | Wrong intervention - no resistance training in intervention |
| 73 | Carter, 2013 | Adherence to a smartphone application for weight loss compared to website and paper diary: pilot randomized controlled trial | Wrong intervention - no resistance training in intervention |
| 74 | Castle, 2022 | The Feasibility and User-Experience of a Digital Health Intervention Designed to Prevent Weight Gain in New Kidney Transplant Recipients-The ExeRTiOn2 Trial | Wrong intervention - no resistance training in intervention |
| 75 | Cavallo, 2012 | A social media-based physical activity intervention: A randomized controlled trial | Wrong intervention - not mHealth |
| 76 | Cavallo, 2014 | The role of companionship, esteem, and informational support in explaining physical activity among young women in an online social network intervention | Wrong intervention - no resistance training in intervention |
| 77 | Chan, 2020 | Feasibility and acceptability of a remotely delivered, web-based behavioral intervention for men with prostate cancer: Four-arm randomized controlled pilot trial | Wrong intervention - no resistance training in intervention |
| 78 | Chan, 2024 | Cultural Adaptation of the Younger Women's Wellness after Cancer Program for Younger Chinese Women with Breast Cancer: A Pilot Randomized Controlled Trial | Wrong intervention - no resistance training in intervention |
| 79 | Chaplin, 2017 | Interactive web-based pulmonary rehabilitation programme: a randomised controlled feasibility trial | Wrong outcome |
| 80 | Chee, 2014 | A Randomised Controlled Trial of a Facebook-based Physical Activity Intervention for Government Employees with Metabolic Syndrome | Wrong intervention - no resistance training in intervention |
| 81 | Chee, 2019 | Decreasing sleep-related symptoms through increasing physical activity among Asian American midlife women | Wrong intervention - not mHealth |
| 82 | Chee, 2020 | Effect of An Online Physical Activity Promotion Program and Cardiovascular Symptoms Among Asian American Women at Midlife | Wrong study design |
| 83 | Chen, 2022 | Effectiveness of a nurse-led mHealth app to prevent excessive gestational weight gain among overweight and obese women: A randomized controlled trial | Wrong intervention - no resistance training in intervention |
| 84 | Chen, 2024 | Effects of an mHealth intervention on maternal and infant outcomes from pregnancy to early postpartum for women with overweight or obesity: A randomized controlled trial | Wrong intervention - no resistance training in intervention |
| 85 | Cheng, 2022 | Use of mobile app to enhance functional outcomes and adherence of home-based rehabilitation program for elderly with hip fracture: A randomized controlled trial | Wrong outcome |
| 86 | Cheng, 2023 | Effect of an mHealth weight loss intervention on Healthy Eating Index diet quality: the SMARTER randomised controlled trial | Wrong intervention - no resistance training in intervention |
| 87 | Chhabra, 2018 | Smartphone app in self-management of chronic low back pain: a randomized controlled trial | Wrong intervention - no resistance training in intervention |
| 88 | Chiang, 2023 | Effectiveness of a 12â€week teleâ€exercise training program on cardiorespiratory fitness and heart rate recovery in patients with cardiometabolic multimorbidity | Wrong intervention - no resistance training in intervention |
| 89 | Choi, 2016 | mHealth Physical Activity Intervention: A Randomized Pilot Study in Physically Inactive Pregnant Women | Wrong intervention - no resistance training in intervention |
| 90 | Choi, 2021 | A Pilot Study to Promote Active Living among Physically Inactive Korean American Women | Wrong intervention - resistance training not prescribed via mHealth |
| 91 | Choi, 2023 | Effects of a Mobile-Health Exercise Intervention on Body Composition, Vascular Function, and Autonomic Nervous System Function in Obese Women: A Randomized Controlled Trial | Wrong outcome |
| 92 | Chow, 2021 | Feasibility of a behavioral intervention using mobile health applications to reduce cardiovascular risk factors in cancer survivors: a pilot randomized controlled trial | Wrong intervention - no resistance training in intervention |
| 93 | Chughtai, 2019 | The Role of Virtual Rehabilitation in Total and Unicompartmental Knee Arthroplasty | Wrong intervention - resistance training not prescribed via mHealth |
| 94 | Chung, 2017 | Tweeting to Health: A Novel mHealth Intervention Using Fitbits and Twitter to Foster Healthy Lifestyles | Wrong intervention - no resistance training in intervention |
| 95 | Chung, 2020 | Exercise Promotion and Distress Reduction Using a Mobile App-Based Community in Breast Cancer Survivors | Wrong intervention - no resistance training in intervention |
| 96 | Cinar, 2021 | Effect of mobile phone app-based training on the quality of life for women with breast cancer | Wrong intervention - no resistance training in intervention |
| 97 | Clausen, 2020 | Improving Maximal Strength in the Initial Postoperative Phase After Anterior Cruciate Ligament Reconstruction Surgery: Randomized Controlled Trial of an App-Based Serious Gaming Approach | Wrong intervention - no resistance training in intervention |
| 98 | Collins, 2010 | Evaluation of a commercial web-based weight loss and weight loss maintenance program in overweight and obese adults: a randomized controlled trial | Wrong intervention - no resistance training in intervention |
| 99 | Collombon, 2024 | The efficacy of online physical activity interventions with added mobile elements within adults aged 50 years and over: Randomized controlled trial | Wrong intervention - no resistance training in intervention |
| 100 | Compernolle, 2015 | Effectiveness of a web-based, computer-tailored, pedometer-based physical activity intervention for adults: a cluster randomized controlled trial | Wrong intervention - no resistance training in intervention |
| 101 | Conroy, 2021 | HeartPhone: Mobile Evaluative Conditioning to Enhance Affective Processes and Promote Physical Activity | Wrong intervention - no resistance training in intervention |
| 102 | Cook, 2015 | A Web-based health promotion program for older workers: randomized controlled trial | Wrong intervention - no resistance training in intervention |
| 103 | Cowdery, 2015 | Exergame Apps and Physical Activity: the Results of the ZOMBIE Trial | Wrong intervention - no resistance training in intervention |
| 104 | Cox, 2017 | Comparison of Internet and Telephone Interventions for Weight Loss Among Cancer Survivors: Randomized Controlled Trial and Feasibility Study | Wrong intervention - no resistance training in intervention |
| 105 | Cox, 2023 | Web-based physical activity promotion in young people with CF: a randomised controlled trial | Wrong intervention - no resistance training in intervention |
| 106 | Cox, 2023 | Increasing Physical Activity in Empty Nest and Retired Populations Online: A Randomized Feasibility Study | Wrong intervention - no resistance training in intervention |
| 107 | Crane, 2015 | A randomized trial testing the efficacy of a novel approach to weight loss among men with overweight and obesity | Wrong intervention - no resistance training in intervention |
| 108 | Cruvinel Junior, 2021 | Foot-ankle functional outcomes of using the Diabetic Foot Guidance System (SOPeD) for people with diabetic neuropathy: a feasibility study for the single-blind randomized controlled FOotCAre (FOCA) trial I | Wrong intervention - no resistance training in intervention |
| 109 | Dadaczynski, 2017 | Promoting physical activity in worksite settings: results of a German pilot study of the online intervention Healingo fit | Wrong intervention - no resistance training in intervention |
| 110 | Damschroder, 2020 | Effect of adding telephone-based brief coaching to an mHealth app (stay strong) for promoting physical activity among veterans: Randomized controlled trial | Wrong intervention - no resistance training in intervention |
| 111 | Davis, 2021 | An observation of the impact of tailored messages on participant non-compliance during Desire2Move | Wrong intervention - no resistance training in intervention |
| 112 | DeCocker, 2012 | Web-based, computer-tailored, pedometer-based physical activity advice: development, dissemination through general practice, acceptability, and preliminary efficacy in a randomized controlled trial | Wrong intervention - no resistance training in intervention |
| 113 | Dekker-van Weering, 2017 | User Experience, Actual Use, and Effectiveness of an Information Communication Technology-Supported Home Exercise Program for Pre-Frail Older Adults | Wrong outcome |
| 114 | Delbaere, 2021 | E-health StandingTall balance exercise for fall prevention in older people: results of a two year randomised controlled trial | Wrong intervention - no resistance training in intervention |
| 115 | delPozo-Cruz, 2012 | AN OCCUPATIONAL, INTERNET-BASED INTERVENTION TO PREVENT CHRONICITY IN SUBACUTE LOWER BACK PAIN: A RANDOMIZED CONTROLLED TRIAL | Wrong outcome |
| 116 | delPozo-Cruz, 2013 | Clinical effects of a nine-month web-based intervention in subacute non-specific low back pain patients: a randomized controlled trial | Wrong outcome |
| 117 | Demark-Wahnefried, 2023 | Results of DUET: A Web-Based Weight Loss Randomized Controlled Feasibility Trial among Cancer Survivors and Their Chosen Partners | Wrong intervention - no resistance training in intervention |
| 118 | Demeyer, 2017 | Physical activity is increased by a 12-week semiautomated telecoaching programme in patients with COPD: a multicentre randomised controlled trial | Wrong intervention - resistance training not prescribed via mHealth |
| 119 | Devi, 2014 | A web-based program improves physical activity outcomes in a primary care angina population: randomized controlled trial | Wrong intervention - no resistance training in intervention |
| 120 | Devine, 2020 | Feasibility of FitSurvivor: A technology-enhanced group-based fitness intervention for adolescent and young adult survivors of childhood cancer | Wrong population |
| 121 | Dharmawan, 2018 | Web-Based Application to Support Physical Fitness Information of Elderly People | Wrong intervention - resistance training not prescribed via mHealth |
| 122 | Dlugonski, 2011 | Increasing physical activity in multiple sclerosis: Replicating internet intervention effects using objective and self-report outcomes | Wrong intervention - no resistance training in intervention |
| 123 | Dlugonski, 2012 | Internet-delivered behavioral intervention to increase physical activity in persons with multiple sclerosis: sustainability and secondary outcomes | Wrong intervention - resistance training not prescribed via mHealth |
| 124 | Domal, 2023 | Influence of smartphone-based physical activity intervention on executive functions and cardiometabolic disease risk in obese young adults: a pilot randomised controlled trial | Wrong intervention - no resistance training in intervention |
| 125 | Dong, 2019 | The effects of the combined exercise intervention based on internet and social media software (CEIBISMS) on quality of life, muscle strength and cardiorespiratory capacity in Chinese postoperative breast cancer patients:a randomized controlled trial | Wrong intervention - not mHealth |
| 126 | Dor-Haim, 2019 | A Novel Digital Platform for a Monitored Home-based Cardiac Rehabilitation Program | Wrong intervention - not mHealth |
| 127 | Downs, 2021 | Adaptive, behavioral intervention impact on weight gain, physical activity, energy intake, and motivational determinants: results of a feasibility trial in pregnant women with overweight/obesity | Wrong intervention - resistance training not prescribed via mHealth |
| 128 | Drew, 2022 | Mechanisms of an eHealth program targeting depression in men with overweight or obesity: A randomised trial | Wrong intervention - resistance training not prescribed via mHealth |
| 129 | Duan, 2017 | Web-Based Intervention for Physical Activity and Fruit and Vegetable Intake Among Chinese University Students: A Randomized Controlled Trial | Wrong intervention - no resistance training in intervention |
| 130 | Duan, 2018 | Evaluation of a Web-Based Intervention for Multiple Health Behavior Changes in Patients With Coronary Heart Disease in Home-Based Rehabilitation: Pilot Randomized Controlled Trial | Wrong intervention - no resistance training in intervention |
| 131 | Duan, 2022 | The Effectiveness of Sequentially Delivered Web-Based Interventions on Promoting Physical Activity and Fruit-Vegetable Consumption among Chinese College Students: Mixed Methods Study | Wrong intervention - no resistance training in intervention |
| 132 | Dulin, 2023 | The Hombres Saludables Physical Activity Web-Based and Mobile Phone Intervention: Pilot Randomized Controlled Trial With Latino Men | Wrong intervention - no resistance training in intervention |
| 133 | Duncan, 2016 | Balanced: a randomised trial examining the efficacy of two self-monitoring methods for an app-based multi-behaviour intervention to improve physical activity, sitting and sleep in adults | Wrong intervention - no resistance training in intervention |
| 134 | Duncan, 2020 | Efficacy of a multi-component m-health weight-loss intervention in overweight and obese adults: A randomised controlled trial | Wrong intervention - no resistance training in intervention |
| 135 | Duscha, 2018 | Effects of a 12-Week mHealth Program on Functional Capacity and Physical Activity in Patients With Peripheral Artery Disease | Wrong intervention - no resistance training in intervention |
| 136 | Duscha, 2018 | Effects of a 12-week mHealth program on peak VO<sub>2</sub> and physical activity patterns after completing cardiac rehabilitation: A randomized controlled trial | Wrong intervention - no resistance training in intervention |
| 137 | Eckardt, 2021 | Smartphone-guided secondary prevention for patients with coronary artery disease | Wrong intervention - no resistance training in intervention |
| 138 | Edney, 2019 | User Engagement and Attrition in an App-Based Physical Activity Intervention: Secondary Analysis of a Randomized Controlled Trial | Wrong intervention - no resistance training in intervention |
| 139 | Edney, 2020 | A Social Networking and Gamified App to Increase Physical Activity: Cluster RCT | Wrong intervention - no resistance training in intervention |
| 140 | Ehlers, 2015 | Can an evidence-based book club intervention delivered via a tablet computer improve physical activity in middle-aged women? | Wrong intervention - no resistance training in intervention |
| 141 | Ek, 2020 | Effectiveness of a 3-Month Mobile Phone-Based Behavior Change Program on Active Transportation and Physical Activity in Adults: Randomized Controlled Trial | Wrong intervention - no resistance training in intervention |
| 142 | Eldoen, 2021 | Web-based vestibular rehabilitation in persistent postural-perceptual dizziness | Wrong intervention - no resistance training in intervention |
| 143 | Elliott, 2019 | Physical Activity Behavior Change Driven by Engagement With an Incentive-Based App: Evaluating the Impact of Sweatcoin | Wrong intervention - no resistance training in intervention |
| 144 | Ellis, 2019 | Comparative effectiveness of mhealth-supported exercise compared with exercise alone for people with Parkinson disease: Randomized controlled pilot study | Wrong outcome |
| 145 | Elloumi, 2018 | Exploratory study of a virtual community for physical activity | Wrong intervention - no resistance training in intervention |
| 146 | Engelen, 2020 | Evaluation of a web-based self-management program for patients with cardiovascular disease: Explorative randomized controlled trial | Wrong intervention - no resistance training in intervention |
| 147 | Ester, 2023 | Effectiveness of a Self-Monitoring App in Supporting Physical Activity Maintenance Among Rural Canadians With Cancer After an Exercise Oncology Program: Cluster Randomized Controlled Trial | Wrong intervention - not mHealth |
| 148 | Evans, 2021 | Acceptability and preliminary efficacy of a web-and telephone-based personalised exercise intervention for individuals with metastatic prostate cancer: The exerciseguide pilot randomised controlled trial | Wrong intervention - no resistance training in intervention |
| 149 | Fanning, 2017 | A smartphone 'app'-delivered randomized factorial trial targeting physical activity in adults | Wrong intervention - no resistance training in intervention |
| 150 | Fanning, 2022 | The Effects of a Pain Management-Focused Mobile Health Behavior Intervention on Older Adults' Self-efficacy, Satisfaction with Functioning, and Quality of Life: a Randomized Pilot Trial | Wrong intervention - no resistance training in intervention |
| 151 | Felix, 2020 | Sweat and Hair: Online Interventions for Improving Physical Activity in African-American Women | Wrong intervention - no resistance training in intervention |
| 152 | Felker, 2022 | A Randomized Controlled Trial of Mobile Health Intervention in Patients With Heart Failure and Diabetes | Wrong intervention - no resistance training in intervention |
| 153 | Feng, 2019 | Feasibility of an at-home, web-based, interactive exercise program for older adults | Wrong intervention - no resistance training in intervention |
| 154 | Ferrando-Terradez, 2024 | Adherence Patterns and Health Outcomes in Spanish Young Women Participating in a Virtual-Guided HIIT Program: insights from the Randomized Controlled WISE Trial | Wrong intervention - no resistance training in intervention |
| 155 | Ferrante, 2022 | Patterns of Fitbit Use and Activity Levels Among African American Breast Cancer Survivors During an eHealth Weight Loss Randomized Controlled Trial | Wrong intervention - no resistance training in intervention |
| 156 | Ferrara, 2015 | Feasibility of a social networking site to promote physical activity in adults | Wrong intervention - no resistance training in intervention |
| 157 | Fichtner, 2024 | Effects of a digital intervention on physical activity in adults: A randomized controlled trial in a large-scale sample | Wrong outcome |
| 158 | Fillol, 2022 | Possible Impact of a 12-Month Web- And Smartphone-Based Program to Improve Long-term Physical Activity in Patients Attending Spa Therapy: Randomized Controlled Trial | Wrong outcome |
| 159 | Finkelstein, 2025 | Effectiveness and cost-effectiveness of an app and rewards-based intervention in type 2 diabetes: A randomised controlled trial | Wrong intervention - no resistance training in intervention |
| 160 | Finlay, 2020 | Optimising web-based computer-tailored physical activity interventions for prostate cancer survivors: A randomised controlled trial examining the impact of website architecture on user engagement | Wrong intervention - no resistance training in intervention |
| 161 | Flachenecker, 2020 | Efficacy of an internet-based program to promote physical activity and exercise after inpatient rehabilitation in persons with multiple sclerosis: A randomized, single-blind, controlled study | Wrong outcome |
| 162 | Forbes, 2015 | Feasibility and Preliminary Efficacy of an Online Intervention to Increase Physical Activity in Nova Scotian Cancer Survivors: A Randomized Controlled Trial | Wrong intervention - resistance training not prescribed via mHealth |
| 163 | Forbes, 2017 | A pilot study on the motivational effects of an internet-delivered physical activity behaviour change programme in Nova Scotian cancer survivors | Wrong intervention - no resistance training in intervention |
| 164 | Frei, 2019 | A novel approach to increase physical activity in older adults in the community using citizen science: a mixed-methods study | Wrong intervention - no resistance training in intervention |
| 165 | Frias, 2017 | Effectiveness of Digital Medicines to Improve Clinical Outcomes in Patients with Uncontrolled Hypertension and Type 2 Diabetes: Prospective, Open-Label, Cluster-Randomized Pilot Clinical Trial | Wrong intervention - no resistance training in intervention |
| 166 | Friederichs, 2014 | Motivational interviewing in a Web-based physical activity intervention with an avatar: randomized controlled trial | Wrong intervention - no resistance training in intervention |
| 167 | Friederichs, 2015 | Long term effects of self-determination theory and motivational interviewing in a web-based physical activity intervention: Randomized controlled trial | Wrong intervention - no resistance training in intervention |
| 168 | Friederichs, 2016 | Exploring the working mechanisms of a web-based physical activity intervention, based on self-determination theory and motivational interviewing | Wrong intervention - no resistance training in intervention |
| 169 | Friederichs, 2016 | Motivational interviewing and self-determination theory in a web-based computer tailored physical activity intervention: A randomized controlled trial | Wrong intervention - no resistance training in intervention |
| 170 | Fruhwirth, 2022 | Evaluation of a Newly Developed Smartphone App for Risk Factor Management in Young Patients With Ischemic Stroke: A Pilot Study | Wrong intervention - no resistance training in intervention |
| 171 | Gabbiadini, 2019 | Fitness mobile apps positively affect attitudes, perceived behavioral control and physical activities | Wrong intervention - no resistance training in intervention |
| 172 | Galdiz, 2021 | Telerehabilitation Programme as a Maintenance Strategy for COPD Patients: A 12-Month Randomized Clinical Trial | Wrong outcome |
| 173 | Galiano-Castillo, 2016 | Telehealth system: A randomized controlled trial evaluating the impact of an internet-based exercise intervention on quality of life, pain, muscle strength, and fatigue in breast cancer survivors | Wrong intervention - not mHealth |
| 174 | Ganesan, 2453 | International Mobile-Health Intervention on Physical Activity, Sitting, and Weight: The Stepathlon Cardiovascular Health Study | Wrong intervention - no resistance training in intervention |
| 175 | Gao, 2023 | Effects of personalized exercise prescriptions and social media delivered through mobile health on cancer survivors' physical activity and quality of life | Wrong outcome |
| 176 | Garcia-Ortiz, 2018 | Long-term effectiveness of a smartphone app for improving healthy lifestyles in general population in primary care: Randomized controlled trial (evident II study) | Wrong intervention - no resistance training in intervention |
| 177 | Geraghty, 2018 | Using an internet intervention to support self-management of low back pain in primary care: Findings from a randomised controlled feasibility trial (SupportBack) | Wrong intervention - no resistance training in intervention |
| 178 | Ghazala, 2024 | Efficacy of mHealth-Based Workplace Health Promotion Strategy in Improving Cardiorespiratory Fitness in a Healthcare Setting: A Randomized Controlled Study | Wrong outcome |
| 179 | Ghorbani, 2021 | Comparing the Effects of Gamification and Teach-Back Training Methods on Adherence to a Therapeutic Regimen in Patients after Coronary Artery Bypass Graft Surgery: Randomized Clinical Trial | Wrong intervention - no resistance training in intervention |
| 180 | Gill, 2019 | The HealtheSteps TM lifestyle prescription program to improve physical activity and modifiable risk factors for chronic disease: a pragmatic randomized controlled trial | Wrong intervention - no resistance training in intervention |
| 181 | Glasgow, 2010 | Outcomes of minimal and moderate support versions of an internet-based diabetes self-management support program | Wrong intervention - no resistance training in intervention |
| 182 | Glasgow, 2011 | Engagement in a diabetes self-management website: usage patterns and generalizability of program use | Wrong intervention - no resistance training in intervention |
| 183 | Glasgow, 2012 | Twelve-month outcomes of an Internet-based diabetes self-management support program | Wrong intervention - no resistance training in intervention |
| 184 | Glozier, 2013 | Internet-Delivered Cognitive Behavioural Therapy for Adults with Mild to Moderate Depression and High Cardiovascular Disease Risks: A Randomised Attention-Controlled Trial | Wrong intervention - no resistance training in intervention |
| 185 | Glynn, 2014 | Effectiveness of a smartphone application to promote physical activity in primary care: The SMART MOVE randomised controlled trial | Wrong intervention - no resistance training in intervention |
| 186 | Goharinejad, 2024 | Evaluating the effects of mobile application-based rehabilitation on improving disability and pain in patients with disputed thoracic outlet syndrome: A randomized controlled trial | Wrong outcome |
| 187 | Golsteijn, 2017 | A Web-Based and Print-Based Computer-Tailored Physical Activity Intervention for Prostate and Colorectal Cancer Survivors: A Comparison of User Characteristics and Intervention Use | Wrong intervention - resistance training not prescribed via mHealth |
| 188 | Golsteijn, 2018 | Short-term efficacy of a computer-tailored physical activity intervention for prostate and colorectal cancer patients and survivors: A randomized controlled trial | Wrong intervention - no resistance training in intervention |
| 189 | Gomez-Marcos, 2018 | Short- and long-term effectiveness of a smartphone application for improving measures of adiposity: A randomised clinical trial - EVIDENT II study | Wrong intervention - no resistance training in intervention |
| 190 | Gonzalez-Plaza, 2022 | Effectiveness of a Step Counter Smartband and Midwife Counseling Intervention on Gestational Weight Gain and Physical Activity in Pregnant Women With Obesity (Pas and Pes Study): Randomized Controlled Trial | Wrong intervention - no resistance training in intervention |
| 191 | Gonzalez-Sanchez, 2019 | Using a smartphone app in changing cardiovascular risk factors: A randomized controlled trial (EVIDENT II study) | Wrong intervention - no resistance training in intervention |
| 192 | Gonze, 2020 | Use of a Smartphone App to Increase Physical Activity Levels in Insufficiently Active Adults: Feasibility Sequential Multiple Assignment Randomized Trial (SMART) | Wrong intervention - no resistance training in intervention |
| 193 | Goode, 2022 | Perioperative Mobile Telehealth Program for Post-Prostatectomy Incontinence: A Randomized Clinical Trial | Wrong intervention - not mHealth |
| 194 | Gorny, 2022 | Active Use and Engagement in an mHealth Initiative Among Young Men With Obesity: Mixed Methods Study | Wrong intervention - no resistance training in intervention |
| 195 | Goto, 2014 | Self-monitoring has potential for home exercise programmes in patients with haemophilia | Wrong intervention - not mHealth |
| 196 | Gotsis, 2013 | Wellness Partners: Design and evaluation of a web-based physical activity diary with social gaming features for adults | Wrong intervention - no resistance training in intervention |
| 197 | Graham, 2017 | Does Usage of an eHealth Intervention Reduce the Risk of Excessive Gestational Weight Gain? Secondary Analysis From a Randomized Controlled Trial | Wrong intervention - no resistance training in intervention |
| 198 | Grant, 2024 | A web-based physical activity intervention targeting affect regulation: a randomized feasibility trial | Wrong intervention - no resistance training in intervention |
| 199 | Grau-Pellicer, 2019 | Impact of mHealth technology on adherence to healthy PA after stroke: a randomized study | Wrong intervention - no resistance training in intervention |
| 200 | Greene, 2012 | Impact of an online healthful eating and physical activity program for college students | Wrong intervention - no resistance training in intervention |
| 201 | Greene, 2013 | The impact of an online social network with wireless monitoring devices on physical activity and weight loss | Wrong intervention - no resistance training in intervention |
| 202 | Gremaud, 2018 | Gamifying accelerometer use increases physical activity levels of sedentary office workers | Wrong intervention - no resistance training in intervention |
| 203 | Guillaumier, 2022 | Evaluation of an online intervention for improving stroke survivors' health-related quality of life: A randomised controlled trial | Wrong intervention - no resistance training in intervention |
| 204 | Guo, 2020 | Effect of a WeChat-Based intervention (Run4Love) on depressive symptoms among people living with HIV in China: A randomized controlled trial | Wrong intervention - no resistance training in intervention |
| 205 | Guo, 2022 | Effects of a WeChat-based individualized post-discharge rehabilitation program on patients with lumbar fusion surgery | Wrong intervention - resistance training not prescribed via mHealth |
| 206 | Gur, 2020 | The Effect of the ERVE Smartphone App on Physical Activity, Quality of Life, Self-Efficacy, and Exercise Motivation for Inactive People: A Randomized Controlled Trial | Wrong outcome |
| 207 | Haglo, 2021 | Smartphone-Assisted High-Intensity Interval Training in Inflammatory Rheumatic Disease Patients: Randomized Controlled Trial | Wrong intervention - no resistance training in intervention |
| 208 | Hales, 2016 | Social networks for improving healthy weight loss behaviors for overweight and obese adults: A randomized clinical trial of the social pounds off digitally (Social POD) mobile app | Wrong intervention - no resistance training in intervention |
| 209 | Haller, 2018 | Individualized web-Based exercise for the treatment of depression: Randomized controlled trial | Wrong outcome |
| 210 | Hamilton, 2022 | Usability of affordable feedback-based technologies to improve mobility and physical activity in rehabilitation: a mixed methods study | Wrong study design |
| 211 | Hanton, 2017 | Mobile Phone-Based Measures of Activity, Step Count, and Gait Speed: Results From a Study of Older Ambulatory Adults in a Naturalistic Setting | Wrong intervention - no resistance training in intervention |
| 212 | Haque, 2020 | A persuasive mhealth behavioral change intervention for promoting physical activity in the workplace: Feasibility randomized controlled trial | Wrong intervention - no resistance training in intervention |
| 213 | Hardcastle, 2024 | Impact of the Promoting Physical Activity in Regional and Remote Cancer Survivors intervention on health-related quality of life in breast and colorectal cancer survivors | Wrong intervention - not mHealth |
| 214 | Hardt, 3429 | Improved early outcome after TKA through an app-based active muscle training programme-a randomized-controlled trial | Wrong intervention - not mHealth |
| 215 | Harries, 2016 | Effectiveness of a smartphone app in increasing physical activity amongst male adults: a randomised controlled trial | Wrong intervention - no resistance training in intervention |
| 216 | Harris, 2018 | Enhancing Psychosocial Constructs Associated with Technology-Based Physical Activity: A Randomized Trial Among African American Women | Wrong intervention - no resistance training in intervention |
| 217 | Hartman, 2015 | Internet-based physical activity intervention for women with a family history of breast cancer | Wrong intervention - no resistance training in intervention |
| 218 | Hartman, 2017 | Physical activity maintenance among Spanish-speaking Latinas in a randomized controlled trial of an Internet-based intervention | Wrong intervention - no resistance training in intervention |
| 219 | Hassandra, 2017 | An mHealth App for Supporting Quitters to Manage Cigarette Cravings With Short Bouts of Physical Activity: A Randomized Pilot Feasibility and Acceptability Study | Wrong study design |
| 220 | Hastings, 2021 | Video-Enhanced Care Management for Medically Complex Older Adults with Cognitive Impairment | Wrong intervention - not mHealth |
| 221 | Haufe, 2019 | Telemonitoring-supported exercise training, metabolic syndrome severity, and work ability in company employees: a randomised controlled trial | Wrong intervention - no resistance training in intervention |
| 222 | Haufe, 2020 | Employers With Metabolic Syndrome and Increased Depression/Anxiety Severity Profit Most From Structured Exercise Intervention for Work Ability and Quality of Life | Wrong intervention - no resistance training in intervention |
| 223 | Hawkins, 2019 | Acceptability and Feasibility of Implementing Accelorometry-Based Activity Monitors and a Linked Web Portal in an Exercise Referral Scheme: Feasibility Randomized Controlled Trial | Wrong intervention - no resistance training in intervention |
| 224 | Hayman, 2017 | Feasibility, acceptability and efficacy of a web-based computer-tailored physical activity intervention for pregnant women - the Fit4Two randomised controlled trial | Wrong intervention - no resistance training in intervention |
| 225 | Hemnes, 2021 | A Mobile Health Intervention to Increase Physical Activity in Pulmonary Arterial Hypertension | Wrong intervention - no resistance training in intervention |
| 226 | Hernandez-Reyes, 2020 | Push Notifications From a Mobile App to Improve the Body Composition of Overweight or Obese Women: Randomized Controlled Trial | Wrong intervention - no resistance training in intervention |
| 227 | Hernandez-Reyes, 2020 | Effect of an mHealth Intervention Using a Pedometer App With Full In-Person Counseling on Body Composition of Overweight Adults: Randomized Controlled Weight Loss Trial | Wrong intervention - no resistance training in intervention |
| 228 | Hidrus, 2021 | Effects of Brain Breaks Video Intervention of Decisional Balance among Malaysians with Type 2 Diabetes Mellitus: A Randomised Controlled Trial | Wrong intervention - no resistance training in intervention |
| 229 | Hidrus, 2022 | Effects of technology-supported brain breaks videos on exercise self-efficacy among type 2 diabetes mellitus Malaysians | Wrong intervention - no resistance training in intervention |
| 230 | Hochsmann, 2022 | Association between weight loss, change in physical activity, and change in quality of life following a corporately sponsored, online weight loss program | Wrong intervention - no resistance training in intervention |
| 231 | Holmen, 2016 | A mobile health intervention for self-management and lifestyle change for persons with type 2 diabetes, part 2: One-year results from the Norwegian randomized controlled trial RENEWING HEALTH | Wrong intervention - no resistance training in intervention |
| 232 | Holtdirk, 2021 | Results of the Optimune trial: A randomized controlled trial evaluating a novel Internet intervention for breast cancer survivors | Wrong intervention - no resistance training in intervention |
| 233 | Hong, 2023 | Digital Therapeutic Exercises Using Augmented Reality Glasses for Frailty Prevention among Older Adults | Wrong intervention - not mHealth |
| 234 | Hongchuvech, 2021 | Health promotion intervention through smartphone line application for increasing physical activity and healthy eating behavior among overweight women in urban community in Bangkok, Thailand | Wrong intervention - no resistance training in intervention |
| 235 | Hu, 2022 | Supervised mHeath Exercise Improves Health Factors More Than Self-Directed mHealth Exercise: A Clinical Controlled Study | Wrong intervention - no resistance training in intervention |
| 236 | Huang, 2023 | Field Test of an m-Health Worksite Health Promotion Program to Increase Physical Activity in Taiwanese Employees: A Cluster-Randomized Controlled Trial | Wrong intervention - no resistance training in intervention |
| 237 | Hurkmans, 2010 | Maintenance of physical activity after Internet-based physical activity interventions in patients with rheumatoid arthritis | Wrong intervention - no resistance training in intervention |
| 238 | Hurkmans, 2018 | Face-To-face versus mobile versus blended weight loss program: Randomized clinical trial | Wrong intervention - no resistance training in intervention |
| 239 | Hutchesson, 2018 | A Targeted and Tailored eHealth Weight Loss Program for Young Women: The Be Positive Be Healthe Randomized Controlled Trial | Wrong intervention - no resistance training in intervention |
| 240 | Im, 2017 | Improving menopausal symptoms through promoting physical activity: a pilot Web-based intervention study among Asian Americans | Wrong intervention - resistance training not prescribed via mHealth |
| 241 | Innes, 2019 | Evaluating differences in the clinical impact of a free online weight loss programme, a resource-intensive commercial weight loss programme and an active control condition: a parallel randomised controlled trial | Wrong intervention - resistance training not prescribed via mHealth |
| 242 | Irvine, 2011 | Get moving: a web site that increases physical activity of sedentary employees | Wrong intervention - resistance training not prescribed via mHealth |
| 243 | Irwin, 2016 | Testing the Efficacy of OurSpace, a Brief, Group Dynamics-Based Physical Activity Intervention: A Randomized Controlled Trial | Wrong study design |
| 244 | Ismail, 2022 | Design and Evaluation of a Just-in-Time Adaptive Intervention (JITAI) to Reduce Sedentary Behavior at Work: Experimental Study | Wrong intervention - no resistance training in intervention |
| 245 | Itoh, 2022 | Evaluation of the Effect of Patient Education and Strengthening Exercise Therapy Using a Mobile Messaging App on Work Productivity in Japanese Patients With Chronic Low Back Pain: Open-Label, Randomized, Parallel-Group Trial | Wrong outcome |
| 246 | Jacobs, 2011 | Testing an integrated model of the theory of planned behaviour and self-determination theory for different energy balance-related behaviours and intervention intensities | Wrong intervention - no resistance training in intervention |
| 247 | Jahangiry, 2017 | An interactive web-based intervention on nutritional status, physical activity and health-related quality of life in patient with metabolic syndrome: A randomized-controlled trial (The Red Ruby Study) | Wrong intervention - no resistance training in intervention |
| 248 | Jennings, 2014 | Effectiveness of a web-based physical activity intervention for adults with Type 2 diabetes-A randomised controlled trial | Wrong intervention - no resistance training in intervention |
| 249 | Ji, 2019 | Mobile Health Management Platform-Based Pulmonary Rehabilitation for Patients With Non-Small Cell Lung Cancer: Prospective Clinical Trial | Wrong outcome |
| 250 | Ji-Been, 2024 | Improving Cardiorespiratory and Muscular Function of Korean Firefighters A Kinect-Based Mixed Reality Device Exercise Intervention Randomized Control Trial | Wrong intervention - not mHealth |
| 251 | Johansson, 2021 | Trajectories and associations between depression and physical activity in patients with cardiovascular disease during participation in an internet-based cognitive behavioural therapy programme | Wrong intervention - no resistance training in intervention |
| 252 | Jorvand, 2020 | Evaluating the impact of HBM-based education on exercise among health care workers: the usage of mobile applications in Iran | Wrong intervention - no resistance training in intervention |
| 253 | Joseph, 2016 | Results of a Culturally Adapted Internet-Enhanced Physical Activity Pilot Intervention for Overweight and Obese Young Adult African American Women | Wrong intervention - no resistance training in intervention |
| 254 | Juhlin, 2021 | Physical activity with person-centred guidance supported by a digital platform for persons with chronic widespread pain: A randomized controlled trial | Wrong intervention - resistance training not prescribed via mHealth |
| 255 | Jungreitmayr, 2021 | Effects of an Information and Communication Technology-Based Fitness Program on Strength and Balance in Female Home Care Service Users | Wrong study design |
| 256 | Kaneda, 2024 | Impact of the simultaneous distribution of e-learning and exercise videos on the health literacy and lifestyle of college students during the COVID-19 pandemic: a randomized controlled trial | Wrong intervention - no resistance training in intervention |
| 257 | Kanera, 2016 | Use and appreciation of a tailored self-management ehealth intervention for early cancer survivors: Process evaluation of a randomized controlled trial | Wrong intervention - no resistance training in intervention |
| 258 | Kang, 2014 | A web-based health promotion program for patients with metabolic syndrome | Wrong intervention - no resistance training in intervention |
| 259 | Kang, 2021 | Efficacy of Health Coaching and an Electronic Health Management Program: Randomized Controlled Trial | Wrong intervention - no resistance training in intervention |
| 260 | Kantorowski, 2018 | Determinants and outcomes of change in physical activity in COPD | Wrong intervention - no resistance training in intervention |
| 261 | Kato, 2020 | Effectiveness of lifestyle intervention using the internet of things system for individuals with early type 2 diabetes mellitus | Wrong intervention - no resistance training in intervention |
| 262 | Kattelmann, 2014 | The effects of Young Adults Eating and Active for Health (YEAH): a theory-based Web-delivered intervention | Wrong intervention - no resistance training in intervention |
| 263 | Kaur, 2024 | Implementing changing behaviour towards aerobic and strength exercise: Results of a randomised, phase I study determining the safety, feasibility, and consumer-evaluation of an online exercise program in persons with multiple sclerosis | Wrong intervention - not mHealth |
| 264 | Kawaguchi, 2024 | Effects of a Mobile App to Promote Social Participation on Older Adults: Randomized Controlled Trial | Wrong intervention - no resistance training in intervention |
| 265 | Kayser, 2727 | A web-based tailored nursing intervention (TAVIE en m@rche) aimed at increasing walking after an acute coronary syndrome: Multicentre randomized trial | Wrong intervention - no resistance training in intervention |
| 266 | Keadle, 2021 | Charity-based incentives motivate young adult cancer survivors to increase physical activity: a pilot randomized clinical trial | Wrong intervention - not mHealth |
| 267 | Kelders, 2010 | Usage and effect of a web-based intervention for the prevention of overweight; a RCT | Wrong intervention - resistance training not prescribed via mHealth |
| 268 | Kelders, 2011 | Effectiveness of a Web-based intervention aimed at healthy dietary and physical activity behavior: a randomized controlled trial about users and usage | Wrong intervention - no resistance training in intervention |
| 269 | Kelechi, 2020 | A Lower Leg Physical Activity Intervention for Individuals With Chronic Venous Leg Ulcers: Randomized Controlled Trial | Wrong intervention - no resistance training in intervention |
| 270 | Kenfield, 2019 | Feasibility, Acceptability, and Behavioral Outcomes from a Technology-enhanced Behavioral Change Intervention (Prostate 8): A Pilot Randomized Controlled Trial in Men with Prostate Cancer | Wrong intervention - no resistance training in intervention |
| 271 | Kermarrec, 2020 | EFFECT OF A TAILORED MOBILE APP ON PHYSICAL ACTIVITY IN HEALTHY ADULTS: A RANDOMIZED CONTROLLED TRIAL | Wrong intervention - no resistance training in intervention |
| 272 | Kernot, 2014 | Usability testing and piloting of the mums step it up program - A team-based social networking physical activity intervention for women with young children | Wrong intervention - no resistance training in intervention |
| 273 | Kernot, 2019 | Effectiveness of a Facebook-Delivered Physical Activity Intervention for Postpartum Women: A Randomized Controlled Trial | Wrong intervention - no resistance training in intervention |
| 274 | Keshavarz, 2023 | Online Circuit Training Increases Adherence to Physical Activity: A Randomized Controlled Trial of Men with Obesity | Wrong intervention - not mHealth |
| 275 | Khalili, 2022 | Health Within Reach-a Patient-Centered Intervention to Increase Hepatitis B Screening Among Asian Americans: a Randomized Clinical Trial | Wrong intervention - no resistance training in intervention |
| 276 | Khoshrounejad, 2022 | Effect of a Text Message-Based Support Program on Outcomes of Patients After Flexor Tendon Injury Repair | Wrong intervention - not mHealth |
| 277 | Khunti, 2021 | Behavioural interventions to promote physical activity in a multiethnic population at high risk of diabetes: PROPELS three-arm RCT | Wrong intervention - no resistance training in intervention |
| 278 | Khunti, 2021 | Promoting physical activity in a multi-ethnic population at high risk of diabetes: the 48-month PROPELS randomised controlled trial | Wrong intervention - no resistance training in intervention |
| 279 | Kim, 2015 | Effects of an internet-based lifestyle intervention on cardio-metabolic risks and stress in Korean workers with metabolic syndrome: A controlled trial | Wrong intervention - no resistance training in intervention |
| 280 | Kim, 2019 | Theeffectofasmartphone-based, patient-centered diabetes care system in patients with type 2 diabetes: A randomized, controlled trial for 24 weeks | Wrong intervention - no resistance training in intervention |
| 281 | Kim, 2021 | Effects of ICT-Based Multicomponent Program on Body Composition and Cognitive Function in Older Adults: A Randomized Controlled Clinical Study | Wrong intervention - not mHealth |
| 282 | Kim, 2023 | The Effectiveness of a Mobile Phone-Based Physical Activity Program for Treating Depression, Stress, Psychological Well-Being, and Quality of Life Among Adults: Quantitative Study | Wrong outcome |
| 283 | Kim, 2024 | A randomized controlled trial of an app-based intervention on physical activity and glycemic control in people with type 2 diabetes | Wrong intervention - not mHealth |
| 284 | Kim, 2024 | Effect of a smartphone-based physical intervention on depression, fitness factors and movement characteristics in adults | Wrong intervention - not mHealth |
| 285 | King, 1449 | Employing virtual advisors in preventive care for underserved communities: Results from the compass study | Wrong intervention - no resistance training in intervention |
| 286 | King, 2013 | Harnessing Different Motivational Frames via Mobile Phones to Promote Daily Physical Activity and Reduce Sedentary Behavior in Aging Adults | Wrong intervention - no resistance training in intervention |
| 287 | King, 2016 | Effects of three motivationally targeted mobile device applications on initial physical activity and sedentary behavior change in midlife and older adults: A randomized trial | Wrong intervention - no resistance training in intervention |
| 288 | Klaren, 2014 | Efficacy of a behavioral intervention for reducing sedentary behavior in persons with multiple sclerosis: A pilot examination | Wrong intervention - no resistance training in intervention |
| 289 | Knight, 2014 | Health promotion through primary care: Enhancing self-management with activity prescription and mhealth | Wrong intervention - not mHealth |
| 290 | Knight, 2014 | Prescribing physical activity for healthy aging: Longitudinal follow-up and mixed method analysis of a primary care intervention | Wrong intervention - no resistance training in intervention |
| 291 | Kolt, 2017 | Using Web 2.0 applications to promote health-related physical activity: Findings from the WALK 2.0 randomised controlled trial | Wrong intervention - no resistance training in intervention |
| 292 | Konstantinidis, 2016 | Design, Implementation, and Wide Pilot Deployment of FitForAll: An Easy to use Exergaming Platform Improving Physical Fitness and Life Quality of Senior Citizens | Wrong intervention - not mHealth |
| 293 | Kooiman, 2018 | Self-tracking of Physical Activity in People With Type 2 Diabetes: A Randomized Controlled Trial | Wrong outcome |
| 294 | Koppenaal, 2022 | The 3-Month Effectiveness of a Stratified Blended Physiotherapy Intervention in Patients With Nonspecific Low Back Pain: Cluster Randomized Controlled Trial | Wrong intervention - resistance training not prescribed via mHealth |
| 295 | Kravitz, 2020 | Feasibility, Acceptability, and Influence of mHealth-Supported N-of-1 Trials for Enhanced Cognitive and Emotional Well-Being in US Volunteers | Wrong intervention - no resistance training in intervention |
| 296 | Krebs, 2017 | An eHealth Intervention to Increase Physical Activity and Healthy Eating in Older Adult Cancer Survivors: Summative Evaluation Results | Wrong intervention - not mHealth |
| 297 | Kroesen, 2024 | A cardiac-rehab behaviour intervention to reduce sedentary time in coronary artery disease patients: the SIT LESS randomized controlled trial | Wrong intervention - not mHealth |
| 298 | Krzyzanowski, 2020 | Rams Have Heart, a Mobile App Tracking Activity and Fruit and Vegetable Consumption to Support the Cardiovascular Health of College Students: Development and Usability Study | Wrong intervention - no resistance training in intervention |
| 299 | Kwon, 2018 | An mhealth management platform for patients with chronic obstructive pulmonary disease (efil breath): Randomized controlled trial | Wrong outcome |
| 300 | Lachausse, 2012 | My student body: effects of an internet-based prevention program to decrease obesity among college students | Wrong intervention - no resistance training in intervention |
| 301 | Lambert, 2023 | Adding web-based support to exercise referral schemes improves symptoms of depression in people with elevated depressive symptoms: A secondary analysis of the e-coachER randomised controlled trial | Wrong intervention - no resistance training in intervention |
| 302 | Lancioni, 2020 | A smartphone-based intervention to enhance functional occupation and mood in people with neurodevelopmental disorders: A research extension | Wrong intervention - no resistance training in intervention |
| 303 | Landais, 2022 | Promoting an active choice among physically inactive adults: a randomised web-based four-arm experiment | Wrong intervention - no resistance training in intervention |
| 304 | Langlais, 2022 | Quality of Life of Prostate Cancer Survivors Participating in a Remotely Delivered Web-Based Behavioral Intervention Pilot Randomized Trial | Wrong intervention - no resistance training in intervention |
| 305 | Lao, 2024 | The Feasibility and Effects of Smartphone-Based Application on Cardiac Rehabilitation for Patients After Percutaneous Coronary Intervention: A Randomized Controlled Trial | Wrong intervention - no resistance training in intervention |
| 306 | Lawford, 2018 | Moderators of Effects of Internet-Delivered Exercise and Pain Coping Skills Training for People With Knee Osteoarthritis: Exploratory Analysis of the IMPACT Randomized Controlled Trial | Wrong intervention - not mHealth |
| 307 | Lawrason, 2023 | Evaluating the Feasibility, Acceptability, and Engagement of an mHealth Physical Activity Intervention for Adults With Spinal Cord Injury Who Walk: A Randomized Controlled Trial | Wrong intervention - no resistance training in intervention |
| 308 | Lee, 2017 | Effectiveness of an application-based neck exercise as a pain management tool for office workers with chronic neck pain and functional disability: A pilot randomized trial | Wrong intervention - no resistance training in intervention |
| 309 | Lee, 2018 | Mobile health, physical activity, and obesity: Subanalysis of a randomized controlled trial | Wrong intervention - no resistance training in intervention |
| 310 | Lee, 2019 | Changes in Weight and Health-Related Behavior Using Smartphone Applications in Patients With Colorectal Polyps | Wrong intervention - resistance training not prescribed via mHealth |
| 311 | Lee, 2022 | Effects of a Gamified, Behavior Change Technique-Based Mobile App on Increasing Physical Activity and Reducing Anxiety in Adults With Autism Spectrum Disorder: Feasibility Randomized Controlled Trial | Wrong intervention - no resistance training in intervention |
| 312 | Lee, 2023 | Effectiveness of a theory-driven mHealth intervention in promoting post-surgery rehabilitation adherence in patients who had anterior cruciate ligament reconstruction: A randomized clinical trial | Wrong outcome |
| 313 | Lee, 2024 | Effectiveness of Mobile-Based Progressive and Fixed Physical Activity on Depression, Stress, Anxiety, and Quality of Life Outcomes Among Adults in South Korea: Randomized Controlled Trial | Wrong outcome |
| 314 | Leinonen, 2017 | Feasibility of Gamified Mobile Service Aimed at Physical Activation in Young Men: Population-Based Randomized Controlled Study (MOPO) | Wrong population |
| 315 | LeMat, 2024 | Impact of a Self-Autonomous Evaluation Station and Personalized Training Algorithm on Quality of Life and Physical Capacities in Sedentary Adults: Randomized Controlled Trial | Wrong study design |
| 316 | Levin, 2022 | Tracking Valued and Avoidant Functions with Health Behaviors: A Randomized Controlled Trial of the Acceptance and Commitment Therapy Matrix Mobile App | Wrong intervention - no resistance training in intervention |
| 317 | Lewis, 2023 | Evaluating the feasibility and efficacy of a home-based combined high intensity interval and moderate intensity training program for increasing physical activity among low-active adults: A randomized pilot trial | Wrong outcome |
| 318 | Li, 2020 | Effects of a 12-Week Multifaceted Wearable-Based Program for People With Knee Osteoarthritis: Randomized Controlled Trial | Wrong intervention - no resistance training in intervention |
| 319 | Li, 2023 | Intelligent physical activity versus modified behavioral activation in adolescent and young adult cancer patients with psychological distress: A randomized, controlled pilot trial | Wrong intervention - no resistance training in intervention |
| 320 | Lieber, 1754 | A national interactive web-based physical activity intervention in women, evaluation of the American Heart Association choose to move program 2006-2007 | Wrong intervention - no resistance training in intervention |
| 321 | Lieffers, 2016 | A retrospective analysis of real-world use of the eaTracker My Goals website by adults from Ontario and Alberta, Canada | Wrong intervention - no resistance training in intervention |
| 322 | Lim, 2021 | Effect of a Smartphone App on Weight Change and Metabolic Outcomes in Asian Adults with Type 2 Diabetes: A Randomized Clinical Trial | Wrong intervention - resistance training not prescribed via mHealth |
| 323 | Linke, 2019 | Association Between Physical Activity Intervention Website Use and Physical Activity Levels Among Spanish-Speaking Latinas: Randomized Controlled Trial | Wrong intervention - no resistance training in intervention |
| 324 | Linnan, 2020 | Results of caring and reaching for health (CARE): a cluster-randomized controlled trial assessing a worksite wellness intervention for child care staff | Wrong intervention - no resistance training in intervention |
| 325 | Lison, 2020 | Impact of a Web-Based Exercise and Nutritional Education Intervention in Patients Who Are Obese with Hypertension: Randomized Wait-List Controlled Trial | Wrong intervention - no resistance training in intervention |
| 326 | Liu, 2022 | Effect of Mobile Internet Technology in Health Management of Heart Failure Patients Guiding Cardiac Rehabilitation | Wrong intervention - no resistance training in intervention |
| 327 | Louis Walthouwer, 2015 | Comparing a video and text version of a web-based computer-tailored intervention for obesity prevention: A randomized controlled trial | Wrong intervention - no resistance training in intervention |
| 328 | Lowensteyn, 2019 | The Sustainability of a Workplace Wellness Program That Incorporates Gamification Principles: Participant Engagement and Health Benefits After 2 Years | Wrong intervention - resistance training not prescribed via mHealth |
| 329 | Lugones-Sanchez, 2020 | Effectiveness of an mHealth Intervention Combining a Smartphone App and Smart Band on Body Composition in an Overweight and Obese Population: Randomized Controlled Trial (EVIDENT 3 Study) | Wrong intervention - no resistance training in intervention |
| 330 | Lugones-Sanchez, 2022 | Long-term Effectiveness of a Smartphone App Combined With a Smart Band on Weight Loss, Physical Activity, and Caloric Intake in a Population With Overweight and Obesity (Evident 3 Study): Randomized Controlled Trial | Wrong intervention - no resistance training in intervention |
| 331 | Lugtrek, 2021 | A Personal Health System for Self-Management of Congestive Heart Failure (HeartMan): Development, Technical Evaluation, and Proof-of-Concept Randomized Controlled Trial | Wrong intervention - not mHealth |
| 332 | Lunde, 2020 | Long-term follow-up with a smartphone application improves exercise capacity post cardiac rehabilitation: A randomized controlled trial | Wrong intervention - no resistance training in intervention |
| 333 | Lynch, 2016 | Tablet-Aided BehavioraL intervention EffecT on Self-management skills (TABLETS) for Diabetes | Wrong intervention - no resistance training in intervention |
| 334 | Ma, 2013 | Translating the diabetes prevention program lifestyle intervention for weight loss into primary care: A randomized trial | Wrong intervention - not mHealth |
| 335 | MacPherson, 2019 | Effects of Mobile Health Prompts on Self-Monitoring and Exercise Behaviors Following a Diabetes Prevention Program: Secondary Analysis From a Randomized Controlled Trial | Wrong intervention - no resistance training in intervention |
| 336 | Maddison, 2015 | A mobile phone intervention increases physical activity in people with cardiovascular disease: Results from the HEART randomized controlled trial | Wrong intervention - no resistance training in intervention |
| 337 | Maddison, 2019 | Effects and costs of real-time cardiac telerehabilitation: Randomised controlled non-inferiority trial | Wrong intervention - not mHealth |
| 338 | Madrona Marcos, 2019 | Effect of a motivational physical activity program on lipid parameters in patients with obesity and overweight | Not in English |
| 339 | Magoc, 2011 | Using the web to increase physical activity in college students | Wrong intervention - no resistance training in intervention |
| 340 | Maher, 2015 | A Web-Based, Social Networking Physical Activity Intervention for Insufficiently Active Adults Delivered via Facebook App: Randomized Controlled Trial | Wrong intervention - no resistance training in intervention |
| 341 | Mailey, 2010 | Internet-delivered physical activity intervention for college students with mental health disorders: a randomized pilot trial | Wrong intervention - no resistance training in intervention |
| 342 | Mailey, 2019 | InDependent but not Alone: A Web-Based Intervention to Promote Physical and Mental Health among Military Spouses | Wrong intervention - no resistance training in intervention |
| 343 | Mainsbridge, 2014 | The effect of an e-health intervention designed to reduce prolonged occupational sitting on mean arterial pressure | Wrong intervention - no resistance training in intervention |
| 344 | Maloney, 2011 | Effectiveness of Web-Based Versus Face-To-Face Delivery of Education in Prescription of Falls-Prevention Exercise to Health Professionals: Randomized Trial | Wrong intervention - no resistance training in intervention |
| 345 | Mamede, 2021 | Combining web-based gamification and physical nudges with an app (MoveMore) to promote walking breaks and reduce sedentary behavior of office workers: Field study | Wrong intervention - no resistance training in intervention |
| 346 | Mandic, 2020 | Successful promotion of physical activity among students of medicine through motivational interview and Web-based intervention | Wrong intervention - no resistance training in intervention |
| 347 | Manzoor, 2021 | Effectiveness of mobile health augmented cardiac rehabilitation on behavioural outcomes among post-acute coronary syndrome patients: A randomised controlled trial | Wrong intervention - no resistance training in intervention |
| 348 | Marcus, 2016 | Pasos Hacia La Salud: A randomized controlled trial of an internet-delivered physical activity intervention for Latinas | Wrong intervention - no resistance training in intervention |
| 349 | Marsaux, 2015 | Effects of a web-based personalized intervention on physical activity in European adults:a randomized controlled trial | Wrong intervention - no resistance training in intervention |
| 350 | Martin, 2015 | mActive: A randomized clinical trial of an automated mHealth intervention for physical activity promotion | Wrong intervention - no resistance training in intervention |
| 351 | Martinez-Rodriguez, 2022 | New App-Based Dietary and Lifestyle Intervention on Weight Loss and Cardiovascular Health | Wrong intervention - no resistance training in intervention |
| 352 | Maruyama, 2010 | Effect of a worksite-based intervention program on metabolic parameters in middle-aged male white-collar workers: A randomized controlled trial | Wrong intervention - no resistance training in intervention |
| 353 | Masato, 2017 | A pilot lifestyle intervention study: effects of an intervention using an activity monitor and Twitter on physical activity and body composition | Wrong intervention - no resistance training in intervention |
| 354 | Mascarenhas, 2018 | Increasing Physical Activity in Mothers Using Video Exercise Groups and Exercise Mobile Apps: Randomized Controlled Trial | Wrong intervention - no resistance training in intervention |
| 355 | Massoudi, 2010 | A web-based intervention to support increased physical activity among at-risk adults | Wrong study design |
| 356 | Mateo, 2014 | Access to a behavioral weight loss website with or without group sessions increased weight loss in statewide campaign | Wrong intervention - no resistance training in intervention |
| 357 | Mattila, 2013 | Personal Health Technologies in Employee Health Promotion: Usage Activity, Usefulness, and Health-Related Outcomes in a 1-Year Randomized Controlled Trial | Wrong intervention - no resistance training in intervention |
| 358 | Mattila, 2022 | Evaluation of the Immediate Effects of Web-Based Intervention Modules for Goals, Planning, and Coping Planning on Physical Activity: Secondary Analysis of a Randomized Controlled Trial on Weight Loss Maintenance | Wrong intervention - no resistance training in intervention |
| 359 | Mattila, 2022 | Users' Experiences With the NoHoW Web-Based Toolkit With Weight and Activity Tracking in Weight Loss Maintenance: Long-term Randomized Controlled Trial | Wrong intervention - no resistance training in intervention |
| 360 | Matz-Costa, 2018 | A Pilot Randomized Trial of an Intervention to Enhance the Health-Promoting Effects of Older Adults' Activity Portfolios: The Engaged4Life Program | Wrong intervention - no resistance training in intervention |
| 361 | Maurer, 2018 | A randomized study to evaluate the effect of exercise on fatigue in people with relapsing-remitting multiple sclerosis treated with fingolimod | Wrong outcome |
| 362 | Mayer, 2017 | SurvivorCHESS to increase physical activity in colon cancer survivors: can we get them moving? | Wrong intervention - no resistance training in intervention |
| 363 | Mazuz, 2020 | Developing Self-Management Application of Fall Prevention Among Older Adults: A Content and Usability Evaluation | Wrong intervention - resistance training not prescribed via mHealth |
| 364 | McCormack, 2022 | Effectiveness of an 8-Week Physical Activity Intervention Involving Wearable Activity Trackers and an eHealth App: Mixed Methods Study | Wrong intervention - no resistance training in intervention |
| 365 | Mehra, 2020 | Evaluation of a blended physical activity intervention for older adults: Mixed methods study | Wrong study design |
| 366 | Melchart, 2018 | The TALENT II study: a randomized controlled trial assessing the impact of an individual health management (IHM) on stress reduction | Wrong intervention - no resistance training in intervention |
| 367 | Melton, 2015 | Evaluating a Physical Activity App in the Classroom: A Mixed Methodological Approach Among University Students | Wrong study design |
| 368 | Memon, 2018 | The effectiveness of an incentivized physical activity programme (Active student) among female medical students in Pakistan: A randomized controlled trial | Wrong intervention - no resistance training in intervention |
| 369 | Mendoza-Vasconez, 2022 | Regular and App-Enhanced Maintenance of Physical Activity among Latinas: A Feasibility Study | Wrong intervention - no resistance training in intervention |
| 370 | Mhurchu, 2019 | A co-designed mHealth programme to support healthy lifestyles in Maori and Pasifika peoples in New Zealand (OL@-OR@): a cluster-randomised controlled trial | Wrong intervention - no resistance training in intervention |
| 371 | Michelsen, 2022 | Effect of a Lifestyle-Focused Web-Based Application on Risk Factor Management in Patients Who Have Had a Myocardial Infarction: Randomized Controlled Trial | Wrong intervention - no resistance training in intervention |
| 372 | Miller, 2021 | Feasibility of resp-fit: Technology-enhanced self-management intervention for adults with copd | Wrong intervention - not mHealth |
| 373 | Millstein, 2020 | An Exploration of Health Behaviors in a Mind-Body Resilience Intervention for Parents of Children with Developmental Disabilities | Wrong population |
| 374 | MinnsLowe, 2020 | "WALK30X5": a feasibility study of a physiotherapy walking programme for people with mild to moderate musculoskeletal conditions | Wrong intervention - no resistance training in intervention |
| 375 | Miragall, 2018 | Increasing physical activity through an Internet-based motivational intervention supported by pedometers in a sample of sedentary students: A randomised controlled trial | Wrong intervention - no resistance training in intervention |
| 376 | Miyazato, 2021 | Effect of a mobile digital intervention to enhance physical activity in individuals with metabolic disorders on voiding patterns measured by 24-h voided volume monitoring system: Kumejima Digital Health Project (KDHP) | Wrong intervention - no resistance training in intervention |
| 377 | Mizuta, 2024 | Effectiveness of Metaverse Space-Based Exercise Video Distribution in Young Adults: Randomized Controlled Trial | Wrong outcome |
| 378 | Mo, 2019 | Using gamification and social incentives to increase physical activity and related social cognition among undergraduate students in Shanghai, China | Wrong intervention - no resistance training in intervention |
| 379 | Monninghoff, 2022 | The Effect of a Future-Self Avatar Mobile Health Intervention (FutureMe) on Physical Activity and Food Purchases: Randomized Controlled Trial | Wrong intervention - no resistance training in intervention |
| 380 | Monroe, 2023 | Harnessing technology and gamification to increase adult physical activity: a cluster randomized controlled trial of the Columbia Moves pilot | Wrong intervention - no resistance training in intervention |
| 381 | Mora-Gonzalez, 2020 | The "$in TIME" Gamification Project: Using a Mobile App to Improve Cardiorespiratory Fitness Levels of College Students | Wrong intervention - no resistance training in intervention |
| 382 | Mora-Gonzalez, 2022 | "STAR WARSTM: The First Jedi" Gamification Program: Improvement of Fitness Among College Students | Wrong intervention - no resistance training in intervention |
| 383 | Moraitis, 2023 | Feasibility and acceptability of an mHealth, home-based exercise intervention in colorectal cancer survivors: A pilot randomized controlled trial | Wrong intervention - no resistance training in intervention |
| 384 | Moreau, 2015 | Development of a Fully Automated, Web-Based, Tailored Intervention Promoting Regular Physical Activity Among Insufficiently Active Adults With Type 2 Diabetes: Integrating the I-Change Model, Self-Determination Theory, and Motivational Interviewing Components | Wrong intervention - no resistance training in intervention |
| 385 | Morgan, 2011 | Efficacy of a workplace-based weight loss program for overweight male shift workers: The Workplace POWER (Preventing Obesity Without Eating like a Rabbit) randomized controlled trial | Wrong intervention - no resistance training in intervention |
| 386 | Morgan, 2013 | The SHED-IT community trial: a randomized controlled trial of internet- and paper-based weight loss programs tailored for overweight and obese men | Wrong intervention - no resistance training in intervention |
| 387 | Morrison, 2016 | Findings from a pilot Randomised trial of an Asthma Internet Self-management Intervention (RAISIN) | Wrong intervention - no resistance training in intervention |
| 388 | Motl, 2011 | Internet intervention for increasing physical activity in persons with multiple sclerosis | Wrong intervention - no resistance training in intervention |
| 389 | Motl, 2017 | Randomized controlled trial of an e-learning designed behavioral intervention for increasing physical activity behavior in multiple sclerosis | Wrong intervention - not mHealth |
| 390 | Moy, 2015 | An internet-mediated pedometer-based program improves health-related quality-of-life domains and daily step counts in COPD: A randomized controlled trial | Wrong intervention - no resistance training in intervention |
| 391 | Moy, 2016 | Long-Term Effects of an Internet-Mediated Pedometer-Based Walking Program for Chronic Obstructive Pulmonary Disease: Randomized Controlled Trial | Wrong intervention - no resistance training in intervention |
| 392 | Mueller, 2023 | Supporting Weight Management during COVID-19 (SWiM-C): twelve-month follow-up of a randomised controlled trial of a web-based, ACT-based, guided self-help intervention | Wrong intervention - no resistance training in intervention |
| 393 | Muellmann, 2019 | Effects of two web-based interventions promoting physical activity among older adults compared to a delayed intervention control group in Northwestern Germany: Results of the PROMOTE community-based intervention trial | Wrong intervention - not mHealth |
| 394 | Muller, 2017 | Effects on Engagement and Health Literacy Outcomes of Web-Based Materials Promoting Physical Activity in People With Diabetes: An International Randomized Trial | Wrong intervention - no resistance training in intervention |
| 395 | Muntaner-Mas, 2017 | Effects of a Whatsapp-delivered physical activity intervention to enhance health-related physical fitness components and cardiovascular disease risk factors in older adults | Wrong study design |
| 396 | Muralidharan, 2019 | Engagement and Weight Loss: Results from the Mobile Health and Diabetes Trial | Wrong intervention - no resistance training in intervention |
| 397 | Murphy, 2021 | Intensive and app-delivered occupational therapy to improve upper extremity function in early diffuse cutaneous systemic sclerosis: a pilot two-arm trial | Wrong intervention - no resistance training in intervention |
| 398 | Murray, 2013 | Health on the web: randomised trial of work-based online screening and brief intervention for hazardous and harmful drinking | Wrong intervention - no resistance training in intervention |
| 399 | Murray, 2022 | The Impact of Web-Based Physical Activity Interventions on Depression and Anxiety Among College Students: Randomized Experimental Trial | Wrong intervention - not mHealth |
| 400 | Muzquiz-Barbera, 2023 | "Own doctor" presence in a web-based lifestyle intervention for adults with obesity and hypertension: A randomized controlled trial | Wrong intervention - no resistance training in intervention |
| 401 | Myers, 2020 | Effectiveness of the Fun For Wellness Online Behavioral Intervention to Promote Subjective Well-Being in Adults with Obesity: A Randomized Controlled Trial | Wrong intervention - no resistance training in intervention |
| 402 | Myers, 2020 | Effectiveness of the Fun for Wellness Web-Based Behavioral Intervention to Promote Physical Activity in Adults With Obesity (or Overweight): Randomized Controlled Trial | Wrong intervention - no resistance training in intervention |
| 403 | Naami Nazari, 2020 | The effect of web-based educational intervention on physical activity-related energy expenditure among middle-aged women with overweight and obesity: An application of social cognitive theory | Wrong intervention - no resistance training in intervention |
| 404 | Nahm, 2020 | Effects of mobile-based exercise intervention on health indices by the comparison of personal training time in male workers | Wrong study design |
| 405 | Naimark, 2015 | The impact of a Web-based app (eBalance) in promoting healthy lifestyles: Randomized controlled trial | Wrong intervention - no resistance training in intervention |
| 406 | Nakata, 2019 | Web-based intervention to promote weight-loss maintenance using an activity monitor: A randomized controlled trial | Wrong intervention - no resistance training in intervention |
| 407 | Nam, 2020 | Effects of a social-media-based support on premenstrual syndrome and physical activity among female university students in South Korea | Wrong intervention - no resistance training in intervention |
| 408 | Namli Seker, 2024 | Comparison of the Effects of Two Different Exercise Programs on Lower Limb Functions, Posture, and Physical Activity in Office Workers Working at Home and in Office Alternately: A Randomized Controlled Trial | Wrong intervention - not mHealth |
| 409 | Napolitano, 2021 | Feasibility of a digital intervention to promote healthy weight management among postpartum african american/black women | Wrong intervention - no resistance training in intervention |
| 410 | Naslund, 2016 | Wearable devices and mobile technologies for supporting behavioral weight loss among people with serious mental illness | Wrong intervention - resistance training not prescribed via mHealth |
| 411 | Navarro, 2020 | Manipulating self-avatar body dimensions in virtual worlds to complement an internet-delivered intervention to increase physical activity in overweight women | Wrong intervention - no resistance training in intervention |
| 412 | Neil-Sztramko, 2017 | Feasibility of a telephone and web-based physical activity intervention for women shift workers | Wrong intervention - no resistance training in intervention |
| 413 | Nguyen, 2013 | Internet-Based Dyspnea Self-Management Support for Patients With Chronic Obstructive Pulmonary Disease | Wrong intervention - no resistance training in intervention |
| 414 | Nicklas, 2024 | The Fit After Baby randomized controlled trial: An mHealth postpartum lifestyle intervention for women with elevated cardiometabolic risk | Wrong intervention - no resistance training in intervention |
| 415 | Nikitina, 2018 | Feasibility of Virtual Tablet-Based Group Exercise Among Older Adults in Siberia: Findings From Two Pilot Trials | Wrong study design |
| 416 | Noormohammadpour, 2021 | Effectiveness of an individualized internet-based physical activity program: A randomized controlled trial | Wrong outcome |
| 417 | Nyberg, 2019 | Can the COPD web be used to promote self-management in patients with COPD in swedish primary care: a controlled pragmatic pilot trial with 3 month- and 12 month follow-up | Wrong intervention - no resistance training in intervention |
| 418 | Nystrom, 2017 | Behavioral activation versus physical activity via the internet: A randomized controlled trial | Wrong intervention - no resistance training in intervention |
| 419 | Oba, 2022 | Evaluating the feasibility of a remote-based training program supported by information and communications technology in the older adults living at home | Wrong intervention - not mHealth |
| 420 | O'Donnell, 2014 | The effect of goal setting on fruit and vegetable consumption and physical activity level in a Web-based intervention | Wrong intervention - no resistance training in intervention |
| 421 | Okazaki, 2014 | One-year outcome of an interactive internet-based physical activity intervention among university students | Wrong intervention - no resistance training in intervention |
| 422 | Olson, 2018 | The effectiveness of an online intervention in preventing excessive gestational weight gain: The e-moms roc randomized controlled trial | Wrong intervention - no resistance training in intervention |
| 423 | Orme, 2018 | Findings of the chronic obstructive pulmonary disease-sitting and exacerbations trial (COPD-SEAT) in reducing sedentary time using wearable and mobile technologies with educational support: Randomized controlled feasibility trial | Wrong intervention - no resistance training in intervention |
| 424 | Ormel, 2018 | Self-monitoring physical activity with a smartphone application in cancer patients: a randomized feasibility study (SMART-trial) | Wrong intervention - no resistance training in intervention |
| 425 | Ostlind, 2021 | Physical activity patterns, adherence to using a wearable activity tracker during a 12-week period and correlation between self-reported function and physical activity in working age individuals with hip and/or knee osteoarthritis | Wrong intervention - no resistance training in intervention |
| 426 | Paldan, 2021 | Supervised Exercise Therapy Using Mobile Health Technology in Patients With Peripheral Arterial Disease: Pilot Randomized Controlled Trial | Wrong intervention - resistance training not prescribed via mHealth |
| 427 | Park, 2020 | Evaluating the effect of a smartphone app-based self-management program for people with COPD: A randomized controlled trial | Wrong outcome |
| 428 | Park, 2021 | Mobile Health Intervention Promoting Physical Activity in Adults Post Cardiac Rehabilitation: Pilot Randomized Controlled Trial | Wrong intervention - no resistance training in intervention |
| 429 | Partridge, 2015 | Effectiveness of a mHealth Lifestyle Program With Telephone Support (TXT2BFiT) to Prevent Unhealthy Weight Gain in Young Adults: Randomized Controlled Trial | Wrong intervention - no resistance training in intervention |
| 430 | Pas, 2020 | Effectiveness of an e-health tennis-specific injury prevention programme: randomised controlled trial in adult recreational tennis players | Wrong outcome |
| 431 | Patnaik, 2022 | Effectiveness of mobile application for promotion of physical activity among newly diagnosed patients of type II diabetes -A randomized controlled trial | Wrong intervention - no resistance training in intervention |
| 432 | Paul, 2016 | Increasing physical activity in stroke survivors using STARFISH, an interactive mobile phone application: A pilot study | Wrong intervention - no resistance training in intervention |
| 433 | Paul, 2019 | Web-based physiotherapy for people affected by multiple sclerosis: a single blind, randomized controlled feasibility study | Wrong outcome |
| 434 | Peacock, 2020 | Effect of novel technology-enabled multidimensional physical activity feedback in primary care patients at risk of chronic disease - The MIPACT study: A randomised controlled trial | Wrong intervention - resistance training not prescribed via mHealth |
| 435 | Pedersen, 2014 | An e-health intervention designed to increase workday energy expenditure by reducing prolonged occupational sitting habits | Wrong intervention - not mHealth |
| 436 | Peels, 2014 | Long-term health outcomes and cost-effectiveness of a computer-tailored physical activity intervention among people aged over fifty: Modelling the results of a randomized controlled trial | Wrong intervention - no resistance training in intervention |
| 437 | Peels, 2014 | The differentiated effectiveness of a printed versus a Web-based tailored physical activity intervention among adults aged over 50 | Wrong intervention - no resistance training in intervention |
| 438 | Pei-Jung, 2021 | Long-term effectiveness of an mHealth-tailored physical activity intervention in youth with congenital heart disease: a randomized controlled trial | Wrong intervention - no resistance training in intervention |
| 439 | Pekmezi, 2010 | Feasibility of using computer-tailored and internet-based interventions to promote physical activity in underserved populations | Wrong intervention - no resistance training in intervention |
| 440 | Pellegrini, 2012 | The comparison of a technology-based system and an in-person behavioral weight loss intervention | Wrong intervention - no resistance training in intervention |
| 441 | Perera, 2023 | Social support for exercise from pregnancy to postpartum and the potential impact of a mobile application: A randomized control pilot trial in Southern United States | Wrong intervention - no resistance training in intervention |
| 442 | Perez-Cruzado, 2013 | Improving Adherence Physical Activity with a Smartphone Application Based on Adults with Intellectual Disabilities (APPCOID) | Wrong intervention - resistance training not prescribed via mHealth |
| 443 | Perez-Lopez, 2022 | "STAR WARS: The first Jedi" Gamification Program: Use of a Mobile App to Improve Body Composition in College Students | Wrong intervention - no resistance training in intervention |
| 444 | Petrella, 2014 | Mobile health, exercise and metabolic risk: a randomized controlled trial | Wrong outcome |
| 445 | Petrella, 2017 | Hockey Fans in Training: A Pilot Pragmatic Randomized Controlled Trial | Wrong intervention - no resistance training in intervention |
| 446 | PfaeffliDale, 2015 | Acceptability of a mobile health exercise-based cardiac rehabilitation intervention: A randomized trial | Wrong study design |
| 447 | Phillips, 2022 | Optimization of a technology-supported physical activity promotion intervention for breast cancer survivors: Results from Fit2Thrive | Wrong intervention - no resistance training in intervention |
| 448 | Piao, 2020 | Use of the Healthy Lifestyle Coaching Chatbot App to Promote Stair-Climbing Habits Among Office Workers: Exploratory Randomized Controlled Trial | Wrong intervention - no resistance training in intervention |
| 449 | Pilutti, 2014 | Randomized controlled trial of a behavioral intervention targeting symptoms and physical activity in multiple sclerosis | Wrong intervention - no resistance training in intervention |
| 450 | Pilutti, 2014 | Internet-delivered lifestyle physical activity intervention improves body composition in multiple sclerosis: Preliminary evidence from a randomized controlled trial | Wrong intervention - no resistance training in intervention |
| 451 | Plaete, 2015 | Acceptability, feasibility and effectiveness of an eHealth behaviour intervention using self-regulation: 'MyPlan' | Wrong intervention - no resistance training in intervention |
| 452 | Plotnikoff, 2017 | Integrating smartphone technology, social support and the outdoor physical environment to improve fitness among adults at risk of, or diagnosed with, Type 2 Diabetes: Findings from the 'eCoFit' randomized controlled trial | Wrong intervention - no resistance training in intervention |
| 453 | Plow, 2017 | Using mHealth Technology in a Self-Management Intervention to Promote Physical Activity Among Adults With Chronic Disabling Conditions: Randomized Controlled Trial | Wrong intervention - resistance training not prescribed via mHealth |
| 454 | Poirier, 2016 | Effectiveness of an activity tracker- and internet-based adaptive walking program for adults: A randomized controlled trial | Wrong intervention - no resistance training in intervention |
| 455 | Pomkai, 2024 | Digital Group-Based Intervention for Physical Activity Promotion Among Thai Adults During the COVID-19 Lockdown: Randomized Controlled Trial | Wrong intervention - no resistance training in intervention |
| 456 | Pope, 2022 | Feasibility of smartphone application- and social media-based intervention on college students' health outcomes: A pilot randomized trial | Wrong intervention - no resistance training in intervention |
| 457 | Poppe, 2018 | How Users Experience and Use an eHealth Intervention Based on Self-Regulation: Mixed-Methods Study | Wrong intervention - no resistance training in intervention |
| 458 | Poppe, 2019 | Efficacy of a Self-Regulation-Based Electronic and Mobile Health Intervention Targeting an Active Lifestyle in Adults Having Type 2 Diabetes and in Adults Aged 50 Years or Older: Two Randomized Controlled Trials | Wrong intervention - no resistance training in intervention |
| 459 | Potzel, 2022 | A novel smartphone app to change risk behaviors of women after gestational diabetes: A randomized controlled trial | Wrong intervention - no resistance training in intervention |
| 460 | Puterman, 2022 | COVID-19 Pandemic and Exercise (COPE) trial: a multigroup pragmatic randomised controlled trial examining effects of app-based at-home exercise programs on depressive symptoms | Wrong outcome |
| 461 | Quinonez, 2016 | MHealth or eHealth? Efficacy, use, and appreciation of a web-based computer-tailored physical activity intervention for Dutch adults: A randomized controlled trial | Wrong intervention - no resistance training in intervention |
| 462 | Quintiliani, 2010 | Results of a Randomized Trial Testing Messages Tailored to Participant-Selected Topics Among Female College Students: Physical Activity Outcomes | Wrong intervention - no resistance training in intervention |
| 463 | Quintiliani, 2021 | Community health worker-delivered weight management intervention among public housing residents: A feasibility study | Wrong intervention - no resistance training in intervention |
| 464 | Rabbi, 2015 | Automated Personalized Feedback for Physical Activity and Dietary Behavior Change With Mobile Phones: A Randomized Controlled Trial on Adults | Wrong intervention - no resistance training in intervention |
| 465 | Rabin, 2012 | Internet-based physical activity intervention targeting young adult cancer survivors | Wrong intervention - no resistance training in intervention |
| 466 | Rafiq, 2023 | Effectiveness of lower limb rehabilitation protocol using mobile health on quality of life, functional strength, and functional capacity among knee osteoarthritis patients who are overweight and obese: A randomized-controlled trial | Wrong intervention - not mHealth |
| 467 | Raiszadeh, 2021 | In-Clinic Versus Web-Based Multidisciplinary Exercise-Based Rehabilitation for Treatment of Low Back Pain: Prospective Clinical Trial in an Integrated Practice Unit Model | Wrong study design |
| 468 | Ranjani, 2025 | Effectiveness of Mobile Health Applications for Cardiometabolic Risk Reduction in Urban and Rural India: A Pilot, Randomized Controlled Study | Wrong intervention - no resistance training in intervention |
| 469 | Rasera, 2022 | An Early Phase Trial Testing the Proof of Concept for a Gamified Smartphone App in Manipulating Automatic Evaluations of Exercise | Wrong intervention - no resistance training in intervention |
| 470 | Ratz, 2019 | Effects of Two Web-Based Interventions and Mediating Mechanisms on Stage of Change Regarding Physical Activity in Older Adults | Wrong intervention - no resistance training in intervention |
| 471 | Rayward, 2020 | Efficacy of an m-Health Physical Activity and Sleep Intervention to Improve Sleep Quality in Middle-Aged Adults: The Refresh Study Randomized Controlled Trial | Wrong intervention - no resistance training in intervention |
| 472 | Rebar, 2016 | Healthy mind, healthy body: A randomized trial testing the efficacy of a computer-tailored vs. interactive web-based intervention for increasing physical activity and reducing depressive symptoms | Wrong intervention - no resistance training in intervention |
| 473 | Recio-Rodriguez, 2016 | Short-term effectiveness of a mobile phone app for increasing physical activity and adherence to the mediterranean diet in primary care: A randomized controlled trial (EVIDENT II study) | Wrong intervention - no resistance training in intervention |
| 474 | Reed, 2018 | The impact of web-based feedback on physical activity and cardiovascular health of nurses working in a cardiovascular setting: A randomized trial | Wrong intervention - no resistance training in intervention |
| 475 | Reid, 2012 | Randomized trial of an internet-based computer-tailored expert system for physical activity in patients with heart disease | Wrong intervention - no resistance training in intervention |
| 476 | Robinson, 1082 | A web-based physical activity intervention benefits persons with low self-efficacy in COPD: results from a randomized controlled trial | Wrong intervention - no resistance training in intervention |
| 477 | Robinson, 2021 | A randomised trial of a web-based physical activity self-management intervention in COPD | Wrong intervention - resistance training not prescribed via mHealth |
| 478 | Robroek, 2012 | Initial and Sustained Participation in an Internet-delivered Long-term Worksite Health Promotion Program on Physical Activity and Nutrition | Wrong intervention - no resistance training in intervention |
| 479 | Rodriguez Sanchez-Laulhe, 2022 | An Exercise and Educational and Self-management Program Delivered With a Smartphone App (CareHand) in Adults With Rheumatoid Arthritis of the Hands: Randomized Controlled Trial | Wrong intervention - no resistance training in intervention |
| 480 | Rodriguez Sanchez-Laulhe, 2023 | The effects of a mobile app-delivered intervention in people with symptomatic hand osteoarthritis: a pragmatic randomized controlled trial | Wrong intervention - no resistance training in intervention |
| 481 | Rogers, 2016 | Applying a technology-based system for weight loss in adults with obesity | Wrong intervention - no resistance training in intervention |
| 482 | Rosas, 2020 | Effect of a Culturally Adapted Behavioral Intervention for Latino Adults on Weight Loss over 2 Years: A Randomized Clinical Trial | Wrong intervention - no resistance training in intervention |
| 483 | Rospo, 2016 | Cardiorespiratory Improvements Achieved by American College of Sports Medicine's Exercise Prescription Implemented on a Mobile App | Wrong intervention - no resistance training in intervention |
| 484 | Ross, 2016 | Impact of newer self-monitoring technology and brief phone-based intervention on weight loss: A randomized pilot study | Wrong intervention - no resistance training in intervention |
| 485 | Rovniak, 2016 | Engineering Online and In-Person Social Networks for Physical Activity: A Randomized Trial | Wrong intervention - no resistance training in intervention |
| 486 | Ruggiero, 2014 | Diabetes Island: Preliminary Impact of a Virtual World Self-Care Educational Intervention for African Americans With Type 2 Diabetes | Wrong intervention - no resistance training in intervention |
| 487 | Sabooteh, 2020 | Effect of web-based and software-based educational intervention on stages of behavior change of students' physical activity | Wrong intervention - resistance training not prescribed via mHealth |
| 488 | Saitoh, 2022 | Remote Cardiac Rehabilitation in Older Cardiac Disease: A Randomized Case Series Feasibility Study | Wrong intervention - not mHealth |
| 489 | Salaffi, 2015 | Web/Internet-based telemonitoring of a randomized controlled trial evaluating the time-integrated effects of a 24-week multicomponent intervention on key health outcomes in patients with fibromyalgia | Wrong intervention - not mHealth |
| 490 | Salas-Groves, 2024 | The Effect of Web-Based Culinary Medicine to Enhance Protein Intake on Muscle Quality in Older Adults: Randomized Controlled Trial | Wrong intervention - no resistance training in intervention |
| 491 | Saleh, 2023 | Effect of a Home-Based Mobile Health App Intervention on Physical Activity Levels in Patients With Heart Failure: A Randomized Controlled Trial | Wrong intervention - no resistance training in intervention |
| 492 | Salisbury, 2016 | Telehealth for patients at high risk of cardiovascular disease: pragmatic randomised controlled trial | Wrong intervention - no resistance training in intervention |
| 493 | Samaan, 2013 | South Asian Heart Risk Assessment (SAHARA): Randomized Controlled Trial Design and Pilot Study | Wrong intervention - no resistance training in intervention |
| 494 | Samendinger, 2018 | Testing group dynamics with a virtual partner to increase physical activity motivation | Wrong intervention - no resistance training in intervention |
| 495 | Sandal, 2021 | Effectiveness of App-Delivered, Tailored Self-management Support for Adults With Lower Back Pain-Related Disability A SELFBACK Randomized Clinical Trial | Wrong outcome |
| 496 | Sandroff, 2014 | Randomized controlled trial of physical activity, cognition, and walking in multiple sclerosis | Wrong intervention - no resistance training in intervention |
| 497 | Saran, 2018 | Follow-up monitoring of physical activity after rehabilitation by means of a mobile application: Effectiveness of measurements in different age groups | Wrong intervention - no resistance training in intervention |
| 498 | Saslow, 2017 | An Online Intervention Comparing a Very Low-Carbohydrate Ketogenic Diet and Lifestyle Recommendations Versus a Plate Method Diet in Overweight Individuals With Type 2 Diabetes: A Randomized Controlled Trial | Wrong intervention - resistance training not prescribed via mHealth |
| 499 | Schaller, 2016 | Promoting physical activity in low back pain patients: six months follow-up of a randomised controlled trial comparing a multicomponent intervention with a low intensity intervention | Wrong intervention - no resistance training in intervention |
| 500 | Schaller, 2017 | Effectiveness of a theory-based multicomponent intervention (Movement Coaching) on the promotion of total and domain-specific physical activity: a randomised controlled trial in low back pain patients | Wrong intervention - no resistance training in intervention |
| 501 | Schneider, 2012 | The influence of user characteristics and a periodic email prompt on exposure to an internet-delivered computer-tailored lifestyle program | Wrong intervention - no resistance training in intervention |
| 502 | Schneider, 2020 | Efficacy of internet-delivered cognitive behavioural therapy following an acute coronary event: A randomized controlled trial | Wrong intervention - no resistance training in intervention |
| 503 | Schoeppe, 2022 | Acceptability, usefulness, and satisfaction with a web-based video-tailored physical activity intervention: The TaylorActive randomized controlled trial | Wrong intervention - no resistance training in intervention |
| 504 | Schroe, 2020 | Which behaviour change techniques are effective to promote physical activity and reduce sedentary behaviour in adults: A factorial randomized trial of an e- A nd m-health intervention | Wrong intervention - no resistance training in intervention |
| 505 | Schulz, 2014 | Effects of a web-based tailored multiple-lifestyle intervention for adults: a two-year randomized controlled trial comparing sequential and simultaneous delivery modes | Wrong intervention - resistance training not prescribed via mHealth |
| 506 | Schweier, 2014 | A web-based peer-modeling intervention aimed at lifestyle changes in patients with coronary heart disease and chronic back pain: sequential controlled trial | Wrong intervention - no resistance training in intervention |
| 507 | Seekaew, 2015 | Encouraging use of the MyFitnessPal app does not lead to weight loss in primary care patients | Wrong intervention - no resistance training in intervention |
| 508 | Seguin-Fowler, 2020 | Web-Based Dissemination of a Civic Engagement Curriculum to Promote Healthy Eating and Active Living in Rural Towns: The eHEART Study | Wrong intervention - no resistance training in intervention |
| 509 | Seib, 2022 | Improving health-related quality of life in women with breast, blood, and gynaecological Cancer with an eHealth-enabled 12-week lifestyle intervention: the women's wellness after Cancer program randomised controlled trial | Wrong intervention - no resistance training in intervention |
| 510 | Seker, 2024 | Comparison of the Effects of Two Different Exercise Programs on Lower Limb Functions, Posture, and Physical Activity in Office Workers Working at Home and in Office Alternately | Wrong intervention - not mHealth |
| 511 | Shake, 2018 | Efficacy of Bingocize((R)): A Game-Centered Mobile Application to Improve Physical and Cognitive Performance in Older Adults | No full-text |
| 512 | Shang-Lin, 2020 | Effectiveness of a Home-Based Telehealth Exercise Training Program for Patients With Cardiometabolic Multimorbidity: a Randomized Controlled Trial | Wrong intervention - no resistance training in intervention |
| 513 | Shebib, 2019 | Randomized controlled trial of a 12-week digital care program in improving low back pain | Wrong intervention - no resistance training in intervention |
| 514 | Shi, 2024 | The Physical and Psychological Effects of Telerehabilitation-Based Exercise for Patients With Nonspecific Low Back Pain: Prospective Randomized Controlled Trial | Wrong outcome |
| 515 | Shin, 2017 | Enhancing physical activity and reducing obesity through smartcare and financial incentives: A pilot randomized trial | Wrong intervention - no resistance training in intervention |
| 516 | Short, 2017 | How do different delivery schedules of tailored web-based physical activity advice for breast cancer survivors influence intervention use and efficacy? | Wrong intervention - resistance training not prescribed via mHealth |
| 517 | Silarova, 2019 | Effect of communicating phenotypic and genetic risk of coronary heart disease alongside web-based lifestyle advice: The INFORM Randomised Controlled Trial | Wrong intervention - no resistance training in intervention |
| 518 | Simons, 2018 | Effect and process evaluation of a smartphone app to promote an active lifestyle in lower educated working young adults: Cluster randomized controlled trial | Wrong intervention - no resistance training in intervention |
| 519 | Simpson, 2020 | An app-, web- and social support-based weight loss intervention for adults with obesity: the 'HelpMeDoIt!' feasibility randomised controlled trial | Wrong intervention - no resistance training in intervention |
| 520 | Simsek-Cetinkaya, 2023 | Effects of a smartphone-based nursing counseling and feedback system for women with gestational diabetes on compliance, glycemic control, and satisfaction: a randomized controlled study | Wrong intervention - no resistance training in intervention |
| 521 | Sinkkonen, 2024 | The added value of remote technology and the background factors explaining the changes in biopsychosocial functioning in cardiac rehabilitation: cluster randomised controlled trial | Wrong intervention - no resistance training in intervention |
| 522 | Siriwoen, 2018 | Effectiveness of a Weight Management Program Applying Mobile Health Technology as a Supporting Tool for Overweight and Obese Working Women | Wrong intervention - resistance training not prescribed via mHealth |
| 523 | Sjoros, 2023 | The effects of a 6-month intervention aimed to reduce sedentary time on skeletal muscle insulin sensitivity: a randomized controlled trial | Wrong intervention - no resistance training in intervention |
| 524 | Sjoros, 2023 | Reducing Sedentary Time and Whole-Body Insulin Sensitivity in Metabolic Syndrome: A 6-Month Randomized Controlled Trial | Wrong intervention - no resistance training in intervention |
| 525 | Skar, 2011 | Do brief online planning interventions increase physical activity amongst university students? A randomised controlled trial | Wrong intervention - no resistance training in intervention |
| 526 | Skobel, 2017 | Internet-based training of coronary artery patients: the Heart Cycle Trial | Wrong outcome |
| 527 | Skvortsova, 2022 | Increasing the Effectiveness of a Physical Activity Smartphone Intervention With Positive Suggestions: Randomized Controlled Trial | Wrong intervention - no resistance training in intervention |
| 528 | Smith, 2016 | Web-Based Behavioral Intervention Increases Maternal Exercise but Does Not Prevent Excessive Gestational Weight Gain in Previously Sedentary Women | Wrong intervention - no resistance training in intervention |
| 529 | Sniehotta, 2019 | Behavioural intervention for weight loss maintenance versus standard weight advice in adults with obesity: A randomised controlled trial in the UK (NULevel trial) | Wrong intervention - not mHealth |
| 530 | Soetens, 1377 | Using online computer tailoring to promote physical activity: A randomized trial of text, video, and combined intervention delivery modes | Wrong intervention - no resistance training in intervention |
| 531 | Solenhill, 2016 | The effect of tailored web-based feedback and optional telephone coaching on health improvements: A randomized intervention among employees in the transport service industry | Wrong intervention - no resistance training in intervention |
| 532 | Solk, 2023 | Effect of the Fit2Thrive Intervention on Patient-reported Outcomes in Breast Cancer Survivors: A Randomized Full Factorial Trial | Wrong intervention - no resistance training in intervention |
| 533 | Sperlich, 2018 | A 4-week intervention involving mobile-based daily 6-minute micro-sessions of functional high-intensity circuit training improves strength and quality of life, but not cardio-respiratory fitness of young untrained adults | Wrong study design |
| 534 | Sporrel, 2022 | Just-in-Time Prompts for Running, Walking, and Performing Strength Exercises in the Built Environment: 4-Week Randomized Feasibility Study | Wrong intervention - no resistance training in intervention |
| 535 | Spring, 1191 | Effects of an abbreviated obesity intervention supported by mobile technology: The ENGAGED randomized clinical trial | Wrong intervention - no resistance training in intervention |
| 536 | Spring, 2013 | Integrating technology into standard weight loss treatment a randomized controlled trial | Wrong intervention - no resistance training in intervention |
| 537 | Spring, 2018 | Multicomponent mHealth Intervention for Large, Sustained Change in Multiple Diet and Activity Risk Behaviors: The Make Better Choices 2 Randomized Controlled Trial | Wrong intervention - no resistance training in intervention |
| 538 | Sriramatr, 2014 | An Internet-based intervention for promoting and maintaining physical activity: a randomized controlled trial | Wrong intervention - no resistance training in intervention |
| 539 | Staffileno, 2018 | Favorable Outcomes Using an eHealth Approach to Promote Physical Activity and Nutrition Among Young African American Women | Wrong intervention - no resistance training in intervention |
| 540 | Stahl, 2020 | Digital Monitoring of Sleep, Meals, and Physical Activity for Reducing Depression in Older Spousally-Bereaved Adults: A Pilot Randomized Controlled Trial | Wrong intervention - not mHealth |
| 541 | Stefanick, 2022 | Women's Health Initiative Strong and Healthy (WHISH): A pragmatic physical activity intervention trial for cardiovascular disease prevention | Wrong study design |
| 542 | Stenlund, 2024 | Short-term effects on physical activity level with web-based self-management support in people with COPD: a randomised controlled trial | Wrong intervention - no resistance training in intervention |
| 543 | Stephens, 2015 | Smartphone Technology and Text Messaging for Weight Loss in Young Adults: A Randomized Controlled Trial | Wrong intervention - no resistance training in intervention |
| 544 | Storm, 2016 | Effectiveness of a Web-Based Computer-Tailored Multiple-Lifestyle Intervention for People Interested in Reducing their Cardiovascular Risk: A Randomized Controlled Trial | Wrong intervention - no resistance training in intervention |
| 545 | Strom, 2013 | Internet-delivered therapist-guided physical activity for mild to moderate depression: A randomized controlled trial | Wrong intervention - no resistance training in intervention |
| 546 | Suboc, 2014 | The impact of moderate intensity physical activity on cardiac structure and performance in older sedentary adults | Wrong intervention - no resistance training in intervention |
| 547 | Suero-Pineda, 2023 | Effectiveness of a telerehabilitation tablet app in combination with face-to-face physiotherapy for people with wrist, hand or finger injuries: A pragmatic multicentre clinical trial | Wrong intervention - no resistance training in intervention |
| 548 | Suero-Pineda, 2023 | Effectiveness of a Telerehabilitation Evidence-Based Tablet App for Rehabilitation in Traumatic Bone and Soft Tissue Injuries of the Hand, Wrist, and Fingers | Wrong intervention - no resistance training in intervention |
| 549 | Suero-Pineda, 2024 | Effectiveness of a tablet telerehabilitation application in patients with distal radius fracture | Not in English |
| 550 | Sui, 2024 | Increasing physical activity among adults affected by COVID-19 social distancing restrictions: A feasibility trial of an online intervention | Wrong intervention - no resistance training in intervention |
| 551 | Sun, 2021 | Motivating Adherence to Exercise Plans Through a Personalized Mobile Health App: Enhanced Action Design Research Approach | Wrong intervention - no resistance training in intervention |
| 552 | Sweeney, 2022 | Results From "Developing Real Incentives and Volition for Exercise" (DRIVE): A Pilot Randomized Controlled Trial for Promoting Physical Activity in African American Women | Wrong intervention - no resistance training in intervention |
| 553 | Sysko, 2022 | An Initial Test of the Efficacy of a Digital Health Intervention for Bariatric Surgery Candidates | Wrong intervention - no resistance training in intervention |
| 554 | Tabak, 2020 | A Game-Based, Physical Activity Coaching Application for Older Adults: Design Approach and User Experience in Daily Life | Wrong intervention - no resistance training in intervention |
| 555 | Talebi, 2022 | Examination of influence of social media education through mobile phones on the change in physical activity and sedentary behavior in pregnant women: a randomized controlled trial | Wrong intervention - no resistance training in intervention |
| 556 | Taraldsen, 2020 | Digital Technology to Deliver a Lifestyle-Integrated Exercise Intervention in Young Seniors-The PreventIT Feasibility Randomized Controlled Trial | Wrong outcome |
| 557 | Taylor, 2020 | Adding web-based behavioural support to exercise referral schemes for inactive adults with chronic health conditions: the e-coachER RCT | Wrong study design |
| 558 | Taylor, 2020 | Randomised controlled trial of an augmented exercise referral scheme using web-based behavioural support for inactive adults with chronic health conditions: The e-coachER trial | Wrong intervention - resistance training not prescribed via mHealth |
| 559 | Taylor, 2022 | Evaluation of a Type 2 diabetes risk reduction online program for women with recent gestational diabetes: a randomised trial | Wrong intervention - no resistance training in intervention |
| 560 | Templeton, 2022 | Towards Symptom-Specific Intervention Recommendation Systems | Wrong intervention - resistance training not prescribed via mHealth |
| 561 | Teychenne, 2021 | Mums on the Move: A pilot randomised controlled trial of a home-based physical activity intervention for mothers at risk of postnatal depression | Wrong intervention - no resistance training in intervention |
| 562 | Thesen, 2022 | Effectiveness of Internet-Based Cognitive Behavioral Therapy with Telephone Support for Noncardiac Chest Pain: Randomized Controlled Trial | Wrong intervention - no resistance training in intervention |
| 563 | Thiengwittayaporn, 2021 | Development of a mobile application to improve exercise accuracy and quality of life in knee osteoarthritis patients: a randomized controlled trial | Wrong outcome |
| 564 | Thomas, 2020 | Web-based virtual reality to enhance behavioural skills training and weight loss in a commercial online weight management programme: The Experience Success randomized trial | Wrong intervention - no resistance training in intervention |
| 565 | Thorsen, 2022 | The Effects of a Lifestyle Intervention Supported by the InterWalk Smartphone App on Increasing Physical Activity Among Persons With Type 2 Diabetes: Parallel-Group, Randomized Trial | Wrong intervention - no resistance training in intervention |
| 566 | Thorsteinsen, 2014 | Increasing physical activity efficiently: An experimental pilot study of a website and mobile phone intervention | Wrong intervention - no resistance training in intervention |
| 567 | To, 2021 | Examining moderators of the effectiveness of a web- and video-based computer-tailored physical activity intervention | Wrong intervention - no resistance training in intervention |
| 568 | Todorovic, 2019 | Can social media intervention improve physical activity of medical students? | Wrong intervention - no resistance training in intervention |
| 569 | Torbjornsen, 2014 | A Low-Intensity Mobile Health Intervention With and Without Health Counseling for Persons With Type 2 Diabetes, Part 1: Baseline and Short-Term Results From a Randomized Controlled Trial in the Norwegian Part of RENEWING HEALTH | Wrong intervention - no resistance training in intervention |
| 570 | Torkhani, 2021 | Improving Health of People With Multiple Sclerosis From a Multicenter Randomized Controlled Study in Parallel Groups: Preliminary Results on the Efficacy of a Mindfulness Intervention and Intention Implementation Associated With a Physical Activity Program | Wrong intervention - not mHealth |
| 571 | Touger-Decker, 2010 | Workplace weight loss program; Comparing live and internet methods | Wrong intervention - no resistance training in intervention |
| 572 | Trukeschitz, 2884 | Exploring the effectiveness of a fitness-app prototype for home care service users in Austria and Italy | Wrong outcome |
| 573 | Turner-McGrievy, 2011 | Tweets, Apps, and Pods: Results of the 6-month Mobile Pounds Off Digitally (Mobile POD) randomized weight-loss intervention among adults | Wrong intervention - no resistance training in intervention |
| 574 | Turner-McGrievy, 2013 | Weight loss social support in 140 characters or less: Use of an online social network in a remotely delivered weight loss intervention | Wrong intervention - no resistance training in intervention |
| 575 | Upadhyay, 2022 | The Effect of Inner Engineering Online (IEO) Program on Reducing Stress for Information Technology Professionals: A Randomized Control Study | Wrong intervention - no resistance training in intervention |
| 576 | Valle, 2022 | IMPACT: A Randomized Controlled Trial of an mHealth Physical Activity Intervention for Young Adult Cancer Survivors | Wrong study design |
| 577 | Valle, 2023 | Effect of an mHealth intervention on physical activity outcomes among young adult cancer survivors: The IMPACT randomized controlled trial | Wrong intervention - no resistance training in intervention |
| 578 | van Beek, 2022 | Tablet app-based dexterity training in multiple sclerosis (TAD-MS): a randomized controlled trial | Wrong intervention - no resistance training in intervention |
| 579 | Van Dyck, 2016 | Effectiveness of the self-regulation eHealth intervention 'MyPlan1.0.' on physical activity levels of recently retired Belgian adults: a randomized controlled trial | Wrong intervention - no resistance training in intervention |
| 580 | Van Dyck, 2019 | Results of MyPlan 2.0 on Physical Activity in Older Belgian Adults: Randomized Controlled Trial | Wrong intervention - no resistance training in intervention |
| 581 | van Genugten, 2012 | Results from an online computer-tailored weight management intervention for overweight adults: randomized controlled trial | Wrong intervention - no resistance training in intervention |
| 582 | Van Horn, 2018 | Dietary Approaches to Stop Hypertension Diet and Activity to Limit Gestational Weight: Maternal Offspring Metabolics Family Intervention Trial, a Technology Enhanced Randomized Trial | Wrong intervention - no resistance training in intervention |
| 583 | van Mierlo, 2016 | Behavioral Economics, Wearable Devices, and Cooperative Games: Results From a Population-Based Intervention to Increase Physical Activity | Wrong intervention - no resistance training in intervention |
| 584 | Van Reijen, 2016 | Increasing compliance with neuromuscular training to prevent ankle sprain in sport: does the 'Strengthen your ankle' mobile App make a difference? A randomised controlled trial | Wrong outcome |
| 585 | Van Reijen, 2018 | Preventing recurrent ankle sprains: Is the use of an App more cost-effective than a printed Booklet? Results of a RCT | Wrong outcome |
| 586 | Van Woerden, 2014 | Evaluation of a web based tool to improve health behaviours in healthcare staff | Wrong intervention - no resistance training in intervention |
| 587 | Vandelanotte, 2012 | Do participants preferences for mode of delivery (text, video, or both) influence the effectiveness of a web-based physical activity intervention? | Wrong intervention - no resistance training in intervention |
| 588 | Vandelanotte, 2017 | Effectiveness of a Web 2.0 Intervention to Increase Physical Activity in Real-World Settings: Randomized Ecological Trial | Wrong intervention - no resistance training in intervention |
| 589 | Vandelanotte, 2018 | The Effectiveness of a Web-Based Computer-Tailored Physical Activity Intervention Using Fitbit Activity Trackers: Randomized Trial | Wrong intervention - not mHealth |
| 590 | Vandelanotte, 2021 | Are web-based personally tailored physical activity videos more effective than personally tailored text-based interventions? Results from the three-arm randomised controlled TaylorActive trial | Wrong intervention - no resistance training in intervention |
| 591 | Vandelanotte, 2022 | Impact of a web-based personally tailored physical activity intervention on depression, anxiety, stress and quality of life: Secondary outcomes from a randomized controlled trial | Wrong study design |
| 592 | vanden Helder, 2020 | Blended home-based exercise and dietary protein in community-dwelling older adults: a cluster randomized controlled trial | Wrong intervention - no resistance training in intervention |
| 593 | vander Kolk, 2019 | Effectiveness of home-based and remotely supervised aerobic exercise in Parkinson's disease: a double-blind, randomised controlled trial | Wrong intervention - no resistance training in intervention |
| 594 | vander Pligt, 2018 | A pilot intervention to reduce postpartum weight retention and central adiposity in first-time mothers: results from the mums OnLiNE (Online, Lifestyle, Nutrition & Exercise) study | Wrong intervention - no resistance training in intervention |
| 595 | vander Velde, 2021 | Usability and Preliminary Effectiveness of a Preoperative mHealth App for People Undergoing Major Surgery: Pilot Randomized Controlled Trial | Wrong intervention - no resistance training in intervention |
| 596 | vanderWeegen, 2015 | It's LiFe! Mobile and Web-Based Monitoring and Feedback Tool Embedded in Primary Care Increases Physical Activity: A Cluster Randomized Controlled Trial | Wrong intervention - no resistance training in intervention |
| 597 | vandeWiel, 2021 | Effects of and lessons learned from an internet-based physical activity support program (With and without physiotherapist telephone counselling) on physical activity levels of breast and prostate cancer survivors: The pablo randomized controlled trial | Wrong intervention - no resistance training in intervention |
| 598 | Vanoh, 2019 | The Effectiveness of a Web-Based Health Education Tool, WESIHAT 2.0, among Older Adults: A Randomized Controlled Trial | Wrong intervention - resistance training not prescribed via mHealth |
| 599 | Vloothuis, 2019 | Caregiver-mediated exercises with e-health support for early supported discharge after stroke (CARE4STROKE): A randomized controlled trial | Wrong intervention - no resistance training in intervention |
| 600 | Volders, 2020 | y The Effect of Active Plus, a Computer-Tailored Physical Activity Intervention, on the Physical Activity of Older Adults with Chronic Illness(es)-A Cluster Randomized Controlled Trial | Wrong intervention - no resistance training in intervention |
| 601 | vonAsh, 2024 | Pasos Hacia La Salud II: A Superiority RCT Utilizing Technology to Promote Physical Activity in Latinas | Wrong intervention - no resistance training in intervention |
| 602 | Voncken-Brewster, 2015 | A randomized controlled trial evaluating the effectiveness of a web-based, computer-tailored self-management intervention for people with or at risk for COPD | Wrong intervention - no resistance training in intervention |
| 603 | Vorrink, 2016 | A Mobile Phone App to Stimulate Daily Physical Activity in Patients with Chronic Obstructive Pulmonary Disease: Development, Feasibility, and Pilot Studies | Wrong intervention - no resistance training in intervention |
| 604 | Vroege, 2014 | Dose-response effects of a Web-based physical activity program on body composition and metabolic health in inactive older adults: additional analyses of a randomized controlled trial | Wrong intervention - no resistance training in intervention |
| 605 | Waddington, 2019 | An online intervention for promoting physical activity in rural Australian adults | Wrong study design |
| 606 | Wadsworth, 2010 | Effect of a web site intervention on physical activity of college females | Wrong intervention - no resistance training in intervention |
| 607 | Waller, 2022 | Prehabilitation with wearables versus standard of care before major abdominal cancer surgery: a randomised controlled pilot study (trial registration: NCT04047524) | Wrong intervention - resistance training not prescribed via mHealth |
| 608 | Walsh, 2016 | An mHealth Intervention Using a Smartphone App to Increase Walking Behavior in Young Adults: A Pilot Study | Wrong intervention - no resistance training in intervention |
| 609 | Wan, 2017 | Promoting physical activity in COPD: Insights from a randomized trial of a web-based intervention and pedometer use | Wrong intervention - no resistance training in intervention |
| 610 | Wan, 2020 | Long-term effects of web-based pedometer-mediated intervention on COPD exacerbations | Wrong intervention - no resistance training in intervention |
| 611 | Wang, 2014 | Mobile-phone-based home exercise training program decreases systemic inflammation in COPD: a pilot study | Wrong intervention - no resistance training in intervention |
| 612 | Wang, 2015 | Use of Facebook in physical activity intervention programme A test of self-determination theory | Wrong intervention - resistance training not prescribed via mHealth |
| 613 | Wang, 2020 | A mobile health application to support self-management in patients with chronic obstructive pulmonary disease: a randomised controlled trial | Wrong intervention - no resistance training in intervention |
| 614 | Watson, 2012 | An internet-based virtual coach to promote physical activity adherence in overweight adults: randomized controlled trial | Wrong intervention - no resistance training in intervention |
| 615 | Watson, 2015 | Effect of a web-based behavior change program on weight loss and cardiovascular risk factors in overweight and obese adults at high risk of developing cardiovascular disease: Randomized controlled trial | Wrong intervention - no resistance training in intervention |
| 616 | Welch, 2022 | User-centered development of a smartphone application (Fit2Thrive) to promote physical activity in breast cancer survivors | Wrong intervention - no resistance training in intervention |
| 617 | Weman-Josefsson, 2018 | Zooming in on the Effects: a Controlled Trial on Motivation and Exercise Behaviour in a Digital Context | Wrong intervention - no resistance training in intervention |
| 618 | Western, 2022 | Supporting Behavior Change in Sedentary Adults via Real-time Multidimensional Physical Activity Feedback: Mixed Methods Randomized Controlled Trial | Wrong intervention - no resistance training in intervention |
| 619 | Wijdenes, 2013 | Using web-based familial risk information for diabetes prevention: a randomized controlled trial | Wrong intervention - no resistance training in intervention |
| 620 | Wijsman, 2013 | Effects of a web-based intervention on physical activity and metabolism in older adults: Randomized controlled trial | Wrong intervention - no resistance training in intervention |
| 621 | Winett, 2015 | Theory-based approach for maintaining resistance training in older adults with prediabetes: adherence, barriers, self-regulation strategies, treatment fidelity, costs | Wrong intervention - not mHealth |
| 622 | Wing, 1032 | Behavioral and Cardiovascular Effects of a Behavioral Weight Loss Program for People Living with HIV | Wrong intervention - no resistance training in intervention |
| 623 | Wong, 2020 | Effects of a Web-Based Educational Support Intervention on Total Exercise and Cardiovascular Risk Markers in Adults With Coronary Heart Disease | Wrong intervention - no resistance training in intervention |
| 624 | Wu, 2020 | Collaborative Care Model Based Telerehabilitation Exercise Training Program for Acute Stroke Patients in China: A Randomized Controlled Trial | Wrong intervention - not mHealth |
| 625 | Xia, 2021 | The Physical Fitness Level of College Students Before and After Web-Based Physical Education During the COVID-19 Pandemic | Wrong intervention - no resistance training in intervention |
| 626 | Xu, 2020 | A Mobile-Based Intervention for Dietary Behavior and Physical Activity Change in Individuals at High Risk for Type 2 Diabetes Mellitus: Randomized Controlled Trial | Wrong intervention - no resistance training in intervention |
| 627 | Xu, 2023 | Smartphone-based gamification intervention to increase physical activity participation among patients with coronary heart disease: A randomized controlled trial | Wrong study design |
| 628 | Yamatsu, 2022 | Feasibility of the Remote Physical Activity Follow-Up Intervention after the Face-to-Face Program for Healthy Middle-Aged Adults: A Randomized Trial Using ICT and Mobile Technology | Wrong intervention - resistance training not prescribed via mHealth |
| 629 | Yang, 2017 | The Effects of an Activity Promotion System on active living in overweight subjects with metabolic abnormalities | Wrong intervention - no resistance training in intervention |
| 630 | Yang, 2023 | Adherence to mHealth and Paper-Based Versions of Lifestyle-Integrated Functional Exercise: A Secondary Analysis of Data From the PreventIT Feasibility Randomized Controlled Trial | Wrong outcome |
| 631 | Yildiz, 2023 | The effect of web-based and face-to-face training given to office workers on health beliefs and physical activity levels regarding obesity | Wrong intervention - no resistance training in intervention |
| 632 | Yin, 2020 | Using mobile health tools to engage rural underserved individuals in a diabetes education program in South Texas: Feasibility study | Wrong intervention - no resistance training in intervention |
| 633 | Yingling, 2016 | Community Engagement to Optimize the Use of Web-Based and Wearable Technology in a Cardiovascular Health and Needs Assessment Study: A Mixed Methods Approach | Wrong intervention - no resistance training in intervention |
| 634 | Young, 2021 | Impact of a Self-Guided, eHealth Program Targeting Weight Loss and Depression in Men: A Randomized Trial | Wrong intervention - no resistance training in intervention |
| 635 | Yu, 2017 | Impact of a workplace physical activity tracking program on biometric health outcomes | Wrong study design |
| 636 | Yuan, 2022 | My Wellness Coach: evaluation of a mobile app designed to promote integrative health among underserved populations | Wrong intervention - no resistance training in intervention |
| 637 | Yudi, 2021 | SMARTphone-based, early cardiac REHABilitation in patients with acute coronary syndromes: A randomized controlled trial | Wrong intervention - no resistance training in intervention |
| 638 | Yun, 2020 | Efficacy of health coaching and a web-based program on physical activity, weight, and distress management among cancer survivors: A multi-centered randomised controlled trial | Wrong intervention - no resistance training in intervention |
| 639 | Zhang, 2015 | Efficacy and causal mechanism of an online social media intervention to increase physical activity: Results of a randomized controlled trial | Wrong intervention - resistance training not prescribed via mHealth |
| 640 | Zhang, 2018 | Complexity of Daily Physical Activity Is More Sensitive Than Conventional Metrics to Assess Functional Change in Younger Older Adults | Wrong intervention - resistance training not prescribed via mHealth |
| 641 | Zhang, 2019 | Mobile App-Based Small-Group Physical Activity Intervention for Young African American Women: a Pilot Randomized Controlled Trial | Wrong intervention - no resistance training in intervention |
| 642 | Zhang, 2021 | Effects of online bodyweight high-intensity interval training intervention and health education on the mental health and cognition of sedentary young females | Wrong intervention - not mHealth |
| 643 | Zhou, 2021 | Effect of Mobile-Based Lifestyle Intervention on Weight Loss among the Overweight and Obese Elderly Population in China: A Randomized Controlled Trial | Wrong intervention - not mHealth |
| 644 | Zoellner, 2016 | The Influence of Health Literacy on Reach, Retention, and Success in a Worksite Weight Loss Program | Wrong intervention - no resistance training in intervention |
| 645 | Zongpa, 2020 | Effectiveness of A Smartphone Directed Physical Activity Program on Cardiometabolic Disease Risk in Desk-Based Office Employees -- A Pragmatic, Two-Arm, Parallel, Cluster Randomised Trial | Wrong intervention - no resistance training in intervention |
