# Supplementary material for: Effects of mHealth interventions to prescribe resistance training: a systematic review and meta-analysis of randomized controlled trials
Source: Int J Behav Nutr Phys Act. 2025 Dec 22;23:7. doi: 10.1186/s12966-025-01868-8 (PMC12836956; doi:10.1186/s12966-025-01868-8)
Supplement: Supplementary file 6 — Supplementary Material 6. [file 12966_2025_1868_MOESM6_ESM.docx]

| **Effects of mHealth Interventions to Prescribe Resistance Training: A Systematic Review and Meta-Analysis of Randomized Controlled Trials**  **Sports Medicine**  Emily R. Cox, Sam Beacroft, Anna K. Jansson, Levi Wade, Mitch J. Duncan, David R. Lubans, Sara L. Robards, Manuel Leitner, Niklas Gutberlet, Ronald C. Plotnikoff^*^  Ron Plotnikoff, [ron.plotnikoff@newcastle.edu.au](mailto:ron.plotnikoff@newcastle.edu.au)  ATC314*,* University Drive*,* Callaghan, NSW 2308*,* Australia  Tel: +61 (02) 49854465  Fax: 61 + 2 49212084  **TABLE S4. Behaviour change techniques identified in interventions** | | | | | | | | | | | | | | | | | | | | | | | | | | |
| --- | --- | --- | --- | --- | --- | --- | --- | --- | --- | --- | --- | --- | --- | --- | --- | --- | --- | --- | --- | --- | --- | --- | --- | --- | --- | --- |
|  | **Provide information about behaviour health link** | **Provide information on consequences** | **Provide information about others’ approval** | **Prompt intention formation** | **Prompt barrier identification** | **Provide general encouragement** | **Set graded tasks** | **Provide instruction** | **Model or demonstrate the behaviour** | **Prompt specific goal setting** | **Prompt review of behavioural goals** | **Prompt self-monitoring of behaviour** | **Provide feedback on performance** | **Provide contingent rewards** | **Teach to use prompts or cues** | **Agree on beahvioural contract** | **Prompt practice** | **Use follow-up prompts** | **Provide opportunities for social comparison** | **Plan social support or social change** | **Prompt identificaiton as a role model** | **Prompt self-talk** | **Relapse prevention** | **Stress management** | **Motivational interviewing** | **Time management** |
| Alasfour (2020) |  |  |  |  |  | x | x | x | x |  |  |  |  |  |  |  | x |  |  |  |  |  |  |  |  |  |
| Chan (2022) | x | x |  |  |  |  |  | x | x | x |  | x |  |  |  |  | x | x |  |  |  |  |  |  |  |  |
| Dieter (2024) |  |  |  |  |  |  | x | x | x |  |  |  | x |  |  |  | x |  |  |  |  |  |  |  |  |  |
| Donkers (2020) |  |  |  |  |  |  | x | x | x |  |  | x |  |  |  |  |  |  |  |  |  |  |  |  |  |  |
| Ehling (2017) |  |  |  |  |  | x |  | x | x |  |  |  |  |  |  |  | x |  |  |  |  |  |  |  |  |  |
| Frevel (2015) |  |  |  |  |  |  | x | x |  |  |  |  |  |  |  |  |  |  |  |  |  |  |  |  |  |  |
| Gohir (2021) |  | x |  |  |  |  | x | x |  |  |  |  |  |  |  |  | x |  |  |  |  |  |  |  |  |  |
| Granet (2023) |  |  |  |  |  |  | x | x | x |  |  |  |  |  |  |  |  |  |  |  |  |  |  |  |  |  |
| Hansen (2012) |  | x |  |  |  |  |  | x |  | x |  | x | x |  |  |  |  |  |  | x |  |  | x |  |  |  |
| Hawley-Hague (2023) |  | x |  |  |  | x | x | x | x | x | x |  | x |  |  |  | x |  |  |  |  |  |  |  |  | x |
| Irvine (2013) |  | x |  |  | x | x | x | x | x | x |  | x |  |  |  |  |  |  |  |  |  |  |  |  |  | x |
| Jungreitmayr (2022) |  |  |  |  |  | x | x | x |  |  |  |  | x |  |  |  |  |  |  |  |  |  |  |  |  |  |
| Lee (2019) |  | x |  |  |  |  | x |  | x |  |  |  | x |  |  |  |  |  |  |  |  |  |  |  |  |  |
| Lee (2022) |  |  |  |  |  |  |  | x | x |  |  |  |  |  |  |  |  |  |  | x |  |  |  |  |  |  |
| Li (2021) |  |  |  |  |  |  | x | x | x |  |  |  | x |  |  |  |  |  |  |  |  |  |  |  |  |  |
| Li (2022) |  |  |  |  |  |  | x | x | x |  |  |  |  |  |  |  |  |  |  |  |  |  |  |  |  |  |
| Moutzouri (2024) |  | x |  |  |  | x |  | x | x |  |  |  |  |  |  |  |  |  |  |  |  |  |  |  |  |  |
| Muntaner-Mas (2021) |  |  |  |  |  |  |  | x | x | x | x | x | x |  |  |  |  |  |  |  |  |  |  |  |  |  |
| Nabutovsky (2024) |  | x |  |  |  |  | x | x | x |  |  |  |  |  |  |  | x |  |  | x |  |  |  |  |  |  |
| Nasseri (2020) |  |  |  |  |  |  |  | x |  |  |  |  | x |  |  |  |  |  |  |  |  |  |  |  |  |  |
| Park (2021) |  |  |  |  |  |  | x | x | x |  |  |  |  |  |  |  |  |  |  |  |  |  |  |  |  |  |
| Plotnikoff (2023) |  | x |  |  |  |  | x | x | x | x |  | x |  |  |  |  |  |  | x | x |  |  |  |  |  |  |
| Prieto-Moreno (2024) |  | x |  |  |  |  | x | x | x |  |  |  |  |  |  |  |  |  |  | x |  |  |  |  |  |  |
| Rees-Punia (2022) |  | x |  |  |  |  |  | x | x | x |  |  |  |  |  |  | x |  |  | x |  |  |  |  |  |  |
| Spielmanns (2022) |  |  |  |  |  |  | x | x | x |  |  |  |  |  |  |  | x |  |  |  |  |  |  |  |  |  |
| Stork (2021) |  |  |  |  |  |  | x | x | x |  |  |  |  |  |  |  |  |  |  |  |  |  |  |  |  |  |
| Tallner (2016) |  |  |  |  |  |  | x | x |  |  |  |  |  |  |  |  |  |  |  |  |  |  |  |  |  |  |
| Tanhan (2024) |  |  |  |  |  |  | x |  | x |  |  |  |  |  |  |  |  |  |  |  |  |  |  |  |  |  |
| Timurtas (2022) |  |  |  |  |  | x | x | x | x |  |  |  |  |  |  |  | x |  |  | x |  |  |  |  |  |  |
| Weber (2024) |  | x |  |  |  |  | x | x | x | x |  |  |  |  |  |  | x |  |  |  |  |  |  |  |  |  |
| Yasuhiro (2024) |  |  |  |  |  |  |  | x | x |  |  |  |  |  |  |  |  |  |  |  |  |  |  |  |  |  |
| Ziebart (2024) | x | x |  |  |  |  | x | x | x |  |  | x |  |  |  |  |  |  |  |  |  |  |  |  |  |  |
