# Supplementary material for: Effects of mHealth interventions to prescribe resistance training: a systematic review and meta-analysis of randomized controlled trials
Source: Int J Behav Nutr Phys Act. 2025 Dec 22;23:7. doi: 10.1186/s12966-025-01868-8 (PMC12836956; doi:10.1186/s12966-025-01868-8)
Supplement: Supplementary file 7 — Supplementary Material 7. [file 12966_2025_1868_MOESM7_ESM.docx]

**TABLE S3. Meta-analysis results**

| **Analysis** | **Reference** | **Intercept *d* [95 % CI], *p*** | **Moderator effect(s)** | **R²₃** | **Q (df), *p*** | **I^2^_3_ (%)** | ***k* / *n*** |
| --- | --- | --- | --- | --- | --- | --- | --- |
| **Overall** |  |  |  |  |  |  |  |
| Overall pooled | — | 0.18 [0.08, 0.28],  p < .001 | — | — | 61.64 (40),  p = .02 | 28.9 | 18 / 41 |
| Population | Healthy | 0.26 [0.03, 0.49],  p = .03 | Clinical –0.11 [–0.36, 0.14], p = .39 | .00 | 61.64 (40),  p = .02 | — | 18 / 41 |
| Intervention duration | ≤ 12 weeks | 0.18 [0.03, 0.32],  p = .02 | >12 wks –0.006 [–0.20, 0.19], p = .95 | .00 | 61.64 (40),  p = .02 | — | 18 / 41 |
| mHealth mode | App | 0.17 [0.05, 0.29],  p = .01 | Website +0.12 [–0.19, 0.43], p = .46 | .00 | 61.43 (38),  p = .01 | — | 17 / 39 |
| Dosing | Fixed prescription | 0.37 [0.08, 0.66],  p = .01 | Std prog –0.27 [–0.57, 0.03], p = .08; Indiv prog –0.18 [–0.50, 0.15], p = .29 | .22 | 43.76 (33),  p = .10 | — | 13 / 34 |
| Combined  moderators | ≤ 12 wks; App; Fixed | 0.28 [0.08, 0.49],  p = .01 | Length –0.09 [–0.33, 0.14], p = .45; Website +0.42 [–0.06, 0.89], p = .09; Std prog –0.19 [–0.42, 0.04], p = .11 | .86 | 42.91 (31),  p = .08 | — | 12 / 32 |
| **Lower body** |  |  |  |  |  |  |  |
| Lower body pooled | — | 0.22 [0.11, 0.33],  p < .001 | — | — | 31.84 (26),  p = .20 | 13 | 15 / 27 |
| Population | Healthy | 0.29 [–0.02, 0.61],  p = .07 | Clinical –0.09 [–0.43, 0.25], p = .60 | .00 | 31.84 (26),  p = .20 | — | 15 / 27 |
| Intervention duration | ≤ 12 weeks | 0.22 [0.07, 0.38],  p = .01 | >12 wks –0.02 [–0.25, 0.21], p = .88 | .00 | 31.84 (26),  p = .20 | — | 15 / 27 |
| mHealth mode | App | 0.22 [0.09, 0.34],  p < .001 | Website +0.49 [–0.16, 1.13], p = .14 | .33 | 30.88 (24),  p = .16 | — | 14 / 25 |
| Dosing | Fixed prescription | 0.36 [0.09, 0.64],  p = .01 | Std prog –0.28 [–0.55, –0.02], p = .04; Indiv prog –0.12 [–0.42, 0.17], p = .41 | 1.00 | 26.31 (22),  p = .24 | — | 11 / 23 |
| Outcome sub-type | Strength | 0.21 [0.05, 0.37],  p = .01 | Strength endurance +0.20 [0.04, 0.37], p = .02 | .00 | 21.88 (17),  p = .19 | — | 14 / 18 |
| **Upper body** |  |  |  |  |  |  |  |
| Upper body pooled | — | 0.11 [–0.04, 0.26],  p = .15 | — | — | 25.54 (13),  p = .02 | 35 | 10 / 14 |
| Intervention duration | ≤ 12 weeks | 0.05 [–0.16, 0.25],  p = .65 | >12 wks +0.08 [–0.14, 0.30], p = .48 | .16 | 25.54 (13),  p = .02 | — | 10 / 14 |

Intercept (Cohen’s *d*) and 95 % CIs describe the effect in the reference (0) category. Moderator effects are contrasts relative to the reference group. *R*²₃ = proportion of Level-3 variance explained by the moderator; *Q*(df) = Cochran’s *Q* for residual heterogeneity (df = number of effects – number of parameters); *I*²₃ (%) = percentage of total variance attributable to between‐study heterogeneity (only shown for primary models); *k* = number of studies, *n* = number of effect sizes.
